# *MEF2B* mutations in non-Hodgkin lymphoma dysregulate cell migration by decreasing MEF2B target gene activation

**DOI:** 10.1038/ncomms8953

**Published:** 2015-08-06

**Authors:** Julia R. Pon, Jackson Wong, Saeed Saberi, Olivia Alder, Michelle Moksa, S. -W. Grace Cheng, Gregg B. Morin, Pamela A. Hoodless, Martin Hirst, Marco A. Marra

**Affiliations:** 1 Canada's Michael Smith Genome Sciences Centre, BC Cancer Agency, Vancouver, Canada V5Z 1L3; 2 Department of Microbiology and Immunology, Centre for High-Throughput Biology, University of British Columbia, Vancouver, Canada V6T 1Z4; 3 Terry Fox Laboratory, BC Cancer Agency, Vancouver, Canada V5Z 1L3; 4 Department of Medical Genetics, University of British Columbia, Vancouver, Canada V6T 1Z3

## Abstract

Myocyte enhancer factor 2B (MEF2B) is a transcription factor with mutation hotspots at K4, Y69 and D83 in diffuse large B-cell lymphoma (DLBCL). To provide insight into the regulatory network of MEF2B, in this study, we analyse global gene expression and DNA-binding patterns. We find that candidate MEF2B direct target genes include *RHOB*, *RHOD, CDH13*, *ITGA5* and *CAV1*, and that indirect target genes of MEF2B include *MYC*, *TGFB1*, *CARD11*, *MEF2C*, *NDRG1* and *FN1*. MEF2B overexpression increases HEK293A cell migration and epithelial–mesenchymal transition, and decreases DLBCL cell chemotaxis. K4E, Y69H and D83V *MEF2B* mutations decrease the capacity of MEF2B to activate transcription and decrease its' effects on cell migration. The K4E and D83V mutations decrease MEF2B DNA binding. In conclusion, our map of the MEF2B regulome connects MEF2B to drivers of oncogenesis.

## Abstract (web summary)

Mutations in the transcription factor *MEF2B* are found in diffuse large B-cell lymphoma. In this study, the authors map the DNA-binding sites of the transcription factor in cells *in vitro* and find that the mutations decrease the ability of MEF2B to activate transcription.

## Graphical abstract

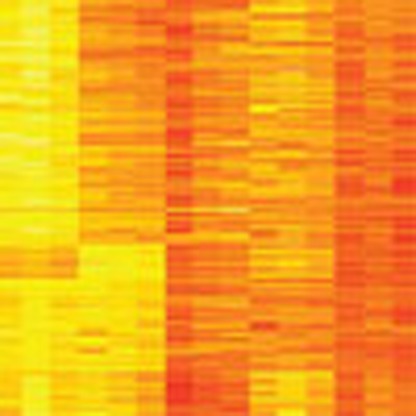

MEF2 proteins are transcription factors involved in the regulation of muscle, neural crest, endothelial cell, chondrocyte, neuron and lymphocyte development^1^. The four human MEF2 proteins, MEF2A, MEF2B, MEF2C and MEF2D, consist of an N-terminal DNA-binding MADS domain, a central MEF2 domain, and a C-terminal transcriptional activation domain^1^. Two isoforms of myocyte enhancer factor 2B (MEF2B) have been described, isoforms A and B, which differ in their transcriptional activation domains^2^. MEF2B is the most divergent of the MEF2 proteins^3^, with neither isoform sharing >31% amino-acid identity with any other MEF2 protein. MEF2B's target gene specificity also appears divergent from that of its paralogues. For instance, MEF2B does not regulate immunoglobulin J-chain gene expression like other MEF2 proteins^4^, and is the only MEF2 protein to bind a promoter region required for maintaining *SMHC* expression^5^. Genome scale technologies have been applied for identifying target genes of MEF2A^6^,^7^,^8^ and MEF2C^8^, but not MEF2B. The only suggested MEF2B direct target genes are *SMHC*^5^ (a smooth muscle myosin gene), *BZLF1* (ref. 9; involved in Epstein–Barr virus reactivation), *SOST*^10^ (a Wnt inhibitor) and *BCL6* (ref. 2; a transcriptional regulator in B-cells).

*MEF2B* is amplified in 9% of ovarian carcinomas (28 out of 311 cases, TCGA provisional data^11^,^12^), 5% of uterine carcinomas (11 out of 240 cases^13^), 5% of adrenocortical carcinomas (4 out of 88 cases, TCGA provisional data^11^,^12^) and 3% of oesophageal carcinomas (6 out of 184 cases, TCGA provisional data^11^,^12^), indicating that MEF2B may act as an oncogene in these carcinomas. Moreover, *MEF2B* is the target of heterozygous somatic mutations in 8–18% of diffuse large B-cell lymphoma (DLBCL)^14^,^15^,^16^, 13% of follicular lymphoma^14^ and 3% of mantle cell lymphoma^17^. Mutations in other *MEF2* genes were either not detected in these lymphomas or were much less frequent^11^,^12^.

Out of 69 *MEF2B* mutations in DLBCL, 27 affected D83, 6 affected Y69 and 6 affected K4 (ref. 14). K4, Y69 and D83 are located in the MADS and MEF2 domains, domains that in MEF2C were required fordimerization and DNA binding^18^. Three to 22% of *MEF2B* mutations in DLBCL^14^,^15^ and 33% of *MEF2B* mutations in follicular lymphoma^14^ were present in the transcriptional activation domain, consisting predominantly of nonsense, frameshift, splice-site or stop codon read-through mutations. Two DLBCL cases with homozygous MEF2B deletion have also been identified^11^,^12^. Expression of the only MEF2B target gene identified in B-cells, *BCL6*, was not affected by the K4E *MEF2B* mutation and could not rescue MEF2B knockdown cells from cell cycle arrest^2^, indicating that there are other target genes through which *MEF2B* mutations may promote lymphomagenesis.

In this study, we profile the genome-wide distribution of wild type (WT) and mutant MEF2B binding sites and assess the transcriptome-wide impacts of WT and mutant MEF2B on gene expression. We identify direct and indirect candidate target genes of MEF2B and associate these with changes in cell proliferation, survival, migration and epithelial–mesenchymal transition (EMT). We also describe effects of *MEF2B* mutation on both DLBCL cell chemotaxis and the expression of lymphoma driver genes. Our data indicate that *MEF2B* mutations decrease target gene activation and alter cell migration.

## Results

### Identification of MEF2B target genes

To identify MEF2B target genes, we analysed microarray data from HEK293A cells stably transfected with WT V5-tagged MEF2B (Supplementary Fig. 1a) and untransfected cells. The 3,944 differentially expressed genes (DEGs) that were found between WT MEF2B-V5 and untransfected cells (Benajmini–Hochberg (B–H) adjusted eBayes *P* values <0.05, Supplementary Data 1) are potential MEF2B target genes^19^. We verified the DEGs using whole-transcriptome sequencing (RNA-seq) data from the MEF2B-V5 cells and cells stably transfected with an empty vector, the latter to control for effects of transfection and selection. Gene expression changes in RNA-seq and microarray data correlated well (Spearman correlation 0.64; Supplementary Fig. 1b; Supplementary Data 2).

We next sought to validate the differential expression of 30 genes with functions of particular interest. The 30 genes were selected to represent the two functional annotation categories that Ingenuity Pathway Analysis (IPA) indicated were most enriched in the microarray DEGs (B–H adjusted right-tailed Fisher exact test *P* values <2 × 10^−10^; Supplementary Fig. 2a): ‘cellular movement' and ‘cellular growth and proliferation'. These categories were also enriched in the RNA-seq DEGs (B–H adjusted right-tailed Fisher exact test *P* values <0.002, Supplementary Fig. 2b), supporting the prediction that these processes were affected by MEF2B-V5 expression.

**Figure 1:**
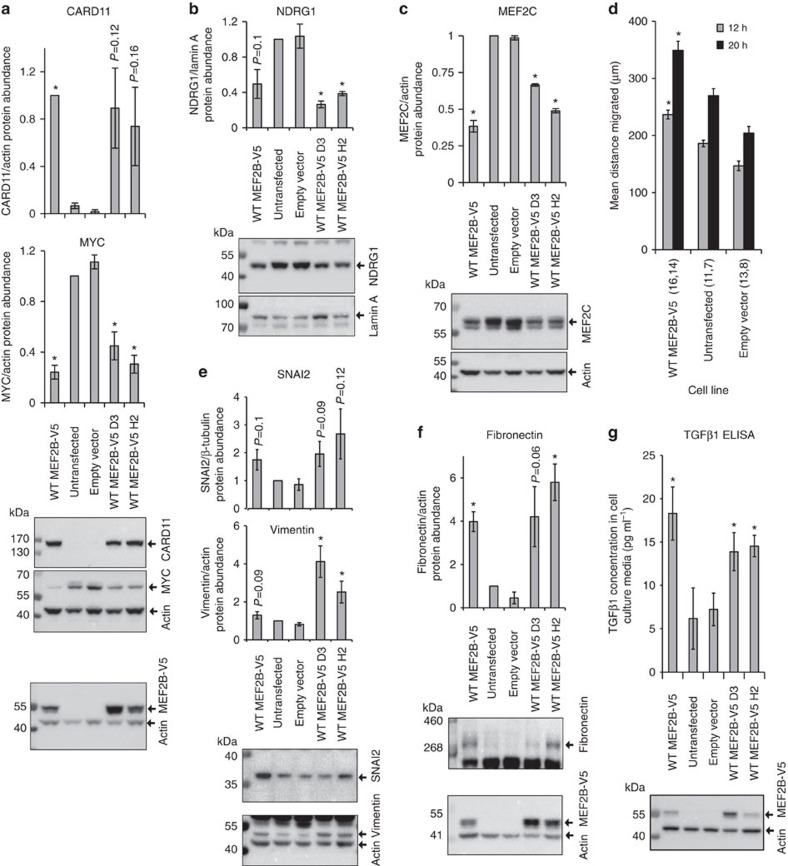
MEF2B-V5 expression alters the abundance of protein from target genes and increases HEK293A cell migration. (**a**–**c**) CARD11, MYC, NDRG1 and MEF2C protein abundance is affected by MEF2B-V5 expression. CARD11 and MYC are shown in the same panel as they were detected on the same blots. The MEF2B-V5 bands shown in **a** were detected in the same lysates as for western blots shown in **a**–**c**. (**d**) MEF2B-V5 expression increases movement into the scratched area of a confluent monolayer. The total number of scratches assessed for each sample is shown in parentheses for 12 and 20 h time points, respectively. (**e**,**f**) Abundance of the mesenchymal proteins SNAI2, vimentin and fibronectin is increased by MEF2B-V5 expression. The MEF2B-V5 bands shown in **f** were detected in the same lysates as for western blots shown in **e**,**f**. (**g**) The concentration of TGFβ1 in cell culture media was increased by MEF2B-V5 expression. TGFβ1 concentration was assessed using a Quantikine ELISA assay (R&D Systems). Shown is the mean of four biological replicates. The western blot indicates MEF2B-V5 abundance in cells whose media was assayed for TGFβ1. For all panels, *P<0.05 in comparison with empty vector cells (Student's two-tailed *t*-test, unpaired). Error bars represent the s.e.m. Relative protein abundance was calculated compared with untransfected cells except for CARD11 abundance, which was calculated compared with WT MEF2B-V5 cells. Western blots and densitometry were performed on four biological replicates of WT MEF2B-V5 and untransfected cells and two biological replicates of empty vector, WT MEF2B-V5 D3 and WT MEF2B-V5 H2 cells. Representative western blots are shown. MEF2B-V5 was detected using V5 antibody. The WT MEF2B-V5 cell line was the monoclonal cell line used for microarrays. WT MEF2B-V5 D3 and H2 were monoclonal cell lines different from the cell line used for microarray. All WT MEF2B-V5 cell lines were HEK293A cells stably transfected with WT MEF2B-V5.

Validation was performed using quantitative PCR with reverse transcription (qRT–PCR) on empty vector cells compared with two monoclonal HEK239A cell lines stably expressing WT MEF2B-V5 (referred to as WT MEF2B-V5 H2 and WT MEF2B-V5 D3) that were different from the WT MEF2B-V5 cell line used for microarrays (Supplementary Fig. 3a). Differential expression of 27 out of the 30 validation set genes was validated in at least one of the two additional WT MEF2B-V5 lines compared with empty vector cells (Supplementary Fig. 3b,c; Supplementary Table 1). The differential expression of a previously identified MEF2B target gene, *BCL6* (ref. 2), was validated as a positive control. Three of the other validated genes, *CARD11, MYC* and *NDRG1*, were among the top 50 genes when all genes were ranked by fold change in expression using microarray data. Increased abundance of CARD11 protein in MEF2B-V5 versus control cells was validated, as was the decreased abundance of MYC and NDRG1 proteins (Fig. 1a,b; Supplementary Fig. 4a,b). Also validated at the protein level was *MEF2C*, the only MEF2 family gene other than *MEF2B* itself that was differentially expressed in the microarray data (B–H adjusted eBayes *P* value 0.03; Fig. 1c; Supplementary Fig. 4c).

The remaining 25 validation set genes had functions related to cell migration. Consistent with the differential expression of migration regulators and with IPA predictions of increased cellular movement of WT MEF2B-V5 versus untransfected cells (Supplementary Data 3; Supplementary Table 2), WT MEF2B-V5 cells filled scratched areas of a confluent monolayer faster than control cells (Fig. 1d). Faster scratch closure was likely due to increased cell migration, not increased proliferation, as no increase in proliferation was detected between MEF2B-V5 and control cells (Supplementary Fig. 5).

Interestingly, a set of 67 genes whose expression increased during EMT^20^ tended to have greater expression in MEF2B-V5 cells than in untransfected cells (gene set enrichment analysis^21^,^22^ false discovery rate 0.105; Supplementary Fig. 6). Among these genes were the well-known EMT inducers *TGFB1*, *FOXC2* and *SNAI2* (also called *SLUG*) as well as the mesenchymal markers *VIM* (encoding vimentin) and *FN1* (encoding fibronectin)^23^. As expected from the microarray data, SNAI2, vimentin, fibronectin and transforming growth factor β1 (TGFβ1) proteins tended to have greater abundance in the two additional MEF2B-V5 lines than in empty vector and untransfected cells (Fig. 1e–g; Supplementary Fig. 4d–f). Interestingly, IPA identified *TGFB1* as the transcriptional regulator whose known set of target genes overlapped most significantly with the microarray DEGs (Fisher's exact test *P* value 1 × 10^−13^, activation z-score 7.0), consistent with the notion that increased TGFβ signalling mediated a large proportion of the gene expression changes downstream of MEF2B. Further supporting this notion, the same prediction was made using DEGs identified from RNA-seq data (Fisher's exact test *P* value 7 × 10^−5^, activation z-score 3.2).

### Identification of genome-wide MEF2B-binding sites

**Figure 2:**
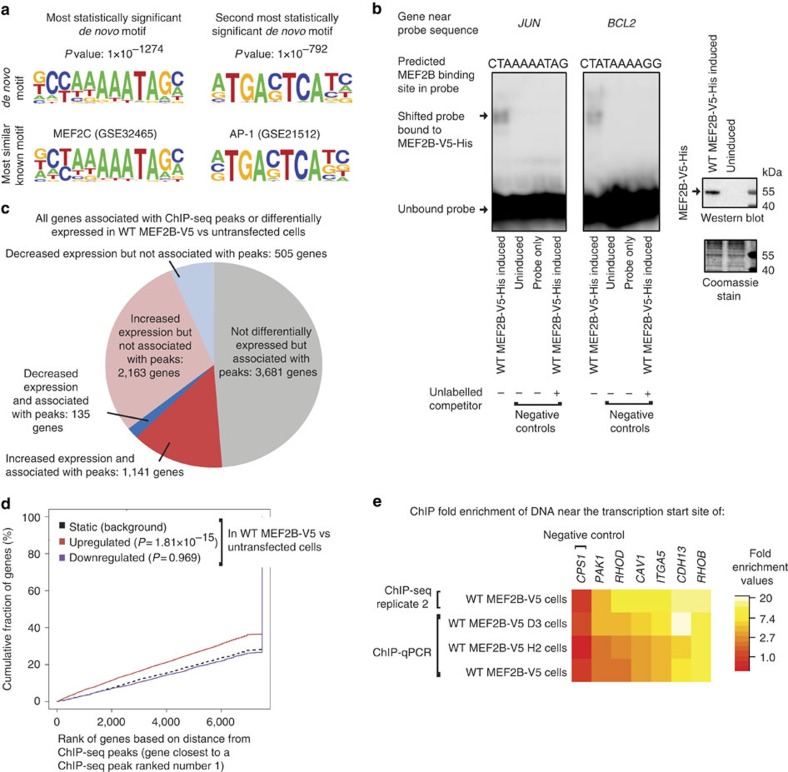
MEF2B binds MEF2 motifs and acts as a transcriptional activator. (**a**) MEF2 and AP-1 motifs were identified as the most enriched motifs, using ChIPseek's implementation of HOMER^24^. (**b**) Gel-shift assays indicated that MEF2B-V5-His binds sequences similar to MEF2 motifs. Probes contained sequence located within 5 kb of the TSS of the indicated gene. The unlabelled competitor consisted of the same sequence as the labelled probe. Lysates were from *E. coli* with or without induction of *MEF2B-V5-His* expression. (**c**) The list of genes associated with ChIP-seq peaks overlaps with the list of DEGs in WT MEF2B-V5 versus untransfected cells. (**d**) The genes whose TSSs tended to be closest to ChIP-seq peaks were those that had increased expression in WT MEF2B-V5 versus untransfected cells. The *x* axis indicates rank numbers based on the distance between the genes' TSSs and ChIP-seq peaks. The *y* axis indicates the proportion of genes with ranks at or better than the *x* axis value. Rankings were calculated and plotted using BETA^28^. *P* values were calculated compared with the background distribution (one-tailed Kolmogorov–Smirnov test). For **c** and **d**, ChIP-seq peaks were identified at a MACS2 (ref. 29) false discovery rate of 0.05, and DEGs had B–H adjusted eBayes *P* values <0.05. Only peaks identified in both ChIP-seq replicates were considered. (**e**) ChIP–qPCR validation of ChIP-seq peaks. ChIP–qPCR fold enrichments were calculated compared with enrichment of an intergenic region not expected to interact with MEF2B, then normalized to fold enrichment in ChIP–qPCR using normal immunoglobulin. Shown are the means of three biological replicates.

The identified DEGs are likely to include both direct and indirect target genes of MEF2B. To identify candidate MEF2B direct target genes, we first identified genome-wide MEF2B-binding sites using a V5 antibody for chromatin immunoprecipitation sequencing (ChIP-seq; Supplementary Table 3; Supplementary Fig. 7; Supplementary Data 4). We used the ChIPseek^24^ implementation of HOMER to identify *de novo* motifs significantly enriched in our ChIP-seq data (Fig. 2a; Supplementary Table 4). The most enriched *de novo* motif matched the known motif of MEF2C and was most enriched near the centres of peaks (CentriMo^25^ central enrichment *P* value <1 × 10^−10^; Supplementary Fig. 8), consistent with the idea that it is directly bound by MEF2B. Binding of MEF2B to sequences similar to MEF2 motifs was validated using gel-shift assays (Fig. 2b; Supplementary Fig. 9).

A MEF2C motif was present in 37% of peaks (Supplementary Data 5). The second most enriched motif, present in 30% of peaks, was that of the AP-1 complex (Fig. 2a; Supplementary Data 5). MEF2 motifs and the AP-1 complex motif tended to co-occur (ChIPModule^26^ corrected *P* value 1.33 × 10^−8^; 12.8% of peaks with MEF2 motifs also contained AP-1 motifs), consistent with the notion that MEF2B and the AP-1 complex may cooperatively regulate target genes. Interestingly, ENCODE data indicated that the AP-1 complex motif was also enriched in MEF2A and MEF2C ChIP-seq peak regions^8^, indicating that MEF2A and MEF2C may also interact with the AP-1 complex.

### Identification of candidate MEF2B direct target genes

We next used GREAT^27^ to identify 4,957 genes potentially regulated by the regions with peaks in both ChIP-seq replicates (Supplementary Fig. 10). The overlap between peak associated genes and DEGs was greater than expected by chance (*χ*^2^ with Yates correction *P* value <0.0001, Fig. 2c), supporting that expression of MEF2B-V5 can alter expression of genes associated with its binding sites. The 2,668 DEGs that were not associated with peaks may be indirect target genes.

Consistent with the hypothesis that MEF2B acts as a transcriptional activator, 89% of DEGs associated with peaks had increased expression in WT MEF2B-V5 cells compared with untransfected cells (Fig. 2c). Assuming that genes closer to ChIP-seq peak regions are more likely to truly be regulated by the peak regions^28^, the differential expression of genes closest to ChIP-seq peaks is likely to be the best indicator of MEF2B's effects. Supporting that MEF2B tends to act as an activator, MEF2B peak regions tended to be closer to genes with increased expression in WT MEF2B-V5 versus untransfected cells than to other genes (Fig. 2d).

Assuming that all peak-associated genes with increased expression in MEF2B-V5 versus untransfected cells (B–H adjusted eBayes *P* values <0.05) are candidate MEF2B direct target genes, our data indicated that 1,141 genes are candidate MEF2B direct target genes (Supplementary Data 6 and the red section of Fig. 2c). To estimate the likelihood that each gene was not a true direct target, we used rank product scores^28^. Lower, more significant rank product scores were assigned to genes with greater fold changes in expression and with transcription start sites (TSSs) closer to ChIP-seq peaks^28^. Rank product scores for 821 of the candidate direct target genes were <0.05, indicating that those 821 genes may be considered high confidence candidate direct target genes.

We selected peaks for validation that were near the 27 genes whose differential expression had already been validated. Six of the 27 genes (that is, *RHOB*, *CDH13*, *ITGA5*, *CAV1*, *RHOD* and *PAK1*) had peaks within 5 kb of their TSSs and were thus considered particularly high confidence direct target genes. Peak regions near all but *PAK1* showed at least twofold enrichment in V5 chromatin immunoprecipitation quantitative PCR (ChIP–qPCR) in at least two of the three WT MEF2B-V5 lines and were thus considered to be validated (Fig. 2e; Supplementary Fig. 11).

### MEF2B target genes regulate cell movement and survival

To predict which of the candidate direct target genes might contribute to the differences in cellular movement observed between WT MEF2B-V5 and control cells, we used IPA to analyse the functional annotations of the 1,141 candidate direct target genes. ‘Cellular movement' and ‘cell growth and proliferation' were the two most enriched categories in the candidate direct targets (B–H adjusted right-tailed Fisher exact test *P* value 8 × 10^−23^; Supplementary Fig. 12; Supplementary Data 7) as they were in the set of all DEGs (that is, both direct and indirect target genes).

The third most enriched category in the candidate direct targets was ‘cell death and survival', containing predominantly prosurvival and antiapoptotic genes (B–H adjusted right-tailed Fisher exact test *P* value 3 × 10^−16^; Supplementary Data 7). MEF2B knockdown has been associated with decreased cell cycle progression in DLBCL cells^2^, consistent with the notion that MEF2B activity helps maintain cell viability. However, no differences in proliferation were detected between WT MEF2B-V5 and control cells, perhaps because endogenous factors maintained cell proliferation and survival at levels insensitive to further increases.

Given the evidence implicating MEF2B in the maintenance of cell viability, we inspected the genes associated with MEF2B-V5 ChIP-seq peaks for well-known regulators of cell viability. We noted that the antiapoptotic lymphoma proto-oncogene *BCL2* contained a MEF2B ChIP-seq peak in its first intron (MACS2 (ref. 29) false discovery rate 1 × 10^−17^; Supplementary Fig. 13a) and had increased expression in WT MEF2B-V5 versus untransfected cells in microarray data (B–H adjusted eBayes *P* value 0.005). Similarly, the proto-oncogene and cell cycle regulator *JUN* was associated with a MEF2B ChIP-seq peak overlapping its TSS (MACS2 (ref. 29) false discovery rate 1 × 10^−67^ in replicate 2; Supplementary Fig. 13a). *JUN* expression is known to be regulated by MEF2 family proteins other than MEF2B^30^,^31^. However, *JUN* was not differentially expressed in WT MEF2B-V5 versus control cells, perhaps because endogenous factors maintained expression of *JUN* at a level insensitive to further activation. To validate *BCL2* and *JUN* as candidate MEF2B direct target genes, we first used ChIP–qPCR to validate ChIP-seq peak regions near these genes (Supplementary Fig. 13b). We also used gel-shift assays to demonstrate that MEF2B directly binds sequences within the validated peak regions (Fig. 2b).

### *MEF2B* mutations reduce MEF2B transcriptional activity

**Figure 3:**
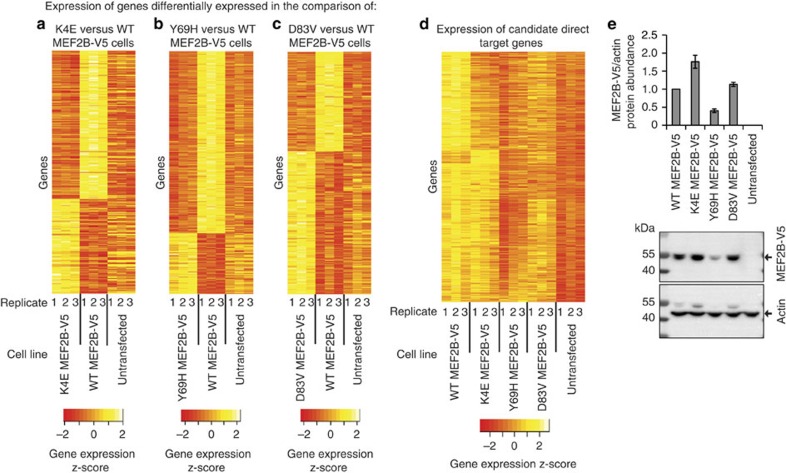
Expression microarray analysis of stably transfected monoclonal HEK293A cell lines expressing mutant and WT MEF2B-V5. Centred and scaled expression values in mutant MEF2B-V5, WT MEF2B-V5 and untransfected cells are indicated for genes that are differentially expressed in (**a**) K4E versus WT MEF2B-V5 cells, (**b**) Y69H versus WT MEF2B-V5 cells or (**c**) D83V versus WT MEF2B-V5 cells (B–H adjusted eBayes *P* values <0.05). (**d**) Centred and scaled expression values in mutant MEF2B-V5, WT MEF2B-V5 and untransfected cells are indicated for the 1,141 genes that were identified as candidate direct targets of WT MEF2B. For **a**–**d**, red indicates lower expression and yellow indicates higher expression. (**e**) MEF2B-V5 protein abundance was similar or greater in mutant compared with WT MEF2B-V5 cell lines, except for the Y69H MEF2B-V5 line. MEF2B-V5 was detected using V5 antibody. Plotted is the mean fold change compared with the WT line. Error bars represent the s.e.m. of three biological replicates.

We next investigated how the activity of MEF2B is altered by the *MEF2B* hotspot mutations identified in lymphoma, K4E, Y69H and D83V. All three of these mutations decreased the half-life of MEF2B (Supplementary Fig. 14) but permitted nuclear localization of MEF2B (Supplementary Fig. 15), indicating that they may not completely abrogate MEF2B's transcriptional activity. To explore effects of mutations on gene expression, we analysed microarray data from HEK293A cells stably expressing mutant or WT MEF2B-V5. We identified 975 DEGs in K4E versus WT MEF2B-V5 cells, 3,305 DEGs in Y69H versus WT MEF2B-V5 cells and 3,369 DEGs in D83V versus WT MEF2B-V5 cells (B–H adjusted eBayes *P* values <0.05, Supplementary Data 8–10). Depending on the mutant considered, 48–71% of the DEGs in mutant versus WT MEF2B-V5 cells were also DEGs in untransfected versus WT MEF2B-V5 cells (Supplementary Fig. 16). This indicated that mutant MEF2B had altered transcriptional activity at target genes of WT MEF2B. The expression of most target genes was altered further away from its levels in untransfected cells by the expression of WT MEF2B-V5 than by the expression of mutant MEF2B-V5 (Fig. 3a–c; Supplementary Fig. 16), consistent with the hypothesis that *MEF2B* mutations reduce MEF2B's capacity to regulate transcription. Specifically, mutant MEF2B appeared to have a reduced capacity to activate direct target gene expression compared with WT MEF2B, as expression of candidate direct target genes tended to be lower in cells with mutant than WT MEF2B-V5 (Fig. 3d).

The simplest explanation for how K4E, Y69H and D83V mutations may promote lymphoma development is that all three mutations do so through dysregulation of the same target genes. Thus, we identified 361 candidate MEF2B target genes that were DEGs in the K4E versus WT MEF2B-V5, Y69H versus WT MEF2B-V5 and D83V versus WT MEF2B-V5 comparisons. The expression of all 361 genes was altered further away from their levels in untransfected cells by the expression of WT MEF2B-V5 than by the expression of mutant MEF2B-V5 (Supplementary Fig. 16; Supplementary Data 11). These data are consistent with the notion that all three mutations decrease MEF2B's transcriptional activity. Interestingly, the tumour suppressor *TGFB1* was among the 361 common DEGs and was identified using IPA as a transcriptional regulator whose known target genes overlapped significantly with the 361 common DEGs (Fisher's exact test *P* value 0.002, activation z-score −3.4). Thus, decreased TGFβ signalling may play a central role in mediating effects of *MEF2B* mutation.

### Validation of decreases in transcriptional activity

Given that the abundance of K4E and D83V MEF2B-V5 was equal to or greater than that of WT MEF2B-V5 in the cell lines that were used for microarrays (Fig. 3e; Supplementary Fig. 17), the decrease in MEF2B transcriptional activity in K4E and D83V cells was not due to differences in MEF2B-V5 abundance. As Y69H MEF2B-V5 was less abundant than WT MEF2B-V5 in the cell lines that were used for microarrays (Fig. 3e), we generated an additional Y69H expressing line (‘Y69H E3') with greater MEF2B-V5 expression than WT MEF2B-V5 cells (Supplementary Fig. 18a). We then used qRT–PCR to assess whether the 27 genes whose differential expression had been validated for WT MEF2B-V5 versus empty vector cells were also differentially expressed in Y69H E3 versus WT MEF2B-V5 cells. There was a strong correlation between fold changes in expression in the microarray and qRT–PCR data, supporting the notion that Y69H decreases MEF2B transcriptional activity (Spearman correlation 0.73; Supplementary Fig. 18b,c). DEGs were also verified using RNA-seq and qRT–PCR on the cell lines that were used for microarrays. Gene expression changes in qRT–PCR and RNA-seq data correlated well with those in microarray data (Spearman correlations ⩾0.68; Supplementary Figs 18 and 19; Supplementary Data 12–14).

We expected that if K4E, Y69H and D83V mutations decreased MEF2B transcriptional activity, then K4E, Y69H and D83V mutations would have effects similar to those of mutations known to reduce the transcriptional activity of MEF2 proteins. Such mutations include R3T and R24L, which in MEF2A and MEF2C resulted in dominant negative activity^18^,^32^. MEF2B target gene expression tended to show the same direction of change in R3T or R24L versus WT MEF2B-V5 cells as in K4E, Y69H or D83V versus WT MEF2B-V5 cells (that is, decreased expression; Supplementary Fig. 20). These data support the notion that K4E, Y69H and D83V mutations, such as R3T and R24L mutations, reduce MEF2B's capacity to activate transcription.

**Figure 4:**
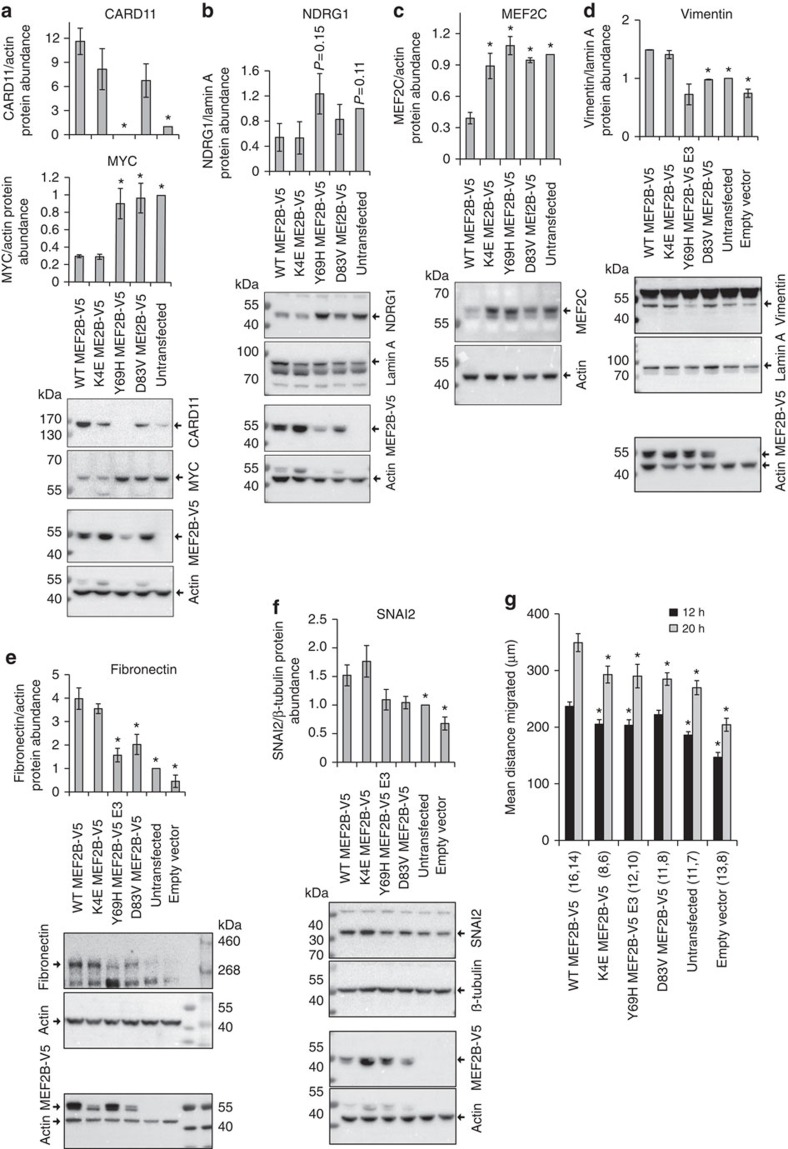
*MEF2B* mutations alter the abundance of protein from MEF2B target genes and decrease cell migration. (**a**–**f**) CARD11, MYC, NDRG1, MEF2C, vimentin, fibronectin and SNAI2 protein abundance in cells expressing WT or mutant MEF2B-V5. The MEF2B-V5 bands shown in **a** were detected on the same western blot as was used for MYC and CARD11 detection. (**b**,**c**) The same lysates were used for NDRG1 and MEF2C western blots as for the MEF2B-V5 western blots shown in **b**. (**d**–**f**) The same lysates were used for the SNAI2, vimentin and fibronectin western blots as for the MEF2B-V5 western blots shown below them. Mean relative abundance was calculated compared with untransfected cells using densitometry on three biological replicates of western blots. Representative western blots are shown. MEF2B-V5 was detected using V5 antibody. (**g**) Movement into the scratched area of a confluent monolayer is increased more by WT than mutant MEF2B-V5 expression. Scratches were assessed in at least two biological replicates (K4E: two biological replicates; Y69H and D83V: three biological replicates; WT, untransfected and empty vector: four biological replicates). The total number of scratches assessed for each sample is shown in parentheses for 12 and 20 h time points, respectively. For all panels, *P<0.05 in comparison with WT MEF2B-V5 expressing cells (Student's two-tailed *t*-test, unpaired). Error bars represent the s.e.m.

We next sought to validate our findings by assessing whether differences in messenger RNA (mRNA) abundance corresponded with differences in protein abundance. We assessed abundance of the seven proteins that we demonstrated were affected by expression of WT MEF2B-V5 (that is, MYC, CARD11, NDRG1, MEF2C, vimentin, fibronectin and SNAI2). Changes in protein abundance were consistent with the changes in mRNA expression (Fig. 4a–f; Supplementary Figs 21 and 22). Consistent with predictions made using IPA (Supplementary Fig. 23; Supplementary Table 5), K4E, Y69H and D83V MEF2B-V5 cells all showed less cell migration in scratch assays than WT MEF2B-V5 cells (Fig. 4g). Faster scratch closure was likely due to decreased cell migration, not decreased proliferation, as no differences in proliferation were detected between mutant and WT MEF2B-V5 cells (Supplementary Fig. 5).

### K4E and D83V *MEF2B* mutations decrease MEF2B DNA binding

**Figure 5:**
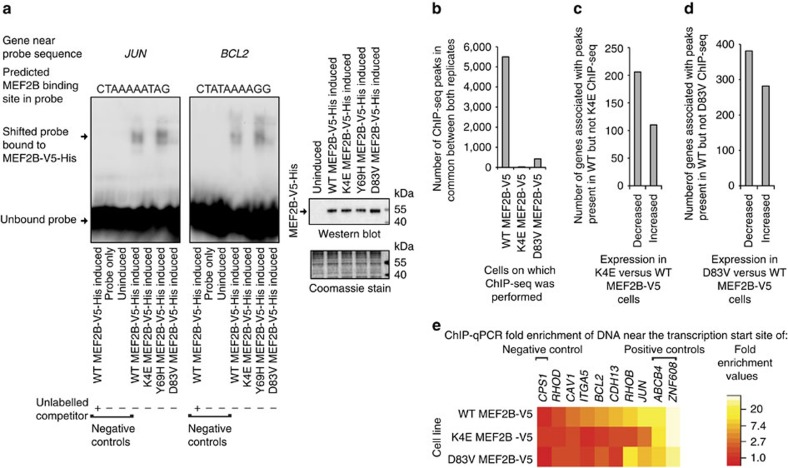
K4E and D83V MEF2B binds HEK293A cell DNA at fewer sites than WT MEF2B. (**a**) Gel-shift assays indicate that K4E and D83V mutations decrease MEF2B DNA binding. Probes contained 35–37 bp of genomic sequence located near the centre of a MEF2B-V5 ChIP-seq peak within 5 kb of the transcription start site of the indicated gene. The unlabelled competitor consisted of the same sequence as the labelled probe. Lysates were from *E. coli* with or without induction of *MEF2B-V5-His* expression. (**b**) More peaks were identified in V5 ChIP-seq on WT MEF2B-V5 cells than in V5 ChIP-seq on K4E or D83V MEF2B-V5 cells. Only peaks identified in both replicates of the ChIP-seq on a cell type were counted. (**c**,**d**) Regions with peaks in both replicates of V5 ChIP-seq on WT MEF2B-V5 cells but neither replicate of V5 ChIP-seq on (**c**) K4E nor (**d**) D83V MEF2B-V5 cells were associated with genes using GREAT^27^. Shown are the numbers of the associated genes that were differentially expressed in mutant versus WT MEF2B-V5 cells (B–H adjusted eBayes *P* values <0.05). For **b**–**d**, ChIP-seq peaks were identified over input control DNA at a MACS2 (ref. 29) false discovery rate of 0.05. (**e**) V5 ChIP-qPCR on WT MEF2B-V5 cells tended to produce greater fold enrichments than V5 ChIP-qPCR on K4E and D83V MEF2B-V5 cells. Shown are mean fold enrichments of three biological replicates. Fold enrichment was calculated compared with an intergenic region not expected to interact with MEF2B, then normalized to ChIP–qPCR using normal immunoglobulin.

We next investigated whether target gene expression differences in mutant versus WT MEF2B-V5 lines could be explained by differences in MEF2B DNA binding. In gel-shift assays, Y69H and WT MEF2B-V5-His shifted similar amounts of probe, whereas D83V and K4E MEF2B-V5-His shifted little and no detectable probe, respectively (Fig. 5a; Supplementary Fig. 9). These data indicated that D83V and K4E mutations reduced direct MEF2B DNA binding, whereas the Y69H mutation had no apparent effect on direct MEF2B DNA binding.

We then used V5 ChIP-seq to investigate whether genome-wide patterns of DNA binding differed between K4E, D83V and WT MEF2B. Consistent with the notion that K4E and D83V mutations reduced DNA binding, the number of peaks identified in both replicates of K4E and D83V ChIP-seq was only 0.6% and 7.7%, respectively, of the number of peaks identified in both replicates of WT ChIP-seq (Fig. 5b). Regions where peaks were lost in K4E or D83V ChIP-seq were highly enriched for MEF2A and MEF2C motifs (Supplementary Table 6), supporting that K4E and D83V mutations disrupt interactions with MEF2 motifs.

We further investigated the 36 and 424 peaks that were identified in both replicates of K4E and D83V ChIP-seq, respectively. Of these peak regions, 97% and 91%, respectively, also had peaks in both replicates of WT ChIP-seq, indicating that K4E and D83V DNA binding tends to remain restricted to sites that WT MEF2B can bind. The most enriched motifs in K4E and D83V ChIP-seq peak regions were those of MEF2A and MEF2C (Supplementary Table 7), indicating that K4E and D83V MEF2B retain specificity for binding MEF2 motifs. Although K4E MEF2B-V5 appeared not to bind MEF2 motifs in gel-shift assays, heterodimers of WT and K4E MEF2 proteins may retain some capacity to bind MEF2 sequences.

### Integrative analysis of ChIP-seq and gene expression data

We then assessed whether differences in DNA binding corresponded to differences in gene expression. As fewer peaks were identified in mutant than WT MEF2B-V5 ChIP-seq, we expected that many of the genes associated with WT ChIP-seq peaks would not have associated peaks in mutant ChIP-seq. Indeed, among the genes associated with peaks in both replicates of WT ChIP-seq, 94.3% were not associated with peaks in either replicate of K4E ChIP-seq and 34.4% were not associated with peaks in either replicate of D83V ChIP-seq. Among these genes associated with WT but not mutant peaks, more genes had decreased than increased expression in mutant versus WT MEF2B-V5 cells (Fig. 5c,d). These data are consistent with the notion that the decreased interaction of K4E and D83V MEF2B-V5 with DNA tends to decrease target gene expression. Also, consistent with this conclusion, regions with peaks in both replicates of WT ChIP-seq but neither replicate of mutant ChIP-seq tended to be closer to genes with decreased expression in mutant versus WT MEF2B-V5 cells than to other genes (Supplementary Fig. 24).

We then used ChIP–qPCR to verify K4E and D83V ChIP-seq data for the seven regions at which WT MEF2B-V5 binding had been validated by ChIP–qPCR (that is, regions near *BCL2*, *JUN*, *RHOB*, *CDH13*, *ITGA5*, *CAV1* and *RHOD*). None of these regions had peaks in either replicate of K4E ChIP-seq, and only the region near *RHOB* had a peak in D83V ChIP-seq. Consistent with this ChIP-seq data, five out of the seven regions showed a decrease in enrichment of at least 40% in both K4E and D83V ChIP–qPCR compared with WT ChIP–qPCR (Fig. 5e; Supplementary Fig. 25). The genes near those five regions, *BCL2*, *JUN*, *CDH13*, *ITGA5* and *CAV1*, had decreased expression in K4E or D83V versus WT MEF2B-V5 (eBayes *P* values <0.006), perhaps because of decreased MEF2B interaction with their regulatory regions.

### MEF2 family genes are expressed in DLBCL cells

As the *MEF2B* mutations we characterized were identified in DLBCL^14^,^15^,^16^, we next investigated MEF2B's role in DLBCL cells. We first sought to determine whether *MEF2* genes other than *MEF2B* are expressed in DLBCL cells. Not only were *MEF2A*, *MEF2C* and *MEF2D* mRNAs detected in DLBCL patient samples using RNA-seq^14^, they were more abundant than *MEF2B* mRNA (Supplementary Fig. 26a). MEF2C protein was detected on western blots of DLBCL cell lines (Supplementary Fig. 26b). We also used RNA-seq data from DLBCL patient samples^14^ to determine the predominant *MEF2B* isoform. *MEF2B* transcripts (91.7%) were isoform A, which was the isoform we selected for our studies (Supplementary Fig. 26c,d). Next, we used mass spectrometry to confirm that both mutant and WT MEF2B protein were present in a DLBCL cell line with an endogenous D83V mutation (Supplementary Fig. 26e). These data are consistent with the notion *MEF2B* mutations can promote lymphoma development even when WT MEF2B remains present.

### *MEF2B* mutations decrease *BCL6* expression in DLBCL cells

**Figure 6:**
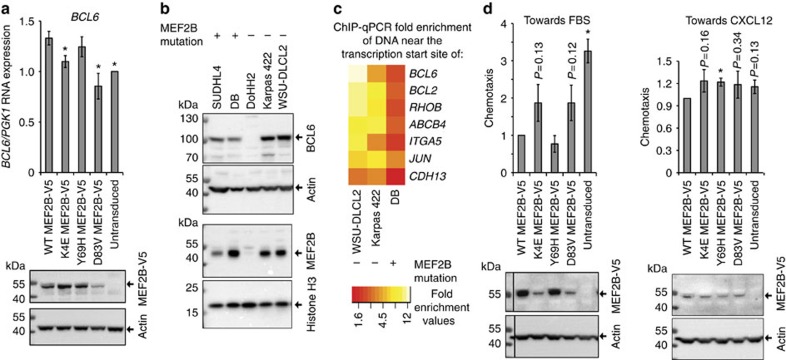
MEF2B-V5 tends to promote BCL6 expression and inhibit chemotaxis in DLBCL. (**a**) *BCL6* mRNA expression in DoHH2 DLBCL cells expressing mutant or WT MEF2B-V5. The mean fold change in mRNA expression compared with untransduced cells was determined using qRT–PCR data normalized to *PGK1* expression. Values are the mean of six biological replicates. (**b**) BCL6 protein expression in DLBCL cell lines with and without endogenous *MEF2B* mutations. (**c**) ChIP–qPCR on DLBCL cells with no *MEF2B* mutations (that is, Karpas 422 and WSU-DLCL2) tended to produce greater fold enrichments than ChIP–qPCR on DLBCL cells with an endogenous D83V *MEF2B* mutation (DB). Shown are the mean fold enrichments of three biological replicates. Fold enrichment was calculated compared to ChIP–qPCR using normal immunoglobulin. (**d**) Expression of WT MEF2B-V5 tended to decrease DoHH2 cell chemotaxis towards fetal bovine serum (FBS) and CXCL12. Shown is the mean fold change in the proportion of cells crossing a Transwell membrane, compared with WT MEF2B-V5-expressing cells. Values are the mean of four (FBS) or five (CXCL12) biological replicates. For all panels, western blots show protein expression in cells at the time of the experiment. MEF2B-V5 was detected using V5 antibody. Black lines through western blots indicate removed lanes. Full blots are shown in Supplementary Fig. 27. *P<0.05 in comparison with WT MEF2B-V5 cells (Student's two-tailed *t*-test, unpaired). Error bars indicate the s.e.m.

We then aimed to determine whether *MEF2B* mutations reduced MEF2B's capacity to activate transcription in DLBCL cells. We transduced WT and mutant MEF2B-V5 into the DoHH2 DLBCL cell line and assessed expression of *BCL6*, a lymphoma oncogene regulated by MEF2B^2^. Consistent with the notion that MEF2B promotes *BCL6* expression, *BCL6* mRNA expression tended to be greater in WT MEF2B-V5 DoHH2 cells than in untransduced cells (Fig. 6a; Supplementary Fig. 27a). Consistent with our finding that *MEF2B* mutations decrease the capacity of MEF2B to activate target gene expression in HEK293A cells, *BCL6* mRNA expression tended to be lower in cells expressing K4E and D83V MEF2B-V5 than in cells expressing WT MEF2B-V5 (Fig. 6a).

To further explore possible effects of *MEF2B* mutation on *BCL6* expression, we assessed the abundance of BCL6 protein across a panel of germinal centre B-cell (GCB) DLBCL cell lines with WT *BCL6* alleles^2^,^14^. BCL6 protein levels were the lowest in the cell line with the lowest MEF2B abundance (Fig. 6b; Supplementary Fig. 27b), consistent with the notion that MEF2B activates *BCL6* expression. DLBCL cell lines with endogenous *MEF2B* mutations (DB and SUDHL4) had lower BCL6 protein levels than those with WT MEF2B (WSU-DLCL2 and Karpas 422; Fig. 6b), consistent with our findings that *MEF2B* mutations decrease transcriptional activation.

### Promoter DNA bound by endogenous MEF2B in DLBCL cells

We next used a MEF2B antibody in ChIP–qPCR to identify genomic loci bound by endogenous MEF2B in DLBCL cells. We assessed seven regions validated using ChIP–qPCR in HEK293A cells. Regions near *BCL6*, *BCL2*, *RHOB, ABCB4, ITGA5* and *JUN* had enrichments that were greater than fourfold and were statistically significant in at least one of the DLBCL cell lines compared with an IgG control (Fig. 6c; Supplementary Fig. 28). These genes may thus be MEF2B direct target genes in DLBCL cells. The ChIP–qPCR data are also consistent with our findings that the D83V mutation decreased MEF2B DNA binding: ChIP–qPCR fold enrichments tended to be lower for cells with an endogenous D83V *MEF2B* mutation (that is, the DB cell line) than for the cell lines without *MEF2B* mutations (Fig. 6c; Supplementary Fig. 28).

### Isoform B MEF2B has decreased transcriptional activity

If the K4E, Y69H and D83V mutations promote lymphoma development by decreasing the capacity of MEF2B to activate transcription, the most parsimonious explanation for how other *MEF2B* mutations contribute to lymphoma development would be that they also decrease MEF2B's capacity to activate transcription. Some MEF2B mutations identified in lymphoma (that is, P256, P267 and L269 frameshift mutations^2^,^14^,^16^) were predicted to cause proteins similar to isoform B MEF2B to be produced from isoform A *MEF2B* transcripts^2^ (see Supplementary Fig. 1a in ref. 2). Thus, we hypothesized that isoform B MEF2B has decreased transcriptional activity compared with isoform A MEF2B. This hypothesis was supported by evidence that isoform A-specific regions were required for efficient transcriptional activation: deletions from the C terminus to D272 or Y223 in the mouse homologue of isoform A MEF2B resulted in reduced transcriptional activity^18^. Isoform B differs from isoform A in all amino acids C terminal to P256 as a result of a frameshift at a splice junction. Supporting the hypothesis that isoform B MEF2B has decreased transcriptional activity compared with isoform A MEF2B, expression of isoform B MEF2B-V5 altered target gene expression to a lesser extent than expression of isoform A MEF2B-V5 in HEK293A cells (Supplementary Fig. 29).

### MEF2B activity inhibits DLBCL chemotaxis

We next explored phenotypes that might be affected by *MEF2B* mutations in DLBCL cells. We first used RNA-seq data^14^ to identify 489 DEGs in 13 *MEF2B* mutant versus 40 WT DLBCL patient samples (B–H adjusted DEseq^33^ *P* values <0.1; Supplementary Data 15). The most enriched annotation category was ‘cellular movement' (B–H adjusted right-tailed Fisher exact test *P* value 1 × 10^−3^; Supplementary Fig. 30a). ‘Cellular movement' remained enriched (B–H adjusted right-tailed Fisher exact test *P* value 0.028) when DEGs were identified at B–H adjusted DEseq^33^ *P* values<0.05, although confidence in the prediction of change direction was reduced (IPA z-score reduced from 2.5 to 0.68).

In contrast to our finding that *MEF2B* mutations decreased HEK293A cell movement, *MEF2B* mutations were predicted to increase DLBCL cell movement (Supplementary Table 8). This difference may be because DLBCL cell movement is regulated by a different set of MEF2B target genes than HEK293A cell movement. Indeed, a greater proportion of the cellular movement annotation groups enriched in the DLBCL DEGs compared with those enriched in HEK293A DEGs were specific to blood or immune system cells (DLBCL: 16/27 annotation groups; HEK293A: 2/44 annotation groups) or related to chemotaxis (DLBCL: 14/27 annotation groups; HEK293A: 4/44 annotation groups). Moreover, of the 30 DEGs in MEF2B mutant versus WT DLBCL patient samples (B–H adjusted DEseq^33^ *P* values <0.05) that were also DEGs in WT MEF2B-V5 versus untransfected HEK293A cells, only four had annotated functions related to cellular movement (*EPHA7*, *ATOH1*, *DPYSL5* and *NTF3*; Supplementary Table 9). Thus, opposite effects of MEF2B on cell movement in HEK293A and DLBCL cells might arise due to cell-specific differences in the genes mediating cell migration.

Supporting the hypothesis that increased cell movement may contribute to DLBCL development, cellular movement was also the most enriched annotation category (B–H adjusted right-tailed Fisher exact test *P* value 2 × 10^−44^; Supplementary Fig. 30b) in the 5,042 genes differentially expressed (B–H adjusted DEseq^33^ *P* values <0.05; Supplementary Data 16) between 53 GCB DLBCL samples and 13 normal centroblast samples. Moreover, this analysis predicted that DLBCL cells would be more migratory than centroblasts (Supplementary Data 17).

To validate these predictions, we assessed chemotaxis of DoHH2 cells expressing mutant or WT MEF2B-V5 towards fetal bovine serum (FBS). Chemotaxis towards FBS tended to be lower in cell lines with greater MEF2B expression (Fig. 6d; Supplementary Fig. 27c), consistent with the notion that MEF2B activity inhibits chemotaxis. We next assessed chemotaxis towards CXCL12, a chemokine that attracts germinal centre (GC) B-cells towards the dark zone of germinal centres^34^. Despite having similar MEF2B-V5 expression, cells expressing K4E, Y69H or D83V MEF2B-V5 tended to show greater chemotaxis towards CXCL12 than cells expressing WT MEF2B-V5 (Fig. 6d; Supplementary Fig. 27c). These data are consistent with the notion that *MEF2B* mutations reduce inhibition of DLBCL chemotaxis, as was predicted from our gene expression analysis. Untransduced cells also tended to have increased chemotaxis compared with WT cells, indicating that decreased MEF2B activity tends to reduce inhibition of chemotaxis towards CXCL12.

## Discussion

We provide the first comprehensive identification of both MEF2B binding sites and genes differentially expressed in response to *MEF2B* mutation. MEF2B direct target genes include *RHOB*, *RHOD*, *CDH13*, *ITGA5* and *CAV1*, and MEF2B indirect target genes include *MYC*, *TGFB1, CARD11*, *MEF2C*, *NDRG1* and *FN1*. None of these genes have previously been identified as MEF2B targets. Moreover, we show for the first time that MEF2B increases cell migration and the expression of genes involved in EMT, perhaps by decreasing TGFβ1 signalling^23^, decreasing *SNAI2* expression^23^ and increasing *NDRG1* expression^35^. MEF2B is amplified in several types of carcinoma^11^,^12^ perhaps because MEF2B promotes EMT. Promotion of EMT by MEF2B would also be consistent with evidence that all human MEF2 proteins blocked mesenchymal to epithelial transition^36^ and with evidence that MEF2A MEF2C and MEF2D promoted EMT of hepatocellular carcinoma cells^37^.

K4E and D83V MEF2B showed decreased DNA binding compared with WT MEF2B. Effects of these two mutations on DNA binding were not surprising, as K4 is located at the DNA-binding interface and deletion of residues 77–80 in the paralogue MEF2C reduced MEF2C DNA binding^18^. The decreased capacity of mutant MEF2B to bind DNA likely contributes to its decreased ability to activate transcription. Y69H also decreased the capacity of MEF2B to activate target gene expression but did not affect direct DNA binding. As Y69H is located at the interface of MEF2 proteins where the co-activator p300 binds^38^, Y69H may decrease transcriptional activation by decreasing co-activator recruitment. Indeed, possible effects of Y69H and D83V mutation on activities other than DNA binding, such as co-activator recruitment, may explain why the Y69H and D83V mutations affected the expression of more genes than the K4E mutation, despite having a less severe effect on DNA binding than the K4E mutation. K4E is located at the interface of MEF2B and DNA and is thus unlikely to affect co-factor interactions.

As the *MEF2B* mutations identified in DLBCL appear heterozygous^14^, it is likely that either MEF2B is haploinsufficient, or that mutant MEF2B acts as a dominant negative. Supporting the latter notion, no nonsense or frameshift mutations affecting the predicted dimerization domains of MEF2B have been reported. Consistent with the idea that decreased MEF2B activity promotes DLBCL development, two cases of homozygous MEF2B deletion have been identified in DLBCL^11^,^12^.

Similar to *MEF2B* mutations, recurrent mutations affecting the chromatin-modifying enzymes EZH2 and KMT2D (also called MLL2) in DLBCL are also thought to reduce target gene expression^14^,^39^. As EZH2, KMT2D and MEF2 proteins are thought to cooperatively regulate common target genes in skeletal muscle^40^, the effects of *MEF2B* mutations may converge with those of *KMT2D* and *EZH2* in DLBCL.

Our evidence that *MEF2B* mutations decrease MEF2B transcriptional activity and *BCL6* expression is inconsistent with a report that *MEF2B* mutations increased *BCL6* expression^2^. However, we note that our work included a comparison of mutant and WT MEF2B transduced into the same DLBCL cell line, assessed expression of multiple target genes and explained how the K4E mutation alters the function of MEF2B. Our study revealed gene expression changes in mutant versus WT cells that are expected to promote cancer development, notably increased expression of the *MYC* oncogene^41^ and decreased activity of the *TGFB1* tumour suppressor^42^.

We also report for the first time that *MEF2B* mutations reduce inhibition of DLBCL cell chemotaxis. Reduced inhibition of chemotaxis was associated with DLBCL development in S1P_2_-deficient mice^43^ and Gα13-deficient mice^44^. Moreover, the genes encoding Gα13 and S1P_2_ are recurrently mutated in DLBCL^14^,^45^. Thus, reduced inhibition of chemotaxis as a result of *MEF2B* mutations may promote DLBCL development. Our study provides a unique resource for exploring the role of MEF2B in cell biology. We map for the first time the MEF2B ‘regulome', demonstrating connections between a relatively understudied transcription factor and multiple oncogenic drivers.

## Methods

### Cell lines and treatments

Information on the sources, authentication and *Mycoplasma* testing of cell lines is provided in Supplementary Table 10. Cell lines were mycoplasma tested using the e-Myco mycoplasma PCR detection kit (iNtRON). HEK293A cell lines were used because they are an experimentally tractable and well-characterized model system^46^. HEK293A cells were grown in DMEM (Gibco) with 10% FBS (Gibco). DLBCL lines were grown in RPMI (Gibco) with 10% FBS. All DLBCL cell lines were of the germinal centre B-cell subtype^14^,^47^. The *MEF2B* mutation status of the DLBCL cell lines was reported previously^2^,^14^ Transfected HEK293A were grown in 100 μg ml^−1^ G418 (Invitrogen). Transduced DoHH2 were grown in 7.5 μg ml^−1^ Blasticidin S (Invitrogen). Antibiotics were removed 24–48 h before harvesting cells. Cyclohexamide (Abcam) was used at 75 μg ml^−1^. Cells for microarray and RNA-seq analysis were treated for 6 h with 1.07 μl ml^−1^ of dimethyl sulfoxide (Fisher) immediately before RNA was collected, as a solvent-only control for drug treated cells (data from drug-treated cells was not presented).

### Production of stably transduced cell lines

Isoform A (GeneCopoeia GC-Z7031-CF) and isoform B (GeneCopoeia GC-F0247-CF) MEF2B were obtained in pDONR vectors. Experiments used isoform A unless indicated otherwise. Mutations were introduced into isoform A MEF2B using the QuikChange II site-directed mutagenesis kit (Agilent). MEF2B was transferred into vectors containing C-terminal V5 tags, pDEST40 and pLenti6.2 (Invitrogen), using the Gateway LR Clonase Enzyme Mix (Invitrogen). Plasmids were sequenced using an ABI Prism 3100 Genetic Analyser (Applied Biosystems) to confirm the absence of other *MEF2B* mutations. pDEST40 constructs and an empty pcDNA3 vector (Invitrogen) were transfected into HEK293A cells using TurboFect (Thermo Scientific). Stably transfected cells were selected over three weeks using 200 μg ml^−1^ G418 (Invitrogen). WT-, K4E-, Y69H-, D83V- and R24L MEF2B-V5-expressing HEK293A cell lines were monoclonal, isolated by dilution of transfected cells to single cells per well before G418 selection. R3T MEF2B-V5-expressing cells were oligoclonal, and WT isoform B MEF2B-V5-expressing cells were polyclonal, as no monoclonal lines with suitable MEF2B-V5 expression were isolated from these transfections.

To package pLenti6.2 constructs into replication-defective lentiviruses, HEK293T cells were co-transfected with pLenti6.2, CMVDeltaR8.91 and pMD2 VSV-G envelope constructs (gifts from Eric Young, BC Cancer Research Centre) using TransIT-LTI transfection reagent (Mirus Bio). Virus particles collected after 48 and 72 h were passed through a 0.45-μm filter, concentrated and applied overnight with Polybrene (Sigma) to DoHH2 cells in 10% FBS RPMI. A polyclonal population of stably transduced cells was selected with 7.5 μg ml^−1^ Blasticidin S (Invitrogen) over 3 weeks. As no cells stably transfected with empty pLenti6.2 vector could be isolated, untransduced DoHH2 cells were used for comparison with MEF2B-V5 DoHH2 cells. The DoHH2 cell line was used for transductions because it had very low endogenous *MEF2B* RNA expression (Fig. 6b). Thus, transduced MEF2B-V5 was likely to comprise a larger fraction of the total MEF2B protein in DoHH2 cells than it would in other DLBCL cell lines.

### Expression microarray analysis

RNA was isolated using the RNeasy plus kit (Qiagen) from three biological replicates of each cell line. RNA labelling and hybridization to GeneChip Human Exon 1.0 ST arrays (Affymetrix) were performed by the McGill University and Genome Quebec Innovation Centre. Probe signal values were normalized using Robust Multi-array Average (RMA) analysis^48^ in the Affymetrix Expression Console, with gene level summarization of core probeset data. The eBayes algorithm of the Linear Models for Microarray Data (LIMMA) Bioconductor package^49^ with B–H multiple testing correction was used to identify DEGs from microarray data. LIMMA^49^ was used for identification of DEGs as it has shown greater power and lower false positive rates compared with other algorithms when working with sample sizes of less than five^50^. Although LIMMA is a parametric technique and techniques for validating the assumption of normality are not appropriate for the small sample sizes used here, LIMMA is robust to considerable deviation from normal distributions^50^ and has been widely used on gene expression data sets. The groups compared showed similar variance in gene expression values, consistent with assumptions of LIMMA (Supplementary Fig. 31a). The data is accessible through GEO dataset GSE67458.

Annotation group enrichment and upstream regulator analyses were performed using Ingenuity Integrative Pathway Analysis of Complex omic's Data (IPA) version 14855783 (Qiagen). Analyses considered only molecules and relationships where the species was human and the confidence was either experimentally observed or highly predicted. IPA Upstream Regulator Analysis *P* values indicate the probability that the overlap between the user-provided DEG list and the known target gene set of a potential upstream regulator is due to chance. For both annotation group enrichment and upstream regulator analysis, z-scores indicate the confidence in the predicted direction of activity change. As recommended by IPA, only absolute z-scores greater than two were considered significant. Gene Set Enrichment Analysis was performed as described, using default parameters^21^,^22^.

### Verification and validation of expression microarray data

All primer sequences are provided in Supplementary Table 11. For all qRT–PCR primers, neighbouring exons that were both highly differentially expressed between cell types were selected as primer sites. Such exons were identified using LIMMA^49^ on data RMA^48^ normalized with exon level summarization. Where exons were similarly differentially expressed, those separated by the largest intron were selected as primer sites. qRT–PCR was performed using the Power SYBR Green RNA-to-CT 1-Step Kit (Life Technologies) and a 7900 HT Sequence Detection System with SDS 2.2 software (Applied Biosystems). Gene expression was normalized to *PGK1* expression because *PGK1* was not differentially expressed in the microarray data and was previously identified as a reference gene for gene expression studies^51^,^52^,^53^. A gene was considered to validate for a given comparison if it met the following two criteria: (i) the gene was differentially expressed in both microarray (B–H adjusted eBayes *P* value <0.05) and qRT–PCR data (Student's *t*-test *P* value <0.05), and (ii) the gene had the same direction of expression change in both microarray and qRT–PCR data.

### mRNA library construction

RNA was isolated using the RNeasy plus kit (Qiagen) from one replicate of each cell line. Plate-based libraries were prepared following the BC Cancer Agency's Michael Smith Genome Sciences Centre (BCGSC) strand specific paired-end protocol on a Biomek FX robot (Beckman-Coulter) with Ampure XP SPRI beads (Beckman-Coulter). First-strand complementary DNA (cDNA) was synthesized using the Superscript cDNA Synthesis kit (Life Technologies) with random hexamer primers and 1 μg μl^−1^ Actinomycin D. The second strand cDNA was synthesized following the Superscript cDNA Synthesis protocol, but substituting dTTP for dUTP. The cDNA was fragmented in an E210 sonicator (Covaris) for 55 s, using a duty cycle of 20% and intensity of 5. Purified cDNA was subjected to end repair and phosphorylation by T4 DNA polymerase (NEB), Klenow DNA polymerase (NEB) and T4 polynucleotide kinase (NEB) in a single reaction, followed by 3′ A-tailing by Klenow fragment (3′–5′ exo minus, NEB). Products were ligated to Illumina PE adaptors. The first strand was then digested using uracil-*N*-glycosylase (Life Technologies), thus achieving strand specificity. After purification, products were PCR-amplified using Phusion DNA Polymerase (Thermo Fisher Scientific) and Illumina's PE primer set, with cycle conditions of 98 °C for 30 s followed by 10–15 cycles of 98 °C for 10 s, 65 °C for 30 s and 72 °C for 30 s, and then 72 °C for 5 min. Purified PCR products were analysed using a LabChip GX (PerkinElmer). PCR products with a desired size range were purified using a 96-channel size selection robot developed at the BCGSC. The DNA quality was assessed using an Agilent DNA 1000 series II assay. DNA was quantified using a Quant-iT dsDNA HS Assay Kit (Invitrogen) and diluted to 8 nM.

### Sequencing and alignment of ChIP and RNA sequencing data

ChIP and RNA libraries were sequenced on the Illumina HiSeq 2000/2500 platform using v3 chemistry and HiSeq Control Software version 2.0.10. ChIP libraries were sequenced 8 per lane, ChIP input control DNA was sequenced 14 per lane and RNA libraries were sequenced 2 per lane. All lanes were 75-bp paired-end sequencing. Sequencing was performed at the BC Cancer Agency's Michael Smith Genome Sciences Centre and passed all quality control thresholds (quality control data shown in Supplementary Tables 3 and 12). The GEO accession number for all sequencing data is GSE67458.

Sequencing data were aligned to the GRCh37-lite genome-plus-junctions reference (http://www.bcgsc.ca/downloads/genomes/9606/hg19/1000genomes/bwa_ind/genome) using BWA (version 0.5.7)^54^ with default parameters. Reads failing the Illumina chastity filter were flagged with a custom script and duplicated reads were flagged with Picard Tools (version 1.31, http://broadinstitute.github.io/picard/). Unmapped reads, optical duplicates and PCR duplicates were removed. Similar numbers of mapped reads were produced for all samples within ChIP-seq and RNA-seq data sets (Supplementary Fig. 32). WIG files for viewing in UCSC genome browser (hg19) were produced using BAM2WIG (http://www.epigenomes.ca/tools.html). The WIG files were used in an in-house pipeline used previously^14^ to calculate RPKM values for each gene. Briefly, the total sequence base coverage across each exon was calculated from the WIG files. This number was then divided by the read length to obtain the number of reads mapping to a gene. RPKM was then calculated by dividing the number of reads mapping to a gene by the length of the gene's collapsed exons in kilobases and then dividing by the total number of millions of mapped reads.

### Differential expression analysis of HEK293A RNA-seq data

Differential expression analysis of RNA-seq data used DEseq Release 2.13 (Bioconductor)^33^ on genes with at least one read in all samples. To enable analysis of data with only one replicate per group, ‘SharingMode' was set to ‘fit-only' and ‘method' was set to ‘blind'. Default settings were otherwise used. DEseq was used because it was recommended over other frequently used tools for cases where false positives are a concern^55^. Comparison of gene expression changes in RNA-seq and microarray data used Spearman correlation coefficients rather than Pearson correlation coefficients as Spearman coefficients are more robust to outliers and do not assume the data follows a normal distribution.

RNA-seq DEGs for IPA analysis were those with B–H adjusted DEseq^33^ *P* values <0.05. Fewer DEGs were identified using the HEK293A RNA-seq data than were identified using the microarray data likely because fewer samples were used of each cell type for RNA-seq than for microarrays (that is, one replicate for RNA-seq, three replicates for microarray). The smaller sample size would reduce statistical power to identity DEGs, reducing the likelihood of a DEGs being identified at a given confidence level.

### ChIP for sequencing

Eight 40–80% confluent 15-cm plates were grown of each cell type and treated with 1% formaldehyde (Sigma) for 10 min, before treatment with one-tenth volume of 1.25 μM glycine (Sigma) for 5 min. EDTA-free complete protease inhibitor cocktail (Roche) was added fresh to lysis buffer (50 mM Tris-HCl pH 8.0, 1% SDS, 10 mM EDTA) and IP buffer (10% Tris-HCl pH 8.0, 1% Triton X-100, 0.1% deoxycholate, 0.1% SDS, 90 mM NaCl, 2 mM EDTA). Cells were lysed on ice for 30 min in lysis buffer, passed through a 22-G needle, and centrifuged (5,000*g*, 10 min). The chromatin pellet was resuspended in 400 μl of lysis buffer and split into two equal halves for sonication (20 min, 30 s on, 30 s off, power level 6) in a Sonicator 3000 (Misonix). Insoluble cell debris and unfragmented chromatin were removed by centrifugation (13,000*g*, 12 min). An aliquot of chromatin was purified to allow determination of chromatin concentration and confirm that DNA fragments were present in the 200–500 bp size range. Protein G Dynabeads (Life Technologies) blocked with bovine serum albumin and salmon sperm DNA were used to pre-clear chromatin. To maximize the amount of DNA for library construction while still keeping samples within a replicate comparable to each other, 675 μg of chromatin were used for all replicate 1 samples and 1,460 μg of chromatin were used for all replicate 2 samples. Chromatin volumes were equalized using lysis buffer and one-fourth volume of IP buffer was added. Chromatin was incubated with 29 μl V5 mouse antibody (Invitrogen R960–25) or normal mouse IgG (Santa Cruz sc-2025) for 1 h at 4 °C before addition of 272 μl of blocked Dynabeads. After overnight incubation at 4 °C, samples were washed twice in low salt buffer (20 mM Tris-HCl pH 8.0, 0.1% SDS, 1% Triton X-100, 2 mM EDTA, 150 mM NaCl) and once in high salt buffer (same as low salt buffer except 500 mM NaCl). DNA was eluted in 100 mM sodium bicarbonate with 1% SDS at 68 °C for 2 h. Eluted DNA was purified by phenol chloroform extraction in Phase Lock Gel tubes (5 Prime) and ethanol precipitated with 40 μg glycogen (Roche). Input controls were produced by purifying 100 ng of chromatin from each sample in the same manner as the immunoprecipitated chromatin was purified.

### ChIP library construction

ChIPed DNA was size separated using 8% polyacrylamide gel electrophoresis (PAGE). The 200–500-bp DNA fractions were excised from the gel and were eluted from the gel slice overnight at 4 °C in elution buffer. Elution buffer consisted of a 5 to 1 ratio of LoTE buffer (3 mM Tris-HCl, pH 7.5, 0.2 mM EDTA) and 7.5 M ammonium acetate. DNA was purified using a Spin-X Filter Tube (Fisher Scientific, UK), and ethanol precipitated.

Library construction was carried out on the Bravo liquid handling platform using VWorks Automation Control Software (Agilent Automation). Samples were first subjected to end repair using T4 polynucleotide kinase (NEB), T4 DNA polymerase (NEB) and Klenow DNA polymerase (NEB) at room temperature for half an hour. DNA was purified using PEG-Sera Mag Speedbeads (Fisher) with 13.87% final polyethylene glycol (PEG) concentration. A-tailing was then performed using Klenow exo minus (NEB) at 37 °C for 30 min. Products were purified as before. Illumina short sequencing adaptors were ligated to A-tailed product using T4 DNA ligase (NEB) at room temperature overnight. To remove adaptor dimers and library fragments below 200 bp, products were purified twice using PEG-Sera Mag Speedbeads with 8.89% and 10.91% final PEG concentrations. Adaptor ligated libraries were PCR amplified and barcoded using custom indexing primers, Illumina PCR primer 1.0 and 0.5 U of Phusion Hot Start II (Fisher). The initial denaturation step at 98 °C for 30 s was followed by 13 cycles of 15 s at 98 °C, 30 s at 65 °C and 30 s at 72 °C, and a final step at 72 °C for 5 min. Amplified libraries were purified using PEG-Sera Mag Speedbeads with 9.19% final PEG concentration. Libraries were quantified using a Qubit HS DNA assay (Invitrogen) and equal molar amounts were pooled. Each pool was quantified for sequencing using the Kapa SYBR Fast Complete Universal qPCR kit (Kapa Biosystems).

### ChIP-seq data analysis

To identify peaks present in ChIP samples that were not present in sequenced input DNA, we used MACS2 version 2.0.10.20131010 (ref. 29). Peaks (5,599) were identified in replicate 1 and 19,642 peaks were identified in replicate 2 at a false discovery rate of 0.05 (Supplementary Data 4). The difference in peak number between replicates was most likely due to the greater amount of chromatin used in the second replicate (1,460 μg versus 675 μg of chromatin) increasing the efficiency of the replicate 2 ChIP compared with replicate 1 ChIP. Quality control statistics for the two replicates were similar (Supplementary Table 3).

Despite differences in the total peak numbers, the peak regions identified by both replicates showed strong concordance. Specifically, 99% of the peak regions identified in replicate 1 also had peaks in replicate 2. Consistent with the notion that both replicates measure the same underlying biology, the peaks that were most likely to reflect true signals (that is, peaks with more significant *P* values) were more likely to be identified in both replicates than peaks that were more likely to be noise (that is, peaks with less significant *P* values; Supplementary Fig. 7a). We also calculated Irreproducible Discovery Rates (IDRs)^56^ for each of the peaks in common between both replicates. IDR values estimate the probability that a peak will be irreproducible in future experiments and are provided in Supplementary Data 4. As expected for high-quality data^57^, a clear inflection point was present around the 1% IDR value in a plot of IDR values versus peak rank number (Supplementary Fig. 7b).

Intersects between peak lists were obtained using ChIPseek^24^. Motifs were identified in sequences within 100 bp of the centre of peaks, using ChIPseek's implementation of HOMER version 4.6 (http://homer.salk.edu/homer/motif/). Analysis of motif enrichment in relation to distance from the peak centres was performed using CentriMo version 4.9.1 (ref. 25). Motif co-occurrence statistics were calculated using ChIPModule^26^ with the default PWMs and Lambda file. For analysis with ChIPModule, the *P* value cutoff for finding PWMs was set to 0.01 and a pattern mining support value of 100 was used.

Genes associated with peaks were identified using GREAT^27^ with the ‘basal plus extension' gene association rule (proximal: 5-kb upstream and 5-kb downstream; distal: 1 Mb), including curated regulatory domains. BETA^28^ was used with default settings to calculate regulatory potential scores and rank product scores. The *x* axis of Fig. 2d and Supplementary Fig. 24 correspond to ranking of regulatory potential scores.

### ChIP–qPCR

ChIP validation primers were designed to regions with peaks in at least one replicate of ChIP-seq that were within 5 kb up or downstream of the TSS of a validation set gene. Two sets of positive control primers were designed to amplify regions that were within 5 kb of the *ABCB4* or *ZNF608* TSSs and had peaks in both replicates of ChIP-seq on mutant and WT MEF2B-V5 cells. Negative control ChIP-qPCR was performed on a region without peaks in either ChIP-seq replicate that was also within 5 kb of the TSS of an expressed gene, *CPS1*.

Validation ChIP on HEK293A cells was performed as for sequencing, but using only 170 μg of chromatin with 5 μl of V5 antibody and 56 μl of beads. ChIP on DLBCL cell lines was also performed as for sequencing, except using a Covaris sonicator (2 min, duty cycle 20%, intensity 8, 200 cycles per burst) and 7 μl of isoform A MEF2B antibody (ProSci) with 258 μg of chromatin and 112 μl of beads in 62% IP buffer. qPCR was performed using SYBR Green qPCR master mix (Life Technologies) and a 7900HT Sequence Detection System (ABI) on equal volumes of ChIPed DNA.

### Gel-shift assays

Gel shift assays were performed using purified MEF2B-V5-his. WT isoform A MEF2B was subcloned from a pDONR vector (Invitrogen) into the pDEST42 bacterial expression construct (Invitrogen) using Gateway LR Clonase Enzyme Mix (Invitrogen). The pDEST42 plasmid contains C-terminal V5 and 6 × His tags. The MEF2B-pDEST42 construct was transformed into BL21-AI competent cells (Invitrogen). Cells were grown to an optical density of 0.4 before addition of 1 mM isopropylthiogalactoside and 0.1% arabinose to induce MEF2B expression. Induced cells were grown at 30 °C for 2 h before cells were pelleted. Pellets from 500 ml of bacterial culture were lysed by vortexing with of 10 ml of low imidazole buffer (50 mM (pH 8) NaH_2_PO_4_, 300 mM NaCl, 5 mM imidazole, 0.5 mM AEBSF). Lysates were then sonicated using three 10-s pulses in a XL-2000 sonicator (Misonix) and were passed through a 0.2-μm filter.

MEF2B was purified from lysates using an AKTA Purifier (GE Healthcare) with Unicorn 5.20 (build 500) workstation software. A 1 ml HisTrap FF column (GE Healthcare) was used to bind the His-tagged MEF2B. Low imidazole buffer was used to remove unbound protein. Bound protein was eluted using a gradually increasing ratio of high to low imidazole buffer. High imidazole buffer was identical to the low imidazole buffer (above) except contained 200 mM imidazole. Fractions eluted at 15–30% high imidazole buffer were collected and concentrated using Amicon Ultra-4 centrifugal filter units with a molecular weight limit of 30 kDa (Millipore). Protein was then dialysed into EMSA buffer (50 mM Tris-HCl, 5 mM MgCl, 50 mM NaCl, 5% glycerol, 200 μg ml^−1^ BSA and 0.5 mM dithiothreitol (DTT)) at 4 °C using 2-ml Slide-A-Lyser mini dialysis devices with a 10-K molecular weight cutoff. Buffer was changed after 2 h of dialysis, and dialysis was allowed to continue overnight. Dialysed protein was concentrated using Amicon Ultra 0.5-ml centrifugal filters with a molecular weight limit of 10 kDa (Millipore). An equal volume of EMSA buffer containing 50% glycerol and 3.5 mM DTT was then added to the protein. Purity was assessed by staining protein on a 4–12% Bis-Tris PAGE gel (Invitrogen) with Coomassie Brilliant blue R-250 (Life Technologies).

Probes were annealed by mixing 2.5 μg of each oligonucleotide in 50 μl of 1 × PCR buffer (Sigma), heating to 65 °C for 5 min and cooling gradually to room temperature. Radiolabelling reactions used 1 μl of annealed oligonucleotides with 0.5 U of Klenow DNA Pol I (NEB), 0.5 nmol dATP, dGTP and dTTP (NEB) and 10 μCi of dCTP containing ^32^P (PerkinElmer). The labelling reaction was allowed to proceed for 15 min at room temperature before purification using Illustra MicroSpin G-50 Columns (GE Healthcare). Probe activity was determined using a Bioscan QC-2000 (InterScience).

For gel shift assays, purified protein was combined with 0.1 μg μl^−1^ of poly[d(I-C)] in 23 μl of EMSA buffer for 20 min at room temperature. Reactions used 10 μg of WT MEF2B-V5 and an equivalent amount of mutant MEF2B-V5, determined using western blots. A total 20,000 c.p.m. of probe (∼5 nM) was then added and binding reactions were allowed to proceed for 20 min at room temperature. Binding reactions were loaded into a 6% PAGE gel made using pH 9.25 TBE and were run at 100 V for 90 min in 0.25 × pH 9.25 TBE. The PAGE gel was then transferred to filter paper and dried at 80 °C for 60 min in a 583 Gel Dryer (Bio-Rad). The dried gel was exposed to a phosphor screen overnight and the screen was scanned using a Typhoon FLA 7000 (GE Healthcare).

### Protein lysate production

Nuclear and cytoplasmic fractions were separated by first treating cells with nuclear isolation buffer (150 mM NaCl, 1.5 mM MgCl_2_, 0.5% NP-40, 10 mM Tris-HCl pH 7.5) on ice for 5 min. Nuclei were pelleted by centrifugation (800*g*, 5 min, 4 °C) and washed four times with nuclei isolation buffer before being lysed in 250 mM NaCl, 20 mM NaPO_4_, 30 mM Na pyrophosphate, 10 mM NaF, 0.5% NP-40, 10% glycerol and 1 mM DTT. The resulting nuclear lysate was syringed through a 22-G needle and centrifuged at 13,000*g*, 4 °C, for 30 min to remove insoluble debris.

Whole-cell lysates were obtained by incubating cells at 4 °C for 1 h with buffer containing 20 mM Tris-HCl pH 7.4, 150 mM NaCl and 0.5 mM NP-40, followed by syringing through a 22-G needle. Complete protease inhibitor cocktail (Roche) was added to all lysis buffers immediately before use.

### Western blotting

Protein concentrations in lysates were determined using the BCA Reagent Kit (Thermo Scientific; Pierce). Protein was separated using PAGE (Invitrogen 4–12% Bis-Tris PAGE gels in MOPS buffer) and transferred to polyvinylidene difluoride membranes (Millipore). Two percentage of skim milk in PBS with 0.01% Tween 20 (Sigma) was used for blocking and antibody dilutions. Incubations with primary antibody were at room temperature for 1 h (actin) or at 4 °C overnight (all other antibodies). Secondary antibodies (goat anti-mouse or anti-rabbit IgG-HRP, Santa Cruz) were applied at 1:5,000 for 1 h at room temperature. Chemiluminescence was detected using Clarity ECL or SuperSignal West Femto substrates (Pierce). Blots were imaged using the ChemiDoc MP Imaging System (Bio-Rad). Densitometry was performed using Image Lab Software version 4.1 (Bio-Rad). Full images of all blots shown in main figures are included as supplementary Figures. Band intensities were calculated relative to the untransfected sample, and then normalized to loading control band intensity. All loading controls were probed on the same membrane as was probed for the protein of interest. Western blots were performed on whole-cell lysates unless noted otherwise.

Antibodies used for western blots recognized V5 (Invitrogen R960-25, 1:5,000 dilution), MYC (Invitrogen, R950-25, 1:5,000 dilution), MEF2C (Cell Signaling 5030, 1:1,000 dilution), BCL6 (Santa Cruz, sc-7388, 1:200 dilution), NDRG1 (Sigma N8539, 1:1,000 dilution), FN1 (Genetex GTX112794, 1:1,000 dilution), VIM (Genetex GTX100619, 1:1,000 dilution), CARD11 (Cell Signaling 4440, 1:1,000 dilution), SNAI2 (Cell Signaling C19G7, 1:500 dilution), Histone 3 (Abcam ab1791, 1:1,000 dilution), MEF2A (Santa Cruz sc-10794, 1:200 dilution), MEF2D (Abcam ab32845, 1:1,000 dilution), lamin A/C (Santa Cruz sc-20680, 1:200 dilution), β-tubulin (Santa Cruz sc-9104, 1:200 dilution), TBP (Abcam ab51841, 1:1,000 dilution) and actin (Abcam ab8227, 1:20,000 dilution). The isoform A MEF2B polyclonal rabbit antibody was custom-made by ProSci using the peptide RPGPALRRLPLADGWPR.

### Quantification of TGFβ1

Concentrations of TGFβ1 in cell culture media were assessed using the Quantikine TGFβ1 Immunoassay (R&D Systems) as directed. Cells were plated in 24-well plates at 8 × 10^4^ cells per well and allowed to adhere overnight. Media was then changed to serum-free DMEM and cells were cultured for 24 h before media samples were collected.

### Cell migration assays

A scratch was drawn with a 30-G needle through a monolayer of HEK293A cells that had been 100% confluent for ∼24 h. Media was replaced immediately after scratching. Cells were imaged on an Axiovert 200 fluorescence microscrope using AxioVision release 4.4 software (Zeiss). The area remaining uncovered by cells was calculated 12, 24 and 32 h after scratches were made. Area values were divided by the length of the imaged scratch to give distance values. The distances at 12 h were subtracted from distances at 24 and 32 h, to give the distance migrated over 12 and 20 h, respectively.

### Proliferation and apoptosis assays

Within each biological replicate, four technical replicates of cells were plated at 1 × 10^4^ cells per well in two 24-well plates. Cells in one plate were fixed and stained after 24 h, whereas cells in the other were fixed and stained after 72 h. Cells were fixed by treatment with 4% paraformaldehyde (Electron Microscopy Sciences) for 15 min at room temperature before being stained for 20 min in 1 mg ml^−1^ crystal violet (EMD Millipore). Cells were washed three times with water to remove excess stain and then were lysed in 10% acetic acid (Fisher). Absorbance at 490 nm was measured in a Victor X3 plate reader with 2030 Workstation software (PerkinElmer). Readings from the plate stained at 72 h were normalized to readings from the plate stained at 24 h.

### Mass spectrometry

Nuclear protein fractions were obtained using the ProteoJET Cytoplasmic and Nuclear Fractionation Kit (Fermentas) and were incubated overnight at 4 °C with 8 μl of MEF2B antibody (Abcam 33540). Forty microlitre of Protein G agarose was then added for 2 h. Beads were washed six times in lysis buffer before protein was eluted at 95 °C for 10 min in 2 × SDS PAGE loading dye. Samples were run in 10% Bis-Tris PAGE gels (Invitrogen) in MOPS buffer and stained with Coomassie Brilliant blue R-250 (Life Technologies). Fragments (30–45 kDa) were excised (predicted molecular weight of MEF2B: 39 kDa) and multiple reaction monitoring (MRM) mass spectrometry was used to identify D83 and D83V MEF2B peptides. MRM mass spectrometry was performed using the same methods and instruments as described previously^58^. Three to four MRM transitions for each peptide were used in the MRM analysis. Peptides detected were TNTDILETLK (D83), TNTVILETLK (D83V) and TPPPLYLPTEGR (control peptide).

### DLBCL patient sample analysis

Paired-end RNA-seq data sets and mutation information for DLBCL patient samples and centroblasts were published previously^14^. Only samples classified as GCB in (ref. 14) were included for analysis here, as all validated *MEF2B* mutations in DLBCL were in the GCB subtype. Differential expression analysis of RNA-seq data used DEseq Release 2.13 (Bioconductor) on genes with at least one read in all mutant MEF2B samples or all WT MEF2B samples. Variance in gene expression values appeared similar between groups (Supplementary Fig. 31b), consistent with the assumptions of DEseq. In addition to differential expression analysis, the DLBCL RNA-seq data was used for determining the ratio of isoform A to isoform B *MEF2B* mRNA abundance. *MEF2B* isoform B is identical to isoform A except for the skipping of exon 8. The number of isoform B transcripts was considered proportional to the sum of the number of sense and antisense reads spanning the junction of exon 7 with exon 9. The number of isoform A transcripts was considered proportional to the sum of the number of sense and antisense reads spanning the junction of exon 7 and 8 or exon 8 and 9, divided by two.

### Chemotaxis assays

RMPI media (600 μl, Gibco) containing chemoattractant was added into 24-well plate wells beneath polycarbonate Transwell inserts with 5-μm pores (Corning, 3421). Cells (1 × 10^6^) in 100 μl of RMPI were then added to the insert. Cells (2.5 × 10^5^) from the same cell suspension were added in triplicate to a 96-well plate as input controls. Cells were allowed to migrate for 3.5 h before inserts were removed and 60 μl of alamarBlue (Life Technologies) was added to the well. alamarBlue was also added to input control cells (10 μl into 90 μl of media). alamarBlue-treated cells were incubated for 2 h at 37 °C before fluorescence was measured as described above. Readings were normalized to media only controls before normalization to input controls. For chemotaxis to CXCL12, the Transwell membrane separated 0 from 300 ng ml^−1^ CXCL12 (PeproTech), with 10% FBS present on both sides of the membrane. For chemotaxis to FBS, the Transwell membrane separated 0 from 10% FBS.

## Additional information

**Accession codes:** The microarray data have been deposited in the Gene Expression Omnibus database under accession code GSE67458. The CHiP-seq and RNA-Seq data have been deposited in the Gene Expression Omnibus database under accession code GSE67458.

**How to cite this article**: Pon, J. R. *et al*. *MEF2B* Mutations in Non-Hodgkin Lymphoma Dysregulate Cell Migration by Decreasing MEF2B Target Gene Activation. *Nat. Commun.* 6:7953 doi: 10.1038/ncomms8953 (2015).

## Supplementary Material

Supplementary Information Supplementary Figures 1-32, Supplementary Tables 1-12 and Supplementary References

Supplementary Data 1 Genes differentially expressed in microarray data for WT MEF2B-V5 versus untransfected HEK293A cells.

Supplementary Data 2 Genes differentially expressed in RNA-seq data for WT MEF2B-V5 versus empty vector HEK293A cells.

Supplementary Data 3 IPA cellular function annotation groups enriched in the genes differentially expressed in WT MEF2B-V5 versus untransfected HEK293A cells.

Supplementary Data 4 Genes associated with peaks in replicate 1 or replicate 2 V5 ChIP-seq on WT MEF2B-V5 cells.

Supplementary Data 5 Known motifs identified in MEF2B-V5 ChIP-seq peak regions.

Supplementary Data 6 Candidate direct MEF2B target genes.

Supplementary Data 7 IPA cellular function annotation groups enriched in the 1,141 candidate direct target genes.

Supplementary Data 8 Genes differentially expressed in microarray data for K4E versus WT MEF2B-V5 HEK293A cells.

Supplementary Data 9 Genes differentially expressed in microarray data for Y69H versus WT MEF2B-V5 HEK293A cells.

Supplementary Data 10 Genes differentially expressed in microarray data for D83V versus WT MEF2B-V5 HEK293A cells.

Supplementary Data 11 Genes differentially expressed in microarray data for comparisons of D83V, Y69H, K4E and untransfected cells to WT MEF2B-V5 HEK293A cells.

Supplementary Data 12 Genes differentially expressed in RNA-seq data for K4E versus WT MEF2B-V5 HEK293A cells.

Supplementary Data 13 Genes differentially expressed in RNA-seq data for Y69H versus WT MEF2B-V5 HEK293A cells.

Supplementary Data 14 Genes differentially expressed in RNA-seq data for D83V versus WT MEF2B-V5 HEK293A cells.

Supplementary Data 15 Genes differentially expressed in MEF2B mutant versus WT GCB DLBCL patient samples.

Supplementary Data 16 Genes differentially expressed in GCB DLBCL patient samples versus reactive centroblasts.

Supplementary Data 17 IPA cellular function annotation groups enriched in genes differentially expressed in GCB DLBCL patient samples versus reactive centroblasts.
