# Supplementary material for: MEF2B mutations in non-Hodgkin lymphoma dysregulate cell migration by decreasing MEF2B target gene activation
Source: Nat Commun. 2015 Aug 6;6:7953. doi: 10.1038/ncomms8953 (PMC4918335; doi:10.1038/ncomms8953)
Supplement: Supplementary Information — Supplementary Figures 1-32, Supplementary Tables 1-12 and Supplementary References [file ncomms8953-s1.pdf]

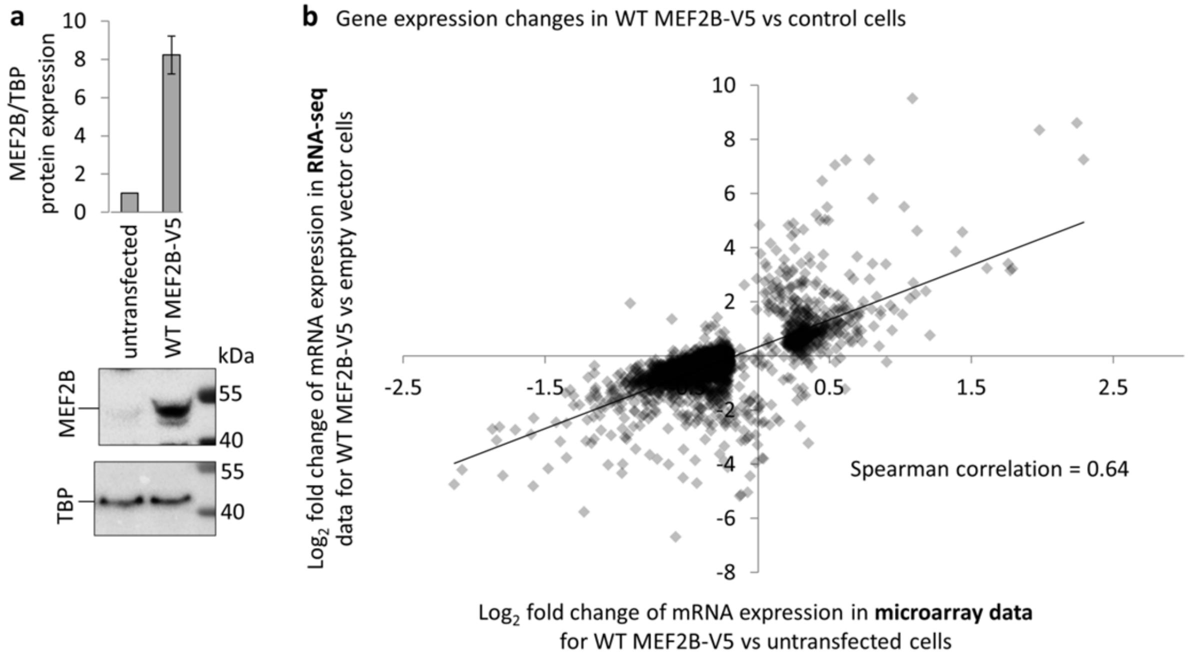

Supplementary Figure 1: Expression microarrays and RNA-seq detected similar alterations in gene expression in response to WT MEF2B-V5 expression

(a) Cells stably transfected with WT MEF2B-V5 have increased abundance of isoform A MEF2B compared to untransfected cells. Shown is the mean fold change in isoform A MEF2B abundance compared to untransfected cells. Error bars represent the s.e.m. of three biological replicates. MEF2B was detected using an antibody that was custom made by ProSci (see methods) to an epitope present only in isoform A MEF2B. **(b)** Fold changes in gene expression between expression microarray and RNA-seq datasets correlate well. Note that microarray data compares WT MEF2B-V5 cells to untransfected cells, whereas RNA-seq data compares WT MEF2B-V5 cells to cells that have been stably transfected with empty vector. Shown are data for genes whose differential expression was statistically significant in at least one of the datasets (B-H adjusted DEseq[1] or eBayes p-values < 0.05).

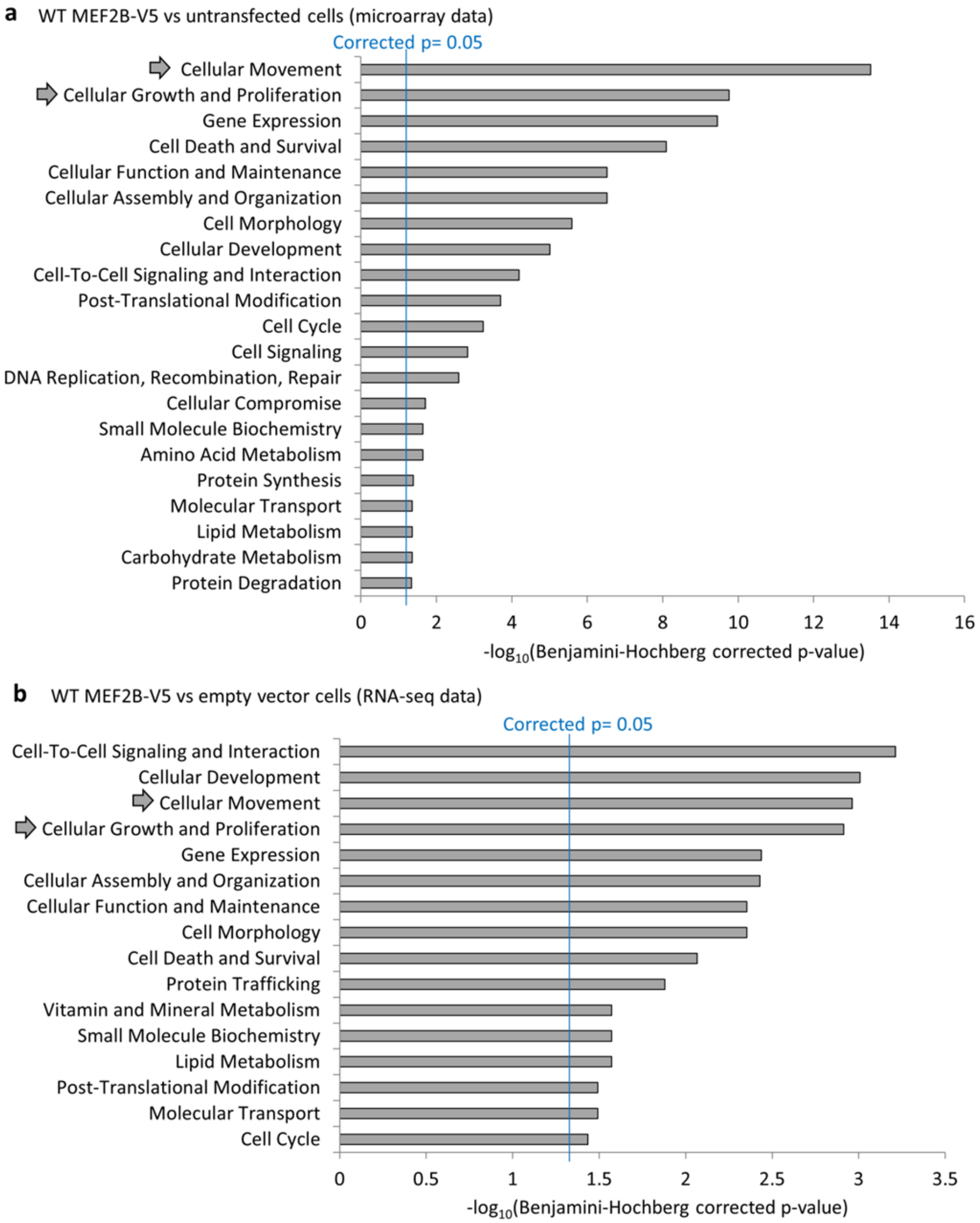

Supplementary Figure 2: Cellular function annotation categories enriched in genes differentially expressed between WT MEF2B-V5 and control cells

The analysed genes were differentially expressed in **(a)** WT MEF2B-V5 expressing versus untransfected cells (B-H adjusted eBayes p-values <0.05 using microarray data) or **(b)** WT MEF2B-V5 expressing versus empty vector cells (B-H adjusted DEseq[1] p-values <0.05 using RNA-seq data). Shown are B-H adjusted right-tailed Fisher exact test p-values for enrichment, calculated using Ingenuity Pathway Analysis. Arrows indicate categories discussed in the main text.

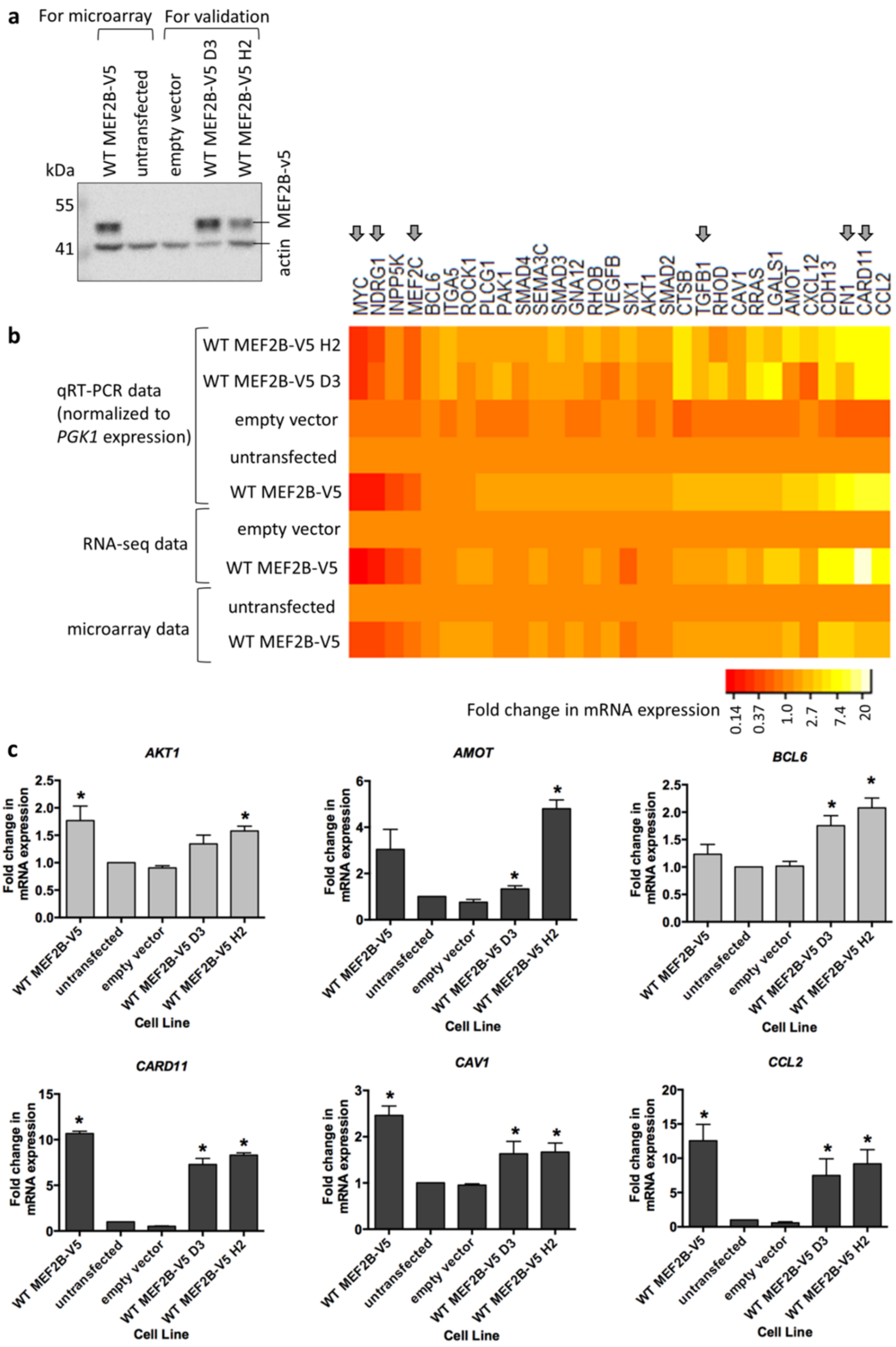
a
For microarray
For validation
WT MEF2B-V5
untransfected
empty vector
WT MEF2B-V5 D3
WT MEF2B-V5 H2
kDa
55
41
MEF2B-v5
actin
b
MYC NDRG1 INPP5K MEF2C BCL6 ITGA5 ROCK1 PLCG1 PAK1 SMAD4 SEMA3C SMAD3 GNA12 RHOB VEGFB SIX1 AKT1 SMAD2 CTSB TGFB1 RHOD CAV1 RRAS LGALS1 AMOT CXCL12 CDH13 FN1 CARD11 CCL2
qRT-PCR data (normalized to PGK1 expression)
WT MEF2B-V5 H2
WT MEF2B-V5 D3
empty vector
untransfected
WT MEF2B-V5
RNA-seq data
empty vector
WT MEF2B-V5
microarray data
untransfected
WT MEF2B-V5
Fold change in mRNA expression
0.14 0.37 1.0 2.7 7.4 20
c
AKT1
AMOT
BCL6
CARD11
CAV1
CCL2
Fold change in mRNA expression
Cell Line
WT MEF2B-V5
untransfected
empty vector
WT MEF2B-V5 D3
WT MEF2B-V5 H2
*

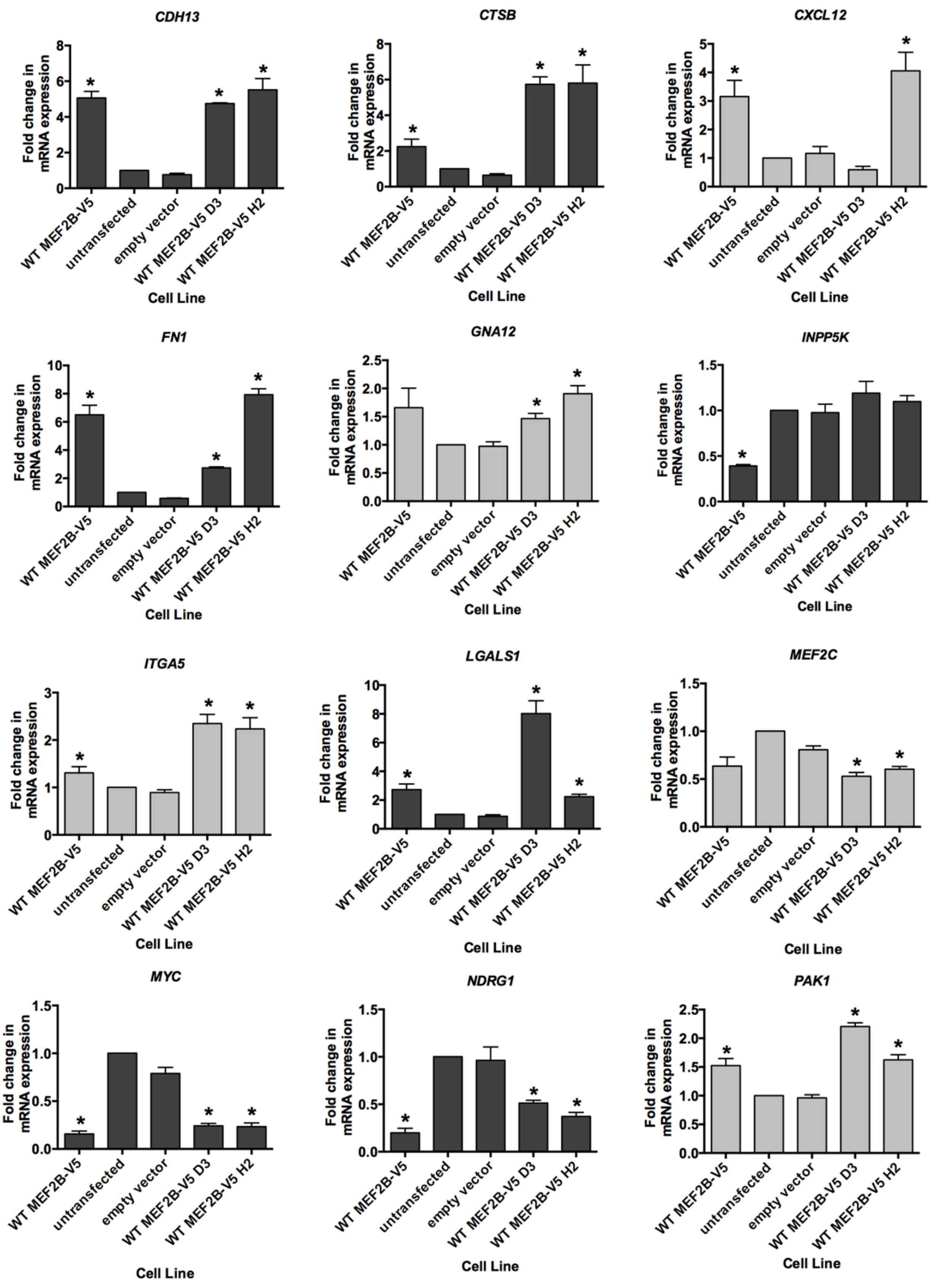

CDH13
CTSB
CXCL12
FN1
GNA12
INPP5K
ITGA5
LGALS1
MEF2C
MYC
NDRG1
PAK1
Fold change in mRNA expression
Cell Line
WT MEF2B-V5
untransfected
empty vector
WT MEF2B-V5 D3
WT MEF2B-V5 H2

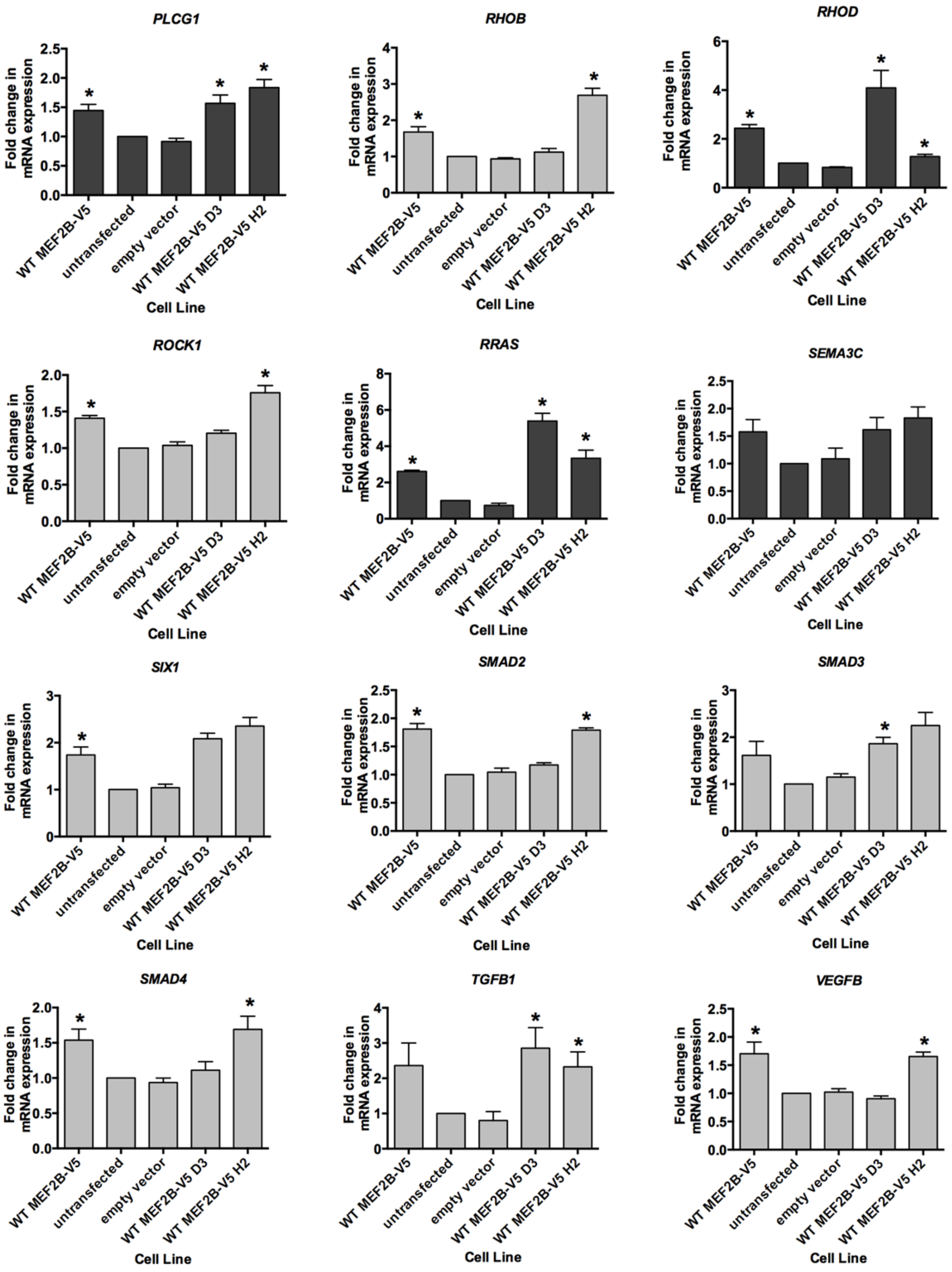

PLCG1
RHOB
RHOD
ROCK1
RRAS
SEMA3C
SIX1
SMAD2
SMAD3
SMAD4
TGFB1
VEGFB
Fold change in mRNA expression
Cell Line
WT MEF2B-V5
untransfected
empty vector
WT MEF2B-V5 D3
WT MEF2B-V5 H2
*

Supplementary Figure 3: Validation of differential gene expression in WT MEF2B-V5 versus untransfected and empty vector cells

(a) MEF2B-V5 abundance in the cell lines used for expression microarrays or validation qRT-PCR. The WT MEF2B-V5 cell line was the monoclonal cell line used for microarrays. WT MEF2B-V5 D3 and H2 were monoclonal cell lines different from the cell line used for microarrays. All WT MEF2B-V5 cell lines were HEK293A cells stably transfected with WT MEF2B-V5. Empty vector cells were stably transfected with empty pcDNA3 vector. **(b)** Fold change in mRNA expression of 30 genes investigated by qRT-PCR for validation of expression microarray data. Shown is the mean fold change across three biological replicates, compared to either untransfected cells (for qRT-PCR and microarray data) or empty vector cells (for RNA-seq data). Arrows indicate genes discussed in the main text. **(c)** Bar plots of the qRT-PCR data shown in (b). Note that *y*-axis scales differ between plots. Error bars represent the s.e.m. of three biological replicates. * $P < 0.05$ in comparison to empty vector cells (Student's two-tailed *t*-test, unpaired). Dark grey plots indicate that the gene was differentially expressed in RNA-seq data for WT MEF2B-V5 versus empty vector cells (B-H adjusted DEseq[1] p-values < 0.05). All 30 genes were differentially expressed in microarray data for WT MEF2B-V5 versus untransfected cells (B-H adjusted eBayes p-values < 0.05).

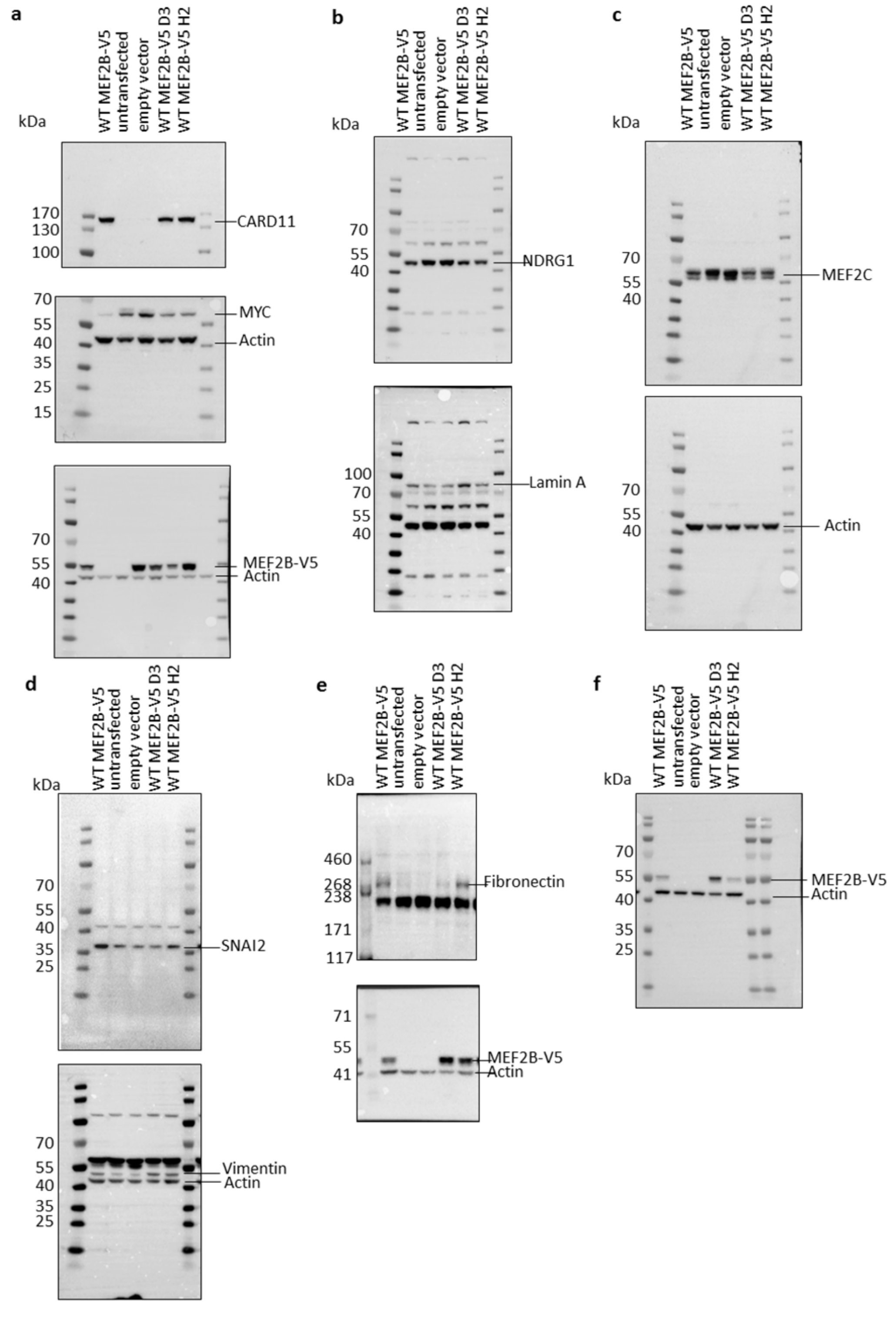

Supplementary Figure 4: Full western blots shown in Figure 1

Portions of the western blots shown in panels **(a)**, **(b)** and **(c)** are shown in Figure 1 (a), (b) and (c), respectively. Portions of the western blots shown in panels **(d)**, **(e)** and **(f)** are shown in Figure 1 (e), (f) and (g), respectively.

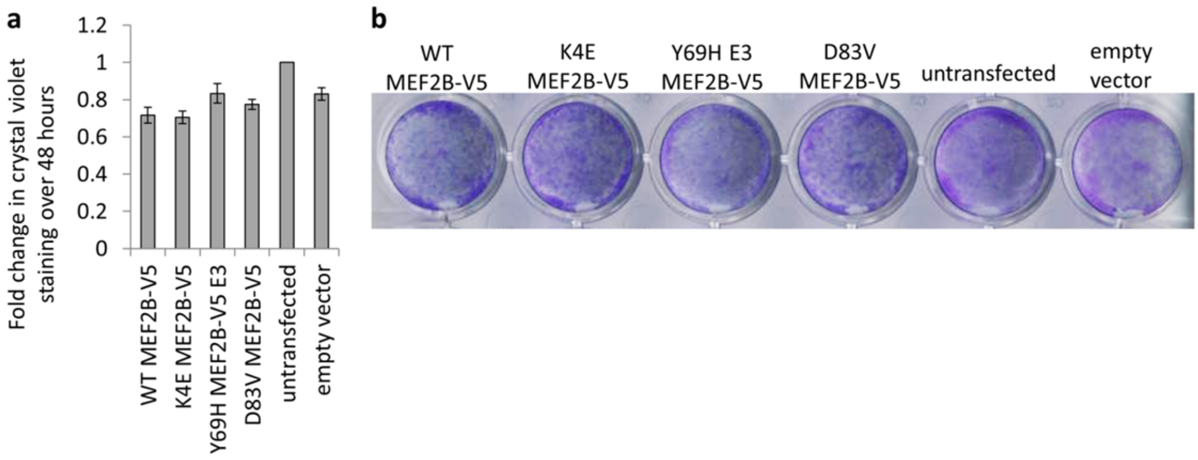

Supplementary Figure 5: Expression of WT or mutant MEF2B-V5 does not affect HEK293A cell proliferation

(a) Quantification of the change in crystal violet staining after 48 hours of cell growth indicates that proliferation rates were not greater for WT MEF2B-V5 cells than for other cell lines. Shown is the mean fold change in absorbance at 490 nm compared to untransfected cells. Error bars represent the s.e.m. of three biological replicates. * $P < 0.05$ (Student's two tailed *t*-test, unpaired) compared to empty vector cells. **(b)** Representative crystal violet staining of cells after 48 hours of cell growth.

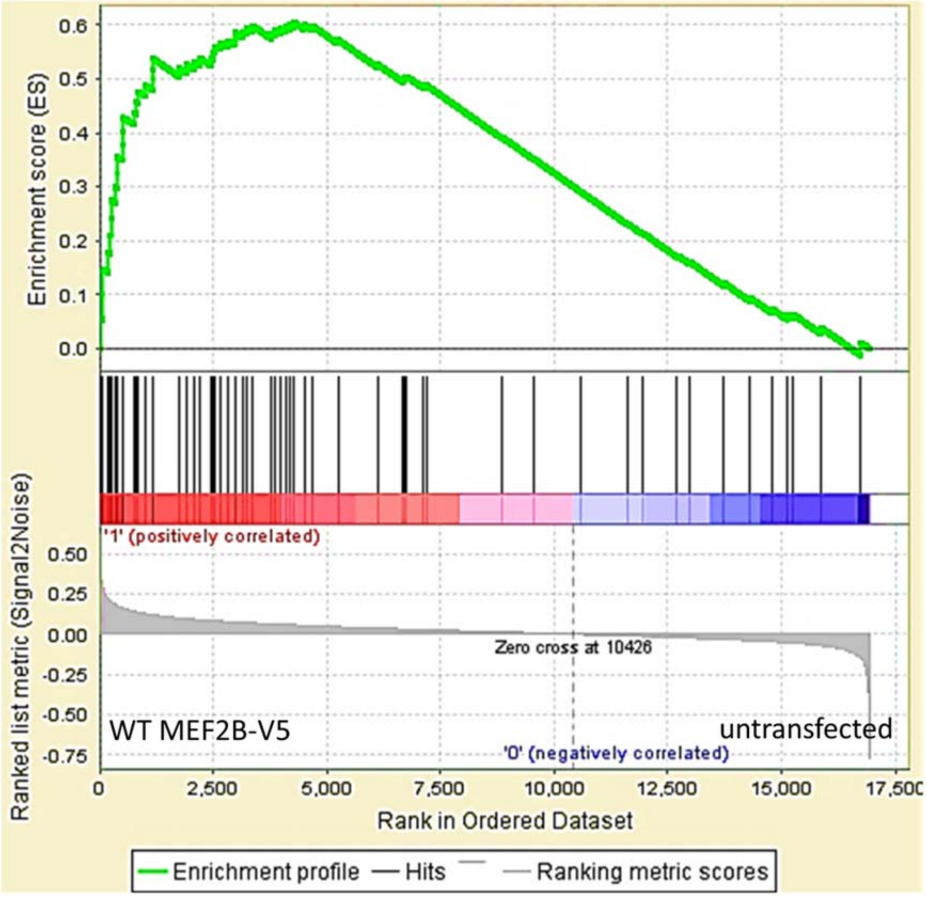

Supplementary Figure 6: Gene Set Enrichment Analysis[2,3] for genes upregulated in EMT

Greater enrichment scores towards the left of the spectrum indicate that genes upregulated in EMT[4] tend to have higher expression in cells with WT MEF2B-V5 than in untransfected cells. All genes with detectable expression in the microarray data were ordered along the *x*-axis according to the magnitude of their expression change in WT MEF2B-V5 versus untransfected cells. Genes with higher expression in WT than untransfected cells are towards the left, and genes with lower expression in WT than untransfected cells are towards the right. Each black vertical line indicates the position of a gene upregulated in EMT in the ordered list of differentially expressed genes. A running-sum statistic for the enrichment of the EMT genes at each position in the ordered list is shown at the top of the figure.

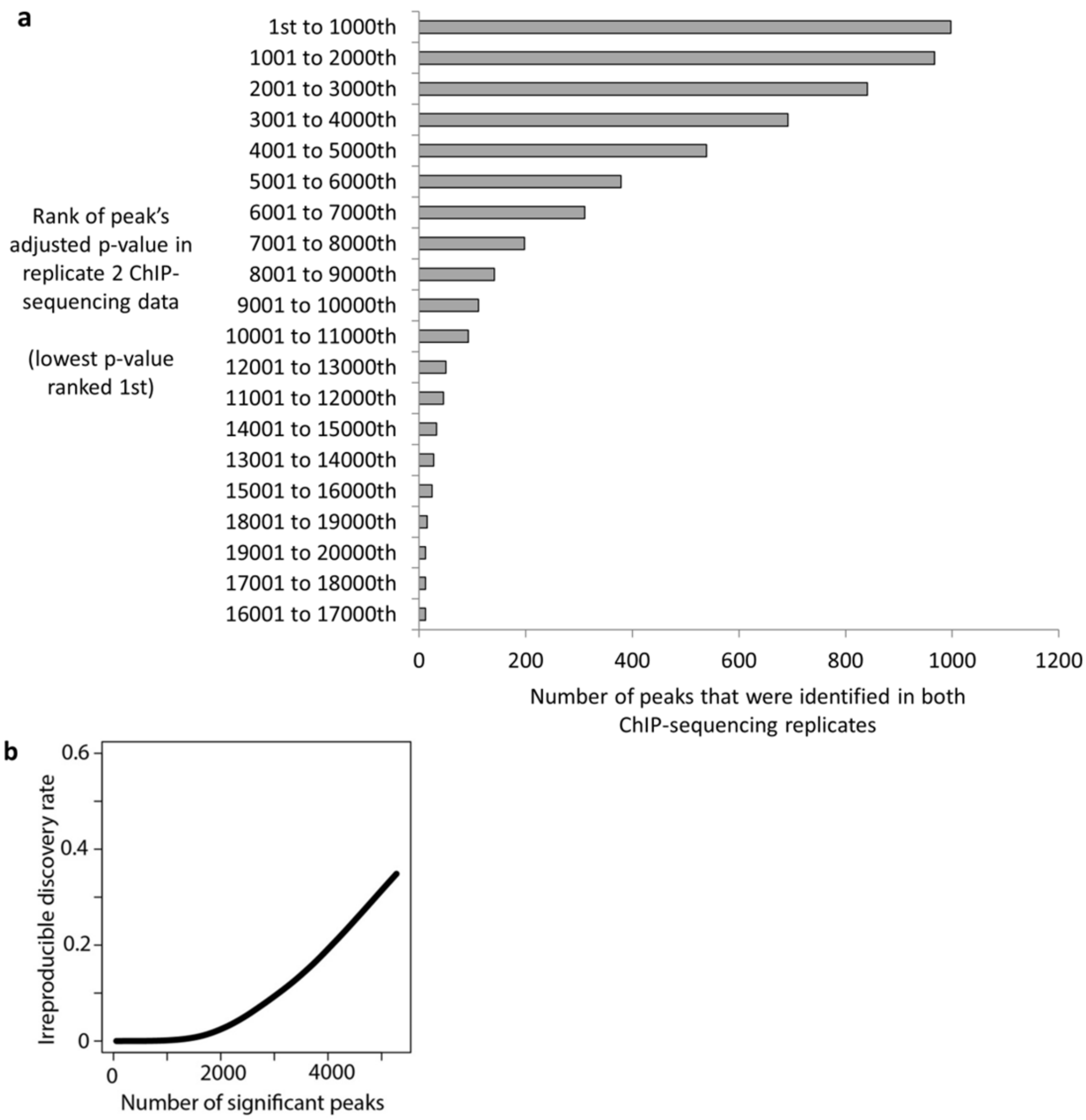

Supplementary Figure 7: Consistency in peak statistics between replicates indicates that WT MEF2B-V5 ChIP-seq data includes reproducibly detectable peaks.

(a) Consistent with the notion that both replicates measure the same underlying biology, the peaks that were most likely to reflect true signals (i.e. peaks with more significant p-values) were more likely to be identified in both replicates than peaks that were more likely to be noise (i.e. peaks with less significant p-values). To produce the plot, all peaks in replicate 2 were ranked by p-value. In each bin of 1,000 rank values there were thus 1,000 peaks from replicate 2 data. However, only a fraction of those peaks were also identified in replicate 1 data. Shown are counts of how many of the replicate 2 peaks were also identified in replicate 1 data, in each bin of rank values. Peaks were identified over input control DNA at a false discovery rate (FDR) of 0.05 using MACS2[5]. **(b)** Peaks in WT MEF2B-V5 ChIP-seq data were enriched for low IDR values, consistent with the notion that many peaks in the ChIP-seq data are highly reproducible.

Significant peaks were ranked by IDR values. Rank numbers are indicated along the *x*-axis. The *y*-axis indicates the IDR value of the peak at each rank position. IDR values estimate the probability that that peak will not be reproducible in future experiments[6]. As expected for high quality data[7], a clear inflection point was present around the 1% IDR value. IDRs were calculated using both replicates of WT MEF2B-V5 ChIP-seq data.

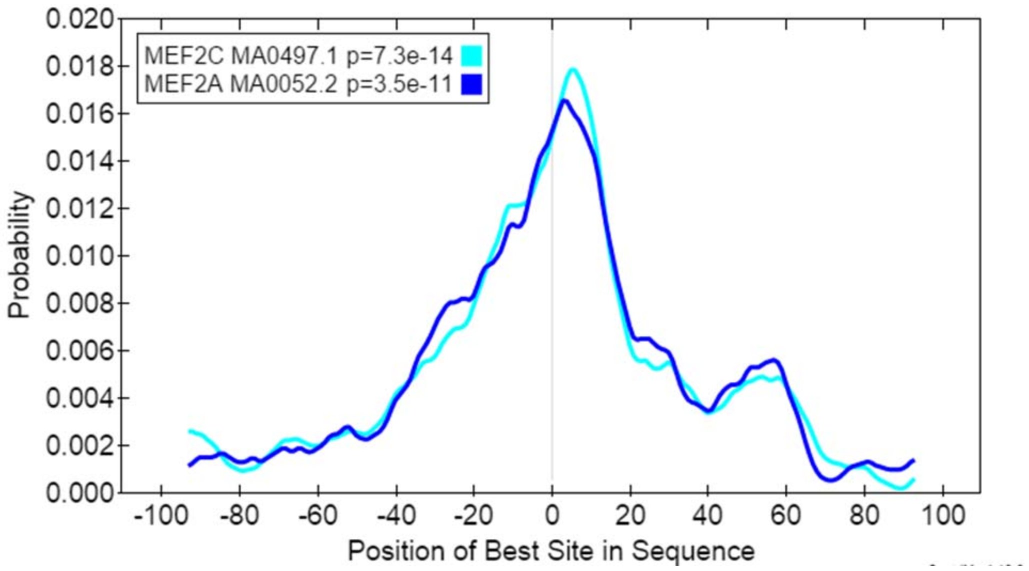

Supplementary Figure 8: MEF2A and MEF2C motifs were centrally enriched in MEF2B-V5 ChIP-seq peaks

MEF2A and MEF2C binding site motifs were more likely to occur near the centre than near the edges of peaks identified in both replicates of MEF2B-V5 ChIP-seq. The plot was produced using CentriMo[8] and shows the probability of a motif occurring at each position within peak regions. Central enrichment p-values are shown in the plot legend. Peaks were identified over input control DNA at a FDR of 0.05 using MACS2[5].

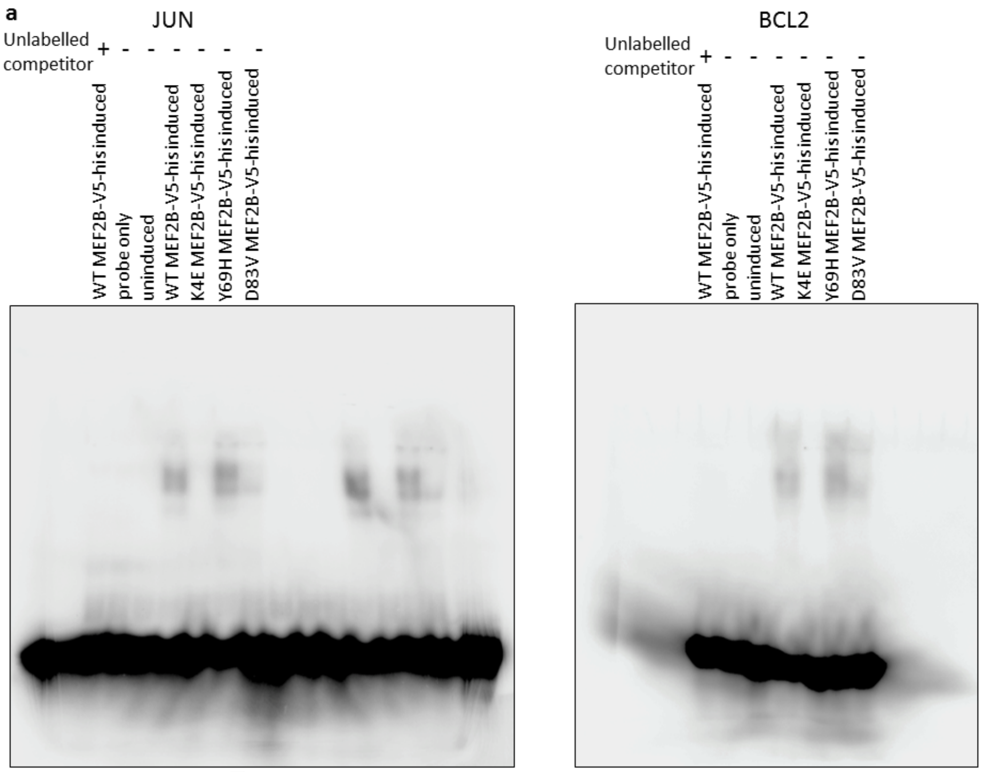

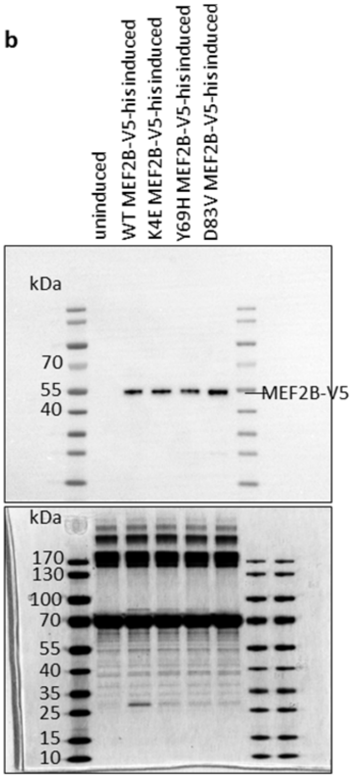

Supplementary Figure 9: Full gel images and western blots shown in Figures 2 and 5

Portions of the gels and western blots shown in **(a)** and **(b)** are shown in Figures 2b and 5a.

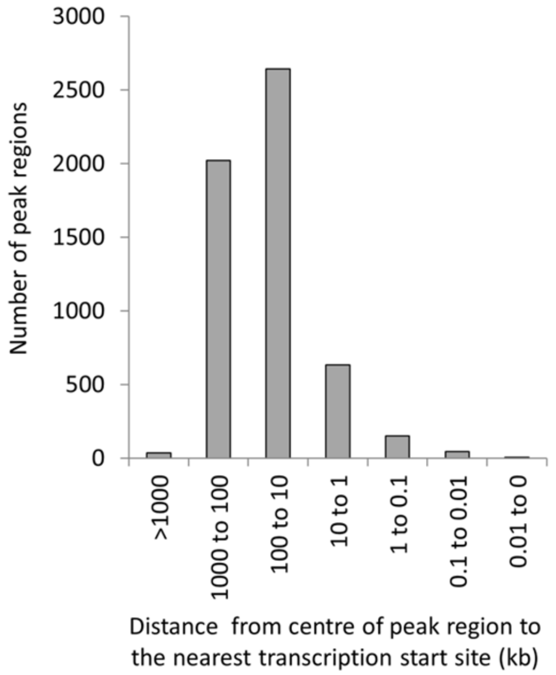

Supplementary Figure 10: The majority of regions with peaks in both replicates of WT MEF2B-V5 ChIP-seq were located further than 10 kb up or downstream of TSSs

GREAT[9] was used to match each peak region to the single nearest gene within 1 Mb up or downstream. This method matched 99.3% of the peak regions to genes. ChIP-seq peaks were identified compared to input control DNA at a FDR of 0.05 using MACS2[5].

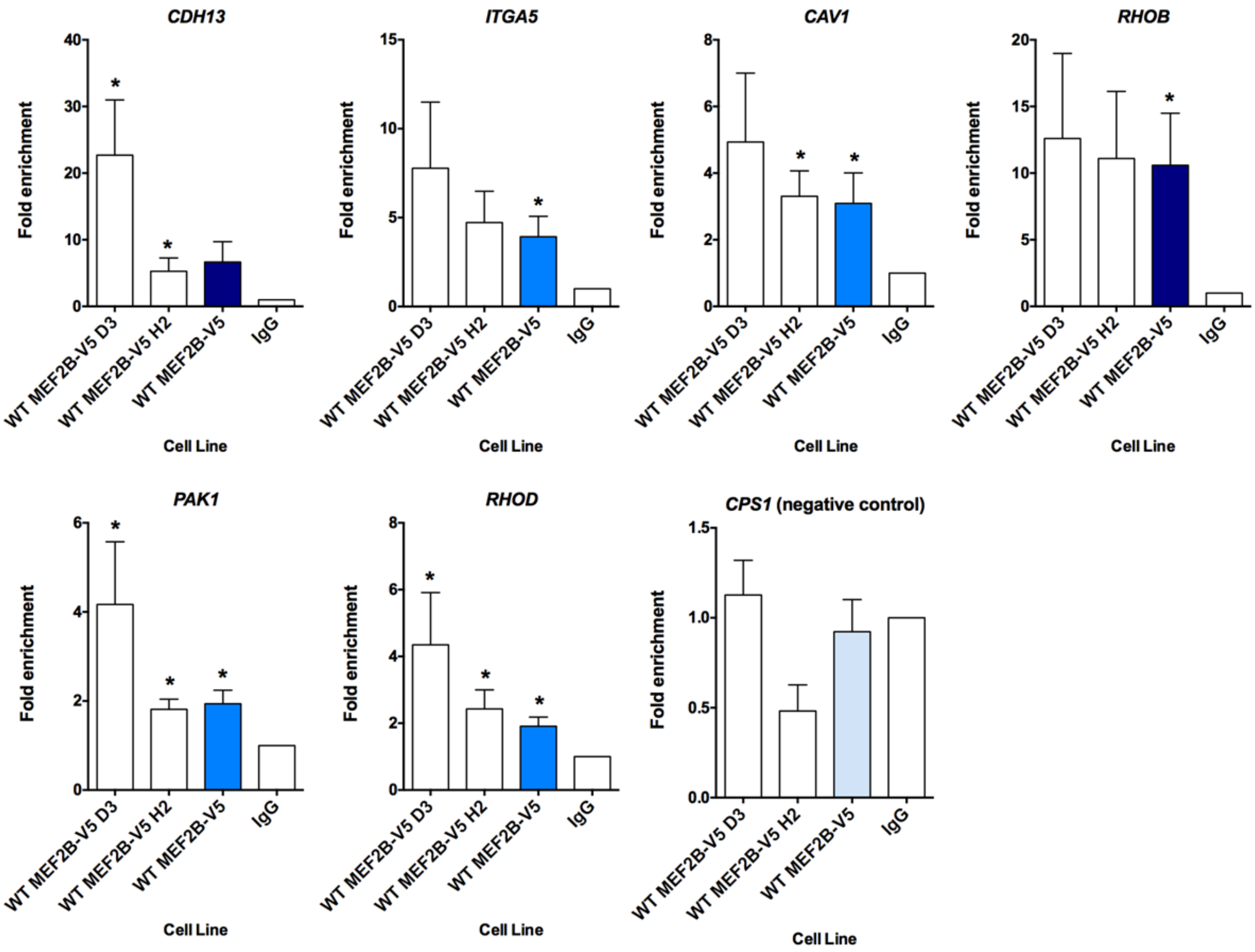

Supplementary Figure 11: ChIP-qPCR validation of V5 ChIP-seq on WT MEF2B-V5 cells

Shown are the mean fold enrichments of DNA regions in three biological replicates of ChIP-qPCR. Note that *y*-axis scales differ between plots. Plot titles indicate genes whose TSS is within 5 kb up or downstream of the DNA region assessed. All ChIPs used V5 antibody except the 'IgG' sample, which used normal mouse immunoglobulin on chromatin from the WT MEF2B-V5 cell line. Fold enrichment was calculated compared to enrichment of an intergenic region not expected to interact with MEF2B, then normalized to fold enrichment in ChIP-qPCR using normal immunoglobulin. The WT MEF2B-V5 cell line was the monoclonal cell line used for ChIP-seq. WT MEF2B-V5 D3 and H2 were monoclonal cell lines different from the cell line used for ChIP-seq. All WT MEF2B-V5 cell lines were HEK293A cells stably transfected with WT MEF2B-V5. The colour of the WT MEF2B-V5 bars indicates how many replicates of V5 ChIP-seq on WT MEF2B-V5 cells had a peak in that region (light blue: no replicates; medium blue: 1 replicate; dark blue: both replicates; FDR 0.05 using MACS2[5]). Error bars represent the s.e.m. * $P < 0.05$ (Student's two tailed *t*-test, unpaired) compared to ChIP using normal immunoglobulin.

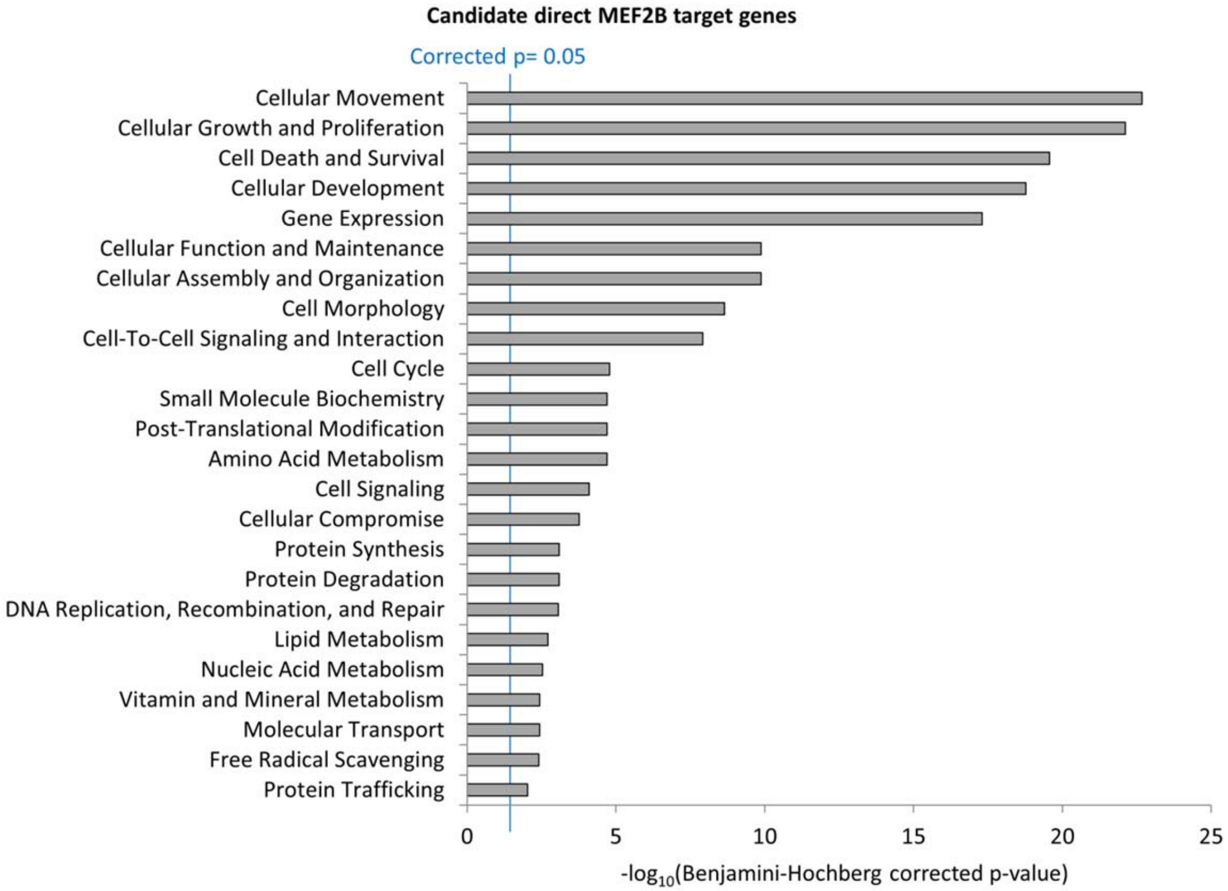

Supplementary Figure 12: Cellular function annotation categories enriched in the 1,141 candidate direct MEF2B target genes

Shown are B-H adjusted right-tailed Fisher exact test p-values for enrichment, calculated using Ingenuity Pathway Analysis.

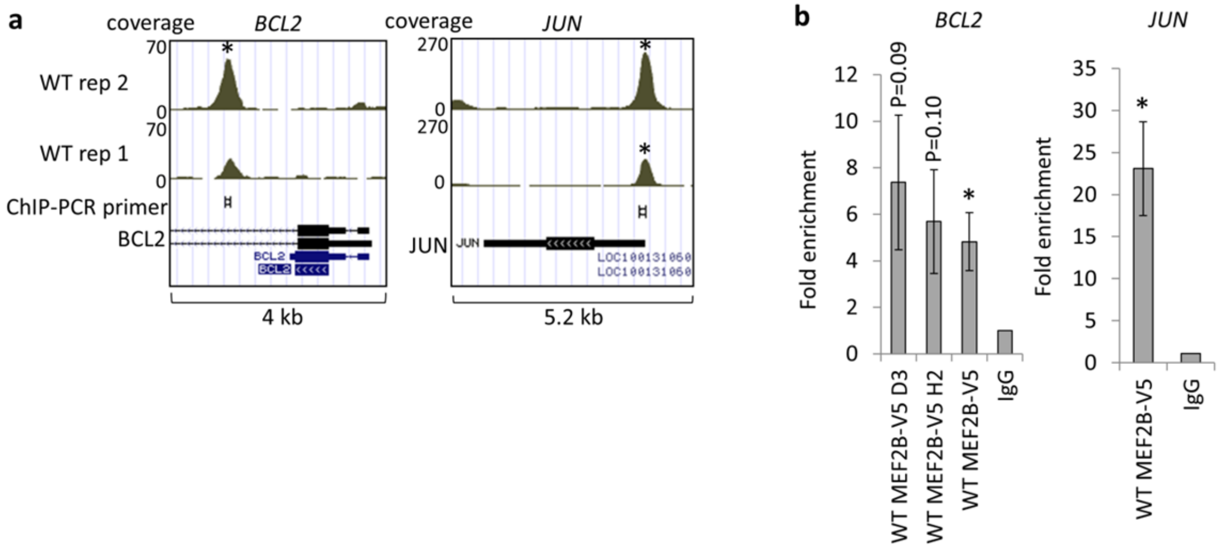

Supplementary Figure 13: MEF2B promotes *BCL2* and *JUN* expression

(a) Shown is the sequencing coverage of regions near the TSSs of *BCL2* and *JUN* in each replicate of V5 ChIP-seq on WT MEF2B-V5 cells. Coverage shows a marked increase in regions where peaks were identified. * indicates the location of a significant peak compared to input control DNA (FDR 0.05 using MACS2[5]). Plots were generated using the UCSC genome browser[10]. **(b)** WT MEF2B-V5 interacts with regions near the TSSs of *BCL2* and *JUN* in ChIP-qPCR. The WT MEF2B-V5 cell line was the monoclonal cell line used for ChIP-seq. WT MEF2B-V5 D3 and H2 were monoclonal cell lines different from the cell line used for ChIP-seq. All ChIPs used V5 antibody except the 'IgG' sample, which used normal mouse immunoglobulin on chromatin from the WT MEF2B-V5 cell line. Fold enrichment of DNA was calculated compared to enrichment of an intergenic DNA region not expected to interact with MEF2B, then normalized to fold enrichment in ChIP-qPCR using normal immunoglobulin. * $P < 0.05$ compared to IgG (Student's two-tailed *t*-test, unpaired). Error bars represent the s.e.m. of three biological replicates.

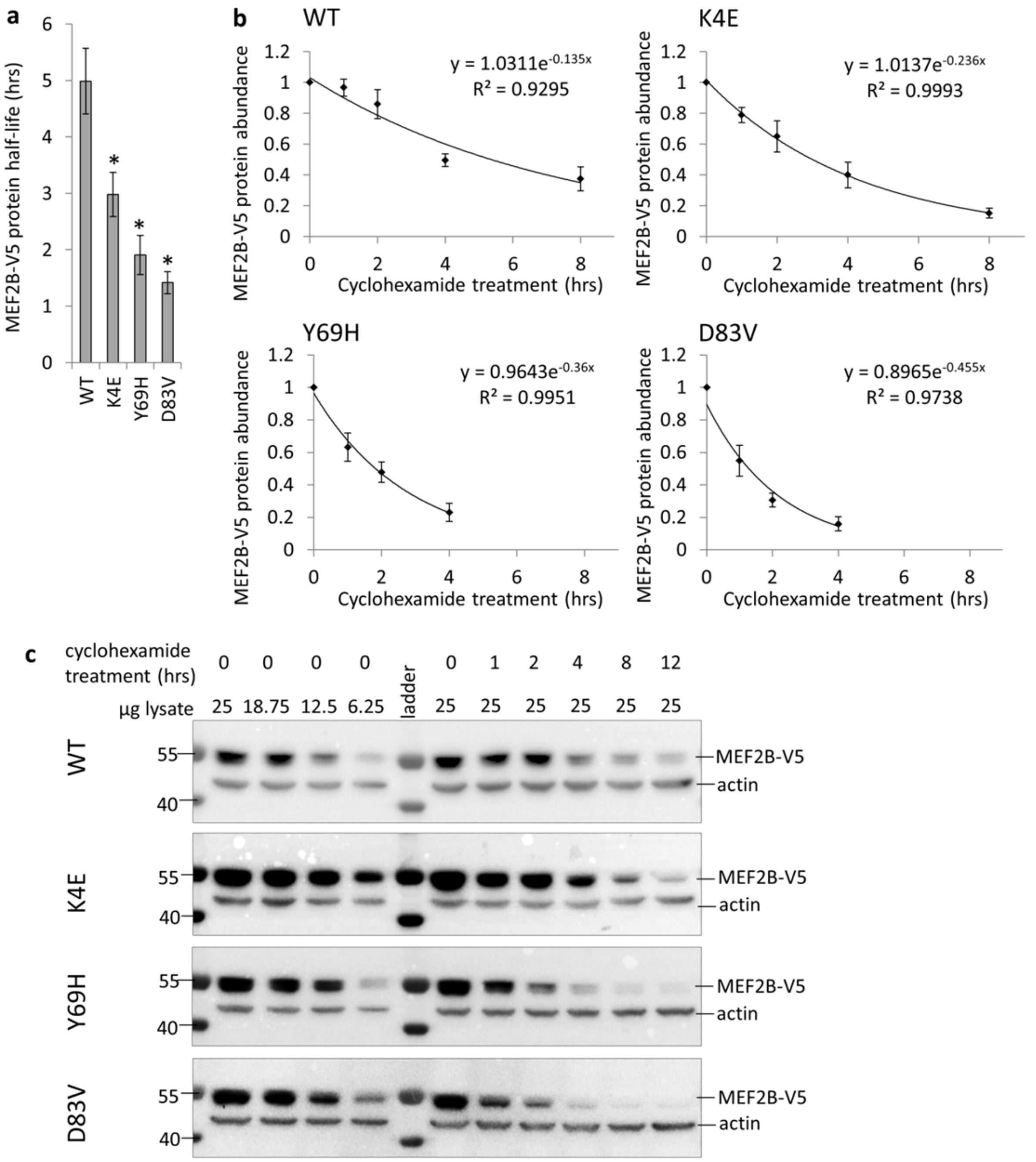

Supplementary Figure 14: K4E, Y69H and D83V mutations reduce MEF2B stability

(a) Mutant MEF2B protein has a reduced half-life. MEF2B-V5 abundance was assessed by western blotting at multiple time points during treatment with the protein synthesis inhibitor cyclohexamide. As cyclohexamide prevents synthesis of new MEF2B protein, the gradual degradation of pre-existing MEF2B over time can be monitored. * $P < 0.05$ compared to WT (Student's two tailed *t*-test, unpaired). **(b)** Degradation curves from which the half-lives were calculated. For **(a)** and **(b)**, error bars represent the s.e.m. of three biological replicates. **(c)** Western blots representative of the data used for **(b).**

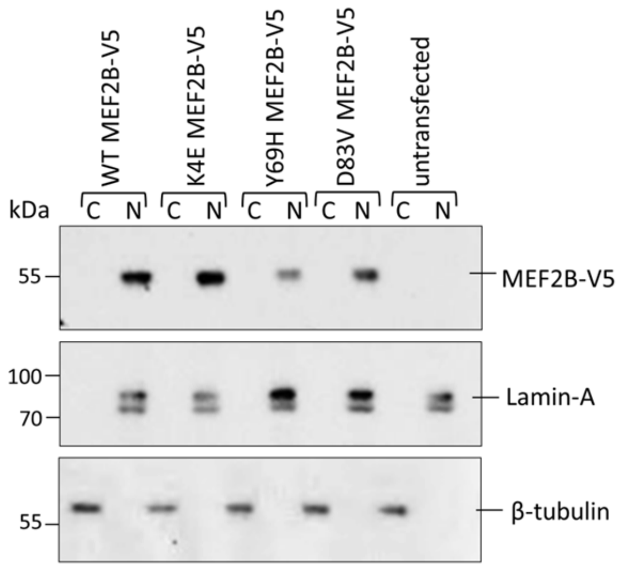

Supplementary Figure 15: WT, K4E, Y69H and D83V MEF2B localize to the nucleus

C: cytoplasm; N: nucleus. Lamin A and β-tubulin were detected as expected in the nuclear or cytoplasmic fractions, respectively.

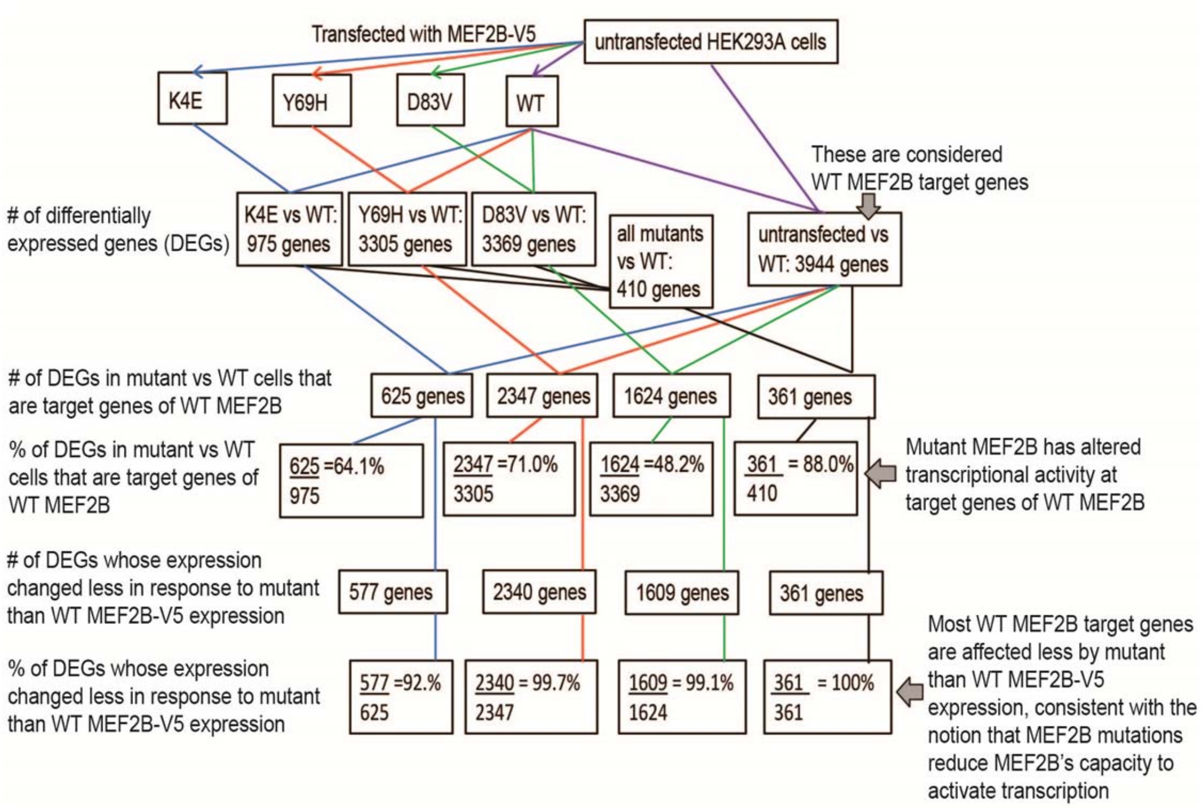

Supplementary Figure 16: Workflow of expression microarray data analysis

The results of this analysis indicate that expression of mutant MEF2B-V5 tended to alter gene expression away from its level in untransfected cells to a lesser extent than expression of WT MEF2B-V5 did. Differentially expressed genes (DEGs) were identified at B-H adjusted eBayes p-values < 0.05. The number of DEGs whose expression changed less in response to mutant than WT MEF2B-V5 expression was considered equal to the number of genes with the same direction of expression change in mutant versus WT MEF2B-V5 cells as in untransfected versus WT MEF2B-V5 cells.

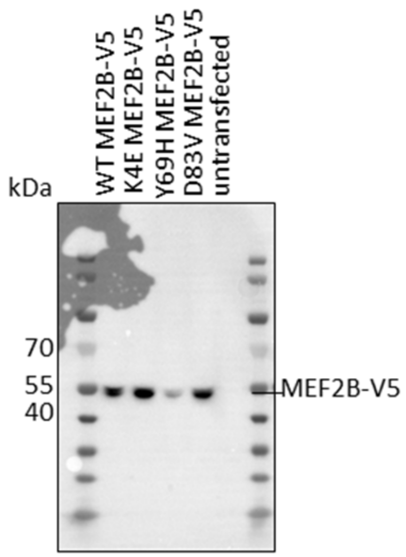

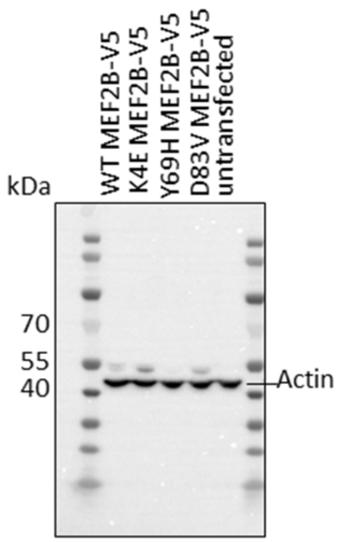

Supplementary Figure 17: Full western blots shown in Figure 3e

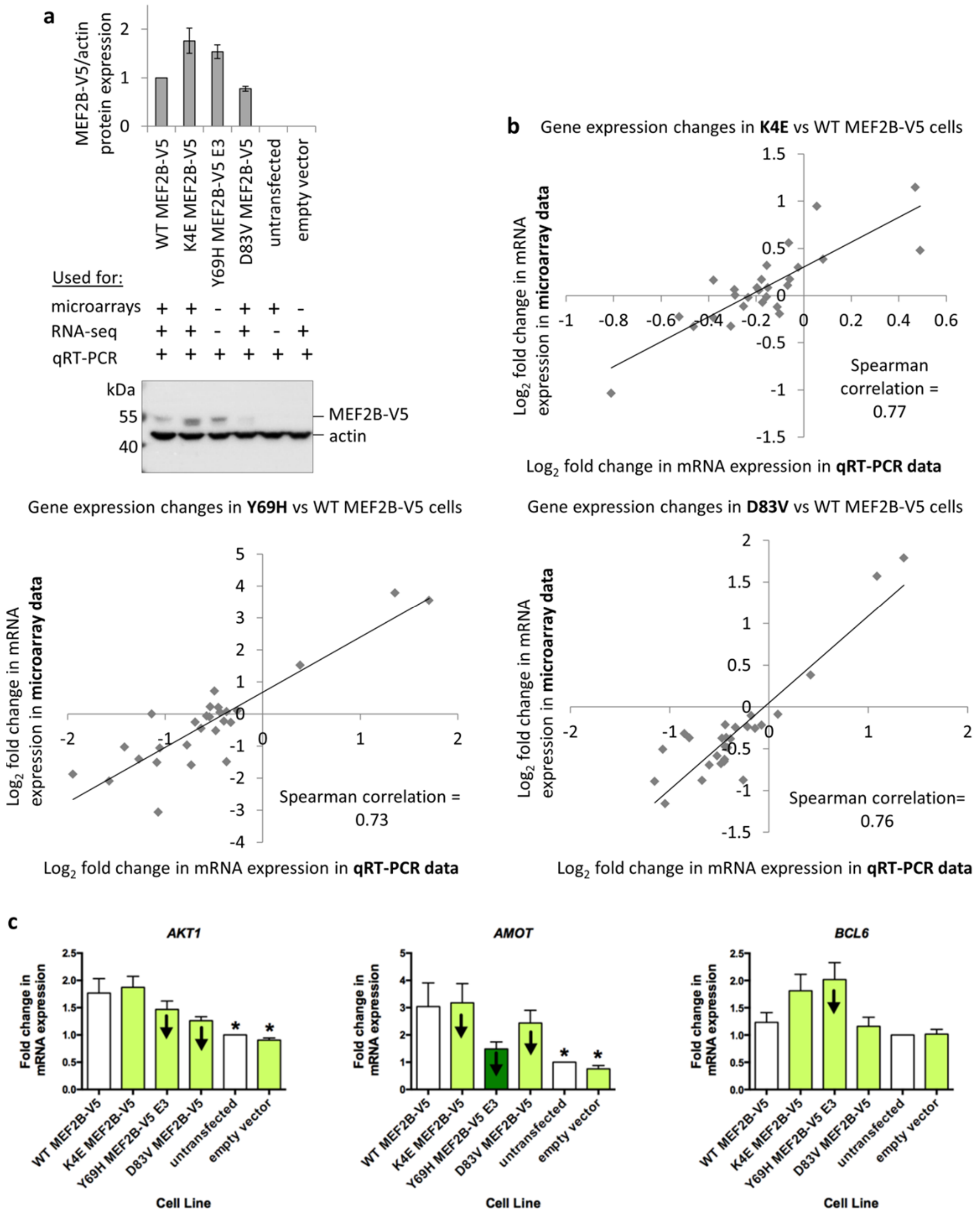
a
MEF2B-V5/actin protein expression
WT MEF2B-V5
K4E MEF2B-V5
Y69H MEF2B-V5 E3
D83V MEF2B-V5
untransfected
empty vector
Used for:
microarrays + + - + + -
RNA-seq + + - + - +
qRT-PCR + + + + + +
kDa
55
40
MEF2B-V5
actin
b
Gene expression changes in K4E vs WT MEF2B-V5 cells
Log2 fold change in mRNA expression in microarray data
Log2 fold change in mRNA expression in qRT-PCR data
Spearman correlation = 0.77
Gene expression changes in Y69H vs WT MEF2B-V5 cells
Spearman correlation = 0.73
Gene expression changes in D83V vs WT MEF2B-V5 cells
Spearman correlation= 0.76
c
AKT1
AMOT
BCL6
Fold change in mRNA expression
Cell Line

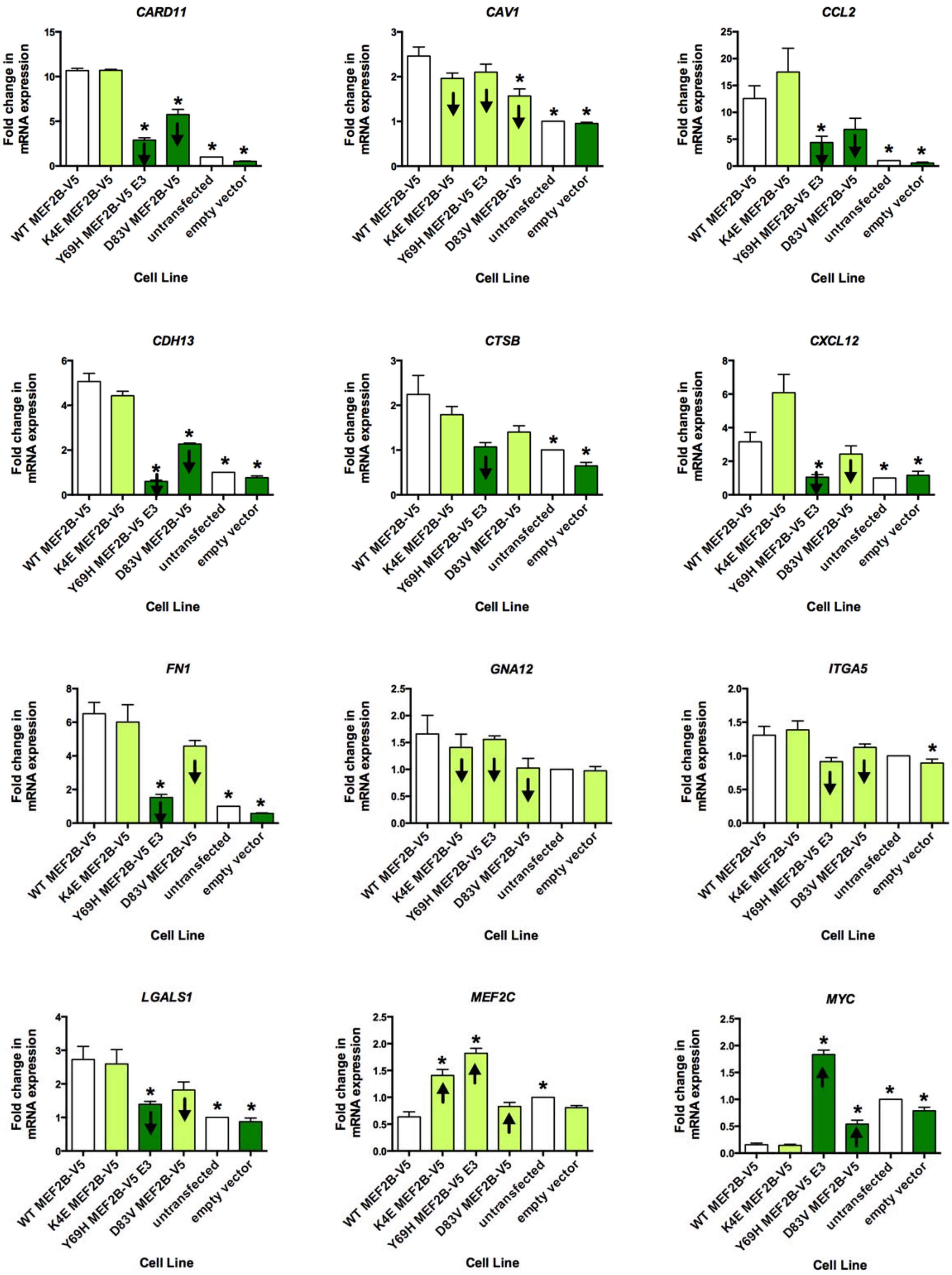
CARD11
CAV1
CCL2
CDH13
CTSB
CXCL12
FN1
GNA12
ITGA5
LGALS1
MEF2C
MYC
Fold change in mRNA expression
WT MEF2B-V5
K4E MEF2B-V5
Y69H MEF2B-V5 E3
D83V MEF2B-V5
untransfected
empty vector
Cell Line

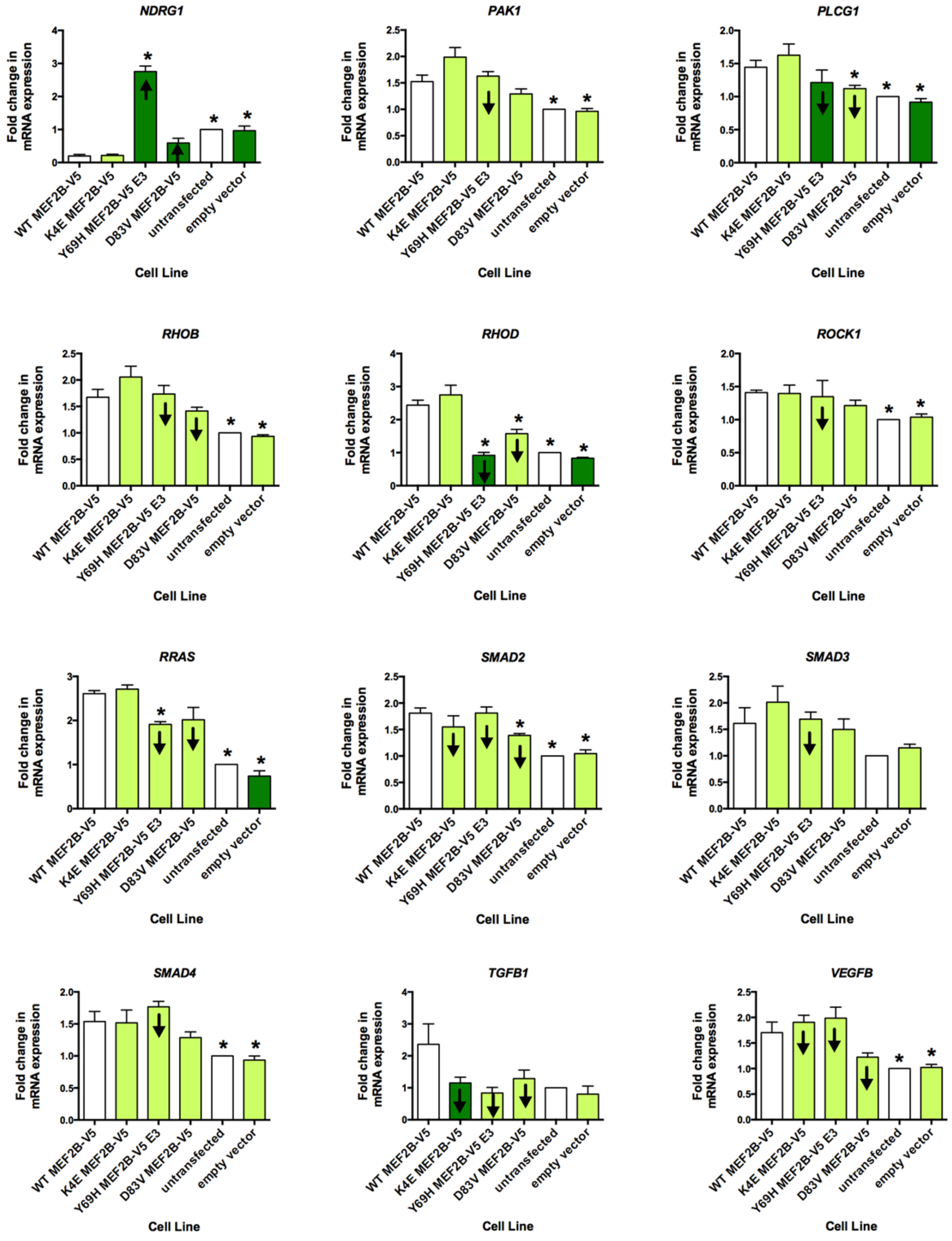
NDRG1
PAK1
PLCG1
RHOB
RHOD
ROCK1
RRAS
SMAD2
SMAD3
SMAD4
TGFB1
VEGFB
Fold change in mRNA expression
WT MEF2B-V5
K4E MEF2B-V5
Y69H MEF2B-V5 E3
D83V MEF2B-V5
untransfected
empty vector
Cell Line

Supplementary Figure 18: Validation of expression microarray data using qRT-PCR on additional cell lines

(a) The mutant MEF2B-V5 cell lines used for validation have similar or greater MEF2B-V5 abundance compared to the WT MEF2B-V5 cells. The Y69H MEF2B-V5 E3 cells are a different monoclonal cell line than the Y69H MEF2B-V5 cells that were used for microarrays and RNA-seq. The K4E, D83V and WT MEF2B-V5 cells are the monoclonal cell line that were used for microarrays and RNA-seq. Error bars represent the s.e.m. of three biological replicates. A representative western blot is shown. MEF2B-V5 was detected using V5 antibody. **(b)** Fold changes in gene expression in mutant versus WT MEF2B-V5 cells produced using qRT-PCR data correlate well with fold changes in gene expression in mutant versus WT MEF2B-V5 cells produced using microarray data. **(c)** qRT-PCR data for fold changes in mRNA expression in mutant MEF2B-V5, WT MEF2B-V5, and empty vector cells compared to untransfected cells. The mRNA expression of each gene was normalized to *PGK1* expression. Note that *y*-axis scales differ between plots. Error bars represent the s.e.m. of three biological replicates. * $P < 0.05$ in comparison to WT MEF2B-V5 cells (unpaired Student's two-tailed *t*-test on qRT-PCR data). Bar colour indicates the B-H adjusted DEseq[1] p-value calculated using RNA-seq data for that cell line compared to WT MEF2B-V5 cells (dark green: < 0.05; light green: > 0.05). Arrows indicate that the gene was differentially expressed in microarray data for that mutant MEF2B-V5 cell line compared to WT MEF2B-V5 cells (B-H adjusted eBayes p-values < 0.05; up arrow = increased expression in mutant versus WT MEF2B-V5 cells; down arrow = decreased expression in mutant versus WT MEF2B-V5 cells).

a

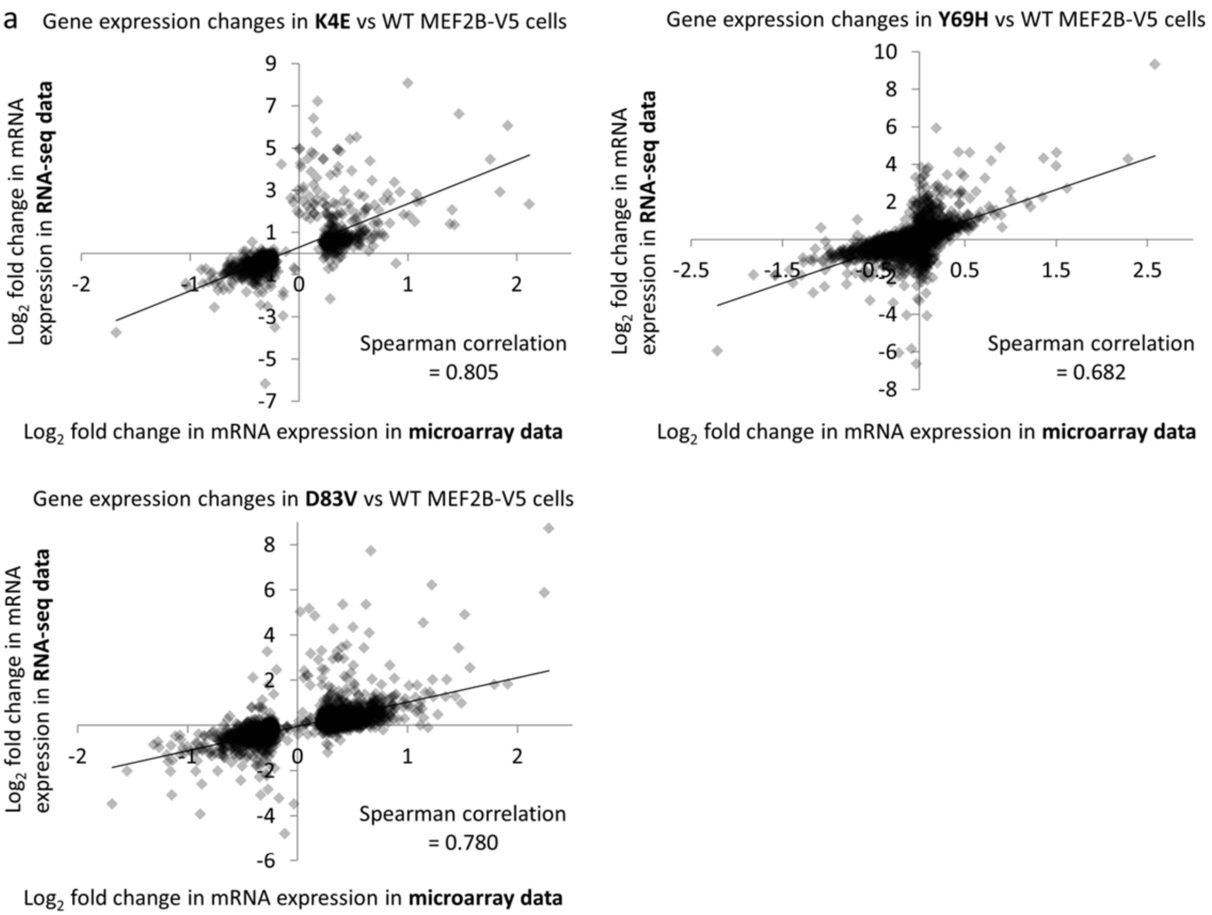

Gene expression changes in **K4E** vs WT MEF2B-V5 cells
Log$_2$ fold change in mRNA expression in **RNA-seq data**
Log$_2$ fold change in mRNA expression in **microarray data**
Spearman correlation = 0.805
Gene expression changes in **Y69H** vs WT MEF2B-V5 cells
Log$_2$ fold change in mRNA expression in **RNA-seq data**
Log$_2$ fold change in mRNA expression in **microarray data**
Spearman correlation = 0.682
Gene expression changes in **D83V** vs WT MEF2B-V5 cells
Log$_2$ fold change in mRNA expression in **RNA-seq data**
Log$_2$ fold change in mRNA expression in **microarray data**
Spearman correlation = 0.780

b

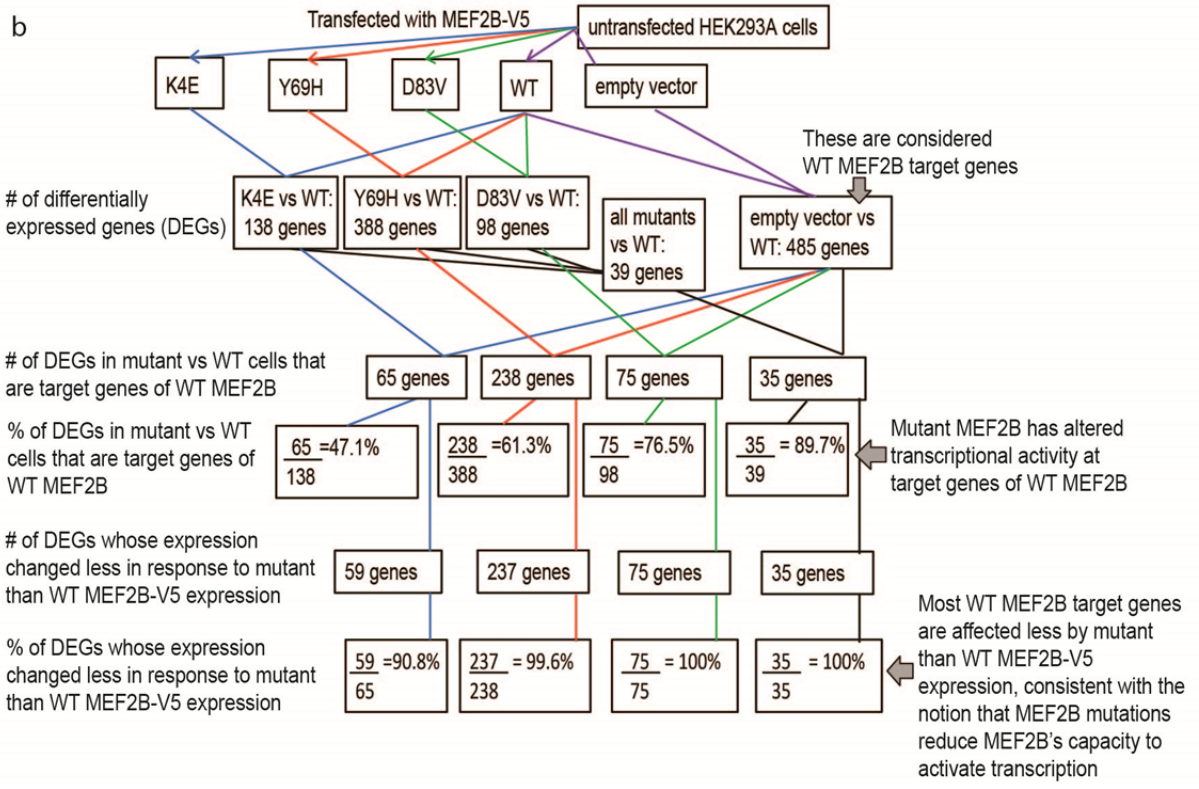

Transfected with MEF2B-V5
untransfected HEK293A cells
K4E
Y69H
D83V
WT
empty vector
These are considered WT MEF2B target genes
# of differentially expressed genes (DEGs)
K4E vs WT: 138 genes
Y69H vs WT: 388 genes
D83V vs WT: 98 genes
all mutants vs WT: 39 genes
empty vector vs WT: 485 genes
# of DEGs in mutant vs WT cells that are target genes of WT MEF2B
65 genes
238 genes
75 genes
35 genes
% of DEGs in mutant vs WT cells that are target genes of WT MEF2B
65/138 =47.1%
238/388 =61.3%
75/98 =76.5%
35/39 = 89.7%
Mutant MEF2B has altered transcriptional activity at target genes of WT MEF2B
# of DEGs whose expression changed less in response to mutant than WT MEF2B-V5 expression
59 genes
237 genes
75 genes
35 genes
% of DEGs whose expression changed less in response to mutant than WT MEF2B-V5 expression
59/65 =90.8%
237/238 = 99.6%
75/75 = 100%
35/35 = 100%
Most WT MEF2B target genes are affected less by mutant than WT MEF2B-V5 expression, consistent with the notion that MEF2B mutations reduce MEF2B's capacity to activate transcription

Supplementary Figure 19: RNA-seq data for mutant versus WT MEF2B-V5 cells supports the conclusions drawn from the microarray data

(a) Fold changes in gene expression between expression microarray and RNA-seq datasets correlate well. Shown are data for genes whose differential expression was statistically significant in at least one of the datasets (B-H adjusted DEseq[1] or eBayes p-values < 0.05). **(b)** Consistent with conclusions from the microarray data, analysis of RNA-seq data indicates that expression of mutant MEF2B-V5 tended to alter gene expression away from its level in untransfected cells to a lesser extent than expression of WT MEF2B-V5 did. Differentially expressed genes (DEGs) were identified at B-H adjusted DEseq[1] p-values < 0.05. The number of DEGs whose expression changed less in response to mutant than WT MEF2B-V5 expression was considered equal to the number of genes with the same direction of expression change in mutant versus WT MEF2B-V5 cells as in empty vector versus WT MEF2B-V5 cells.

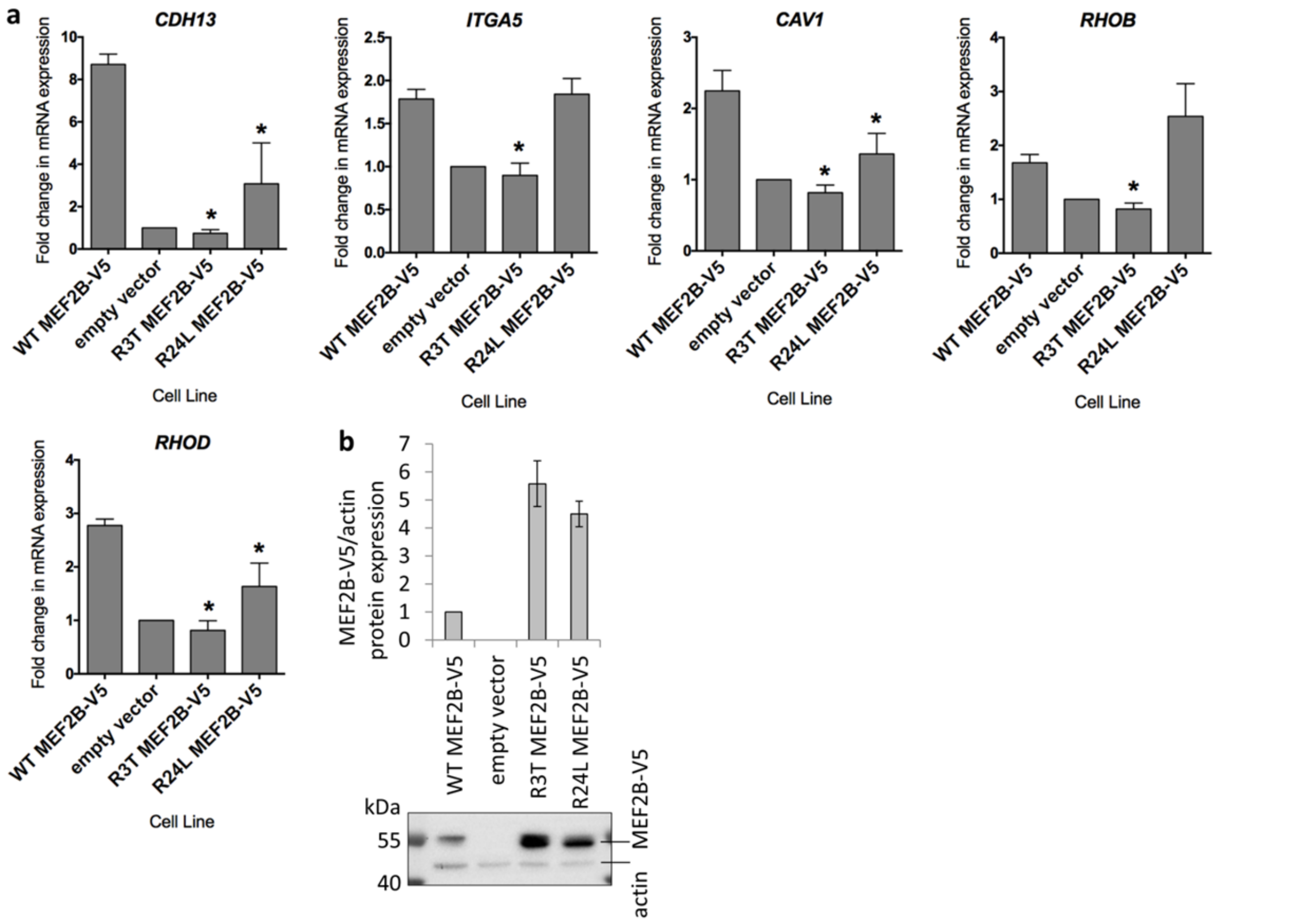

Supplementary Figure 20: R3T and R24L mutations decrease MEF2B's capacity to activate transcription

(a) Expression of R3T or R24L MEF2B-V5 in HEK293A cells tended to increase target gene expression away from its level in untransfected cells to a lesser extent than expression of WT MEF2B-V5. Shown is the mean fold change in expression compared to cells transfected with empty vector. Note that *y*-axis scales differ between plots. Error bars represent the s.e.m. of three biological replicates. * $P < 0.05$ (Student's two tailed *t*-test, unpaired) compared to WT MEF2B-V5 cells. Data were produced using qRT-PCR **(b)** MEF2B-V5 protein abundance is greater in R3T and R24L MEF2B-V5 cells than in WT MEF2B-V5 cells. Relative abundance was calculated compared to WT MEF2B-V5 cells using densitometry on three biological replicates of western blots. A representative western blot is shown. Error bars represent the s.e.m.

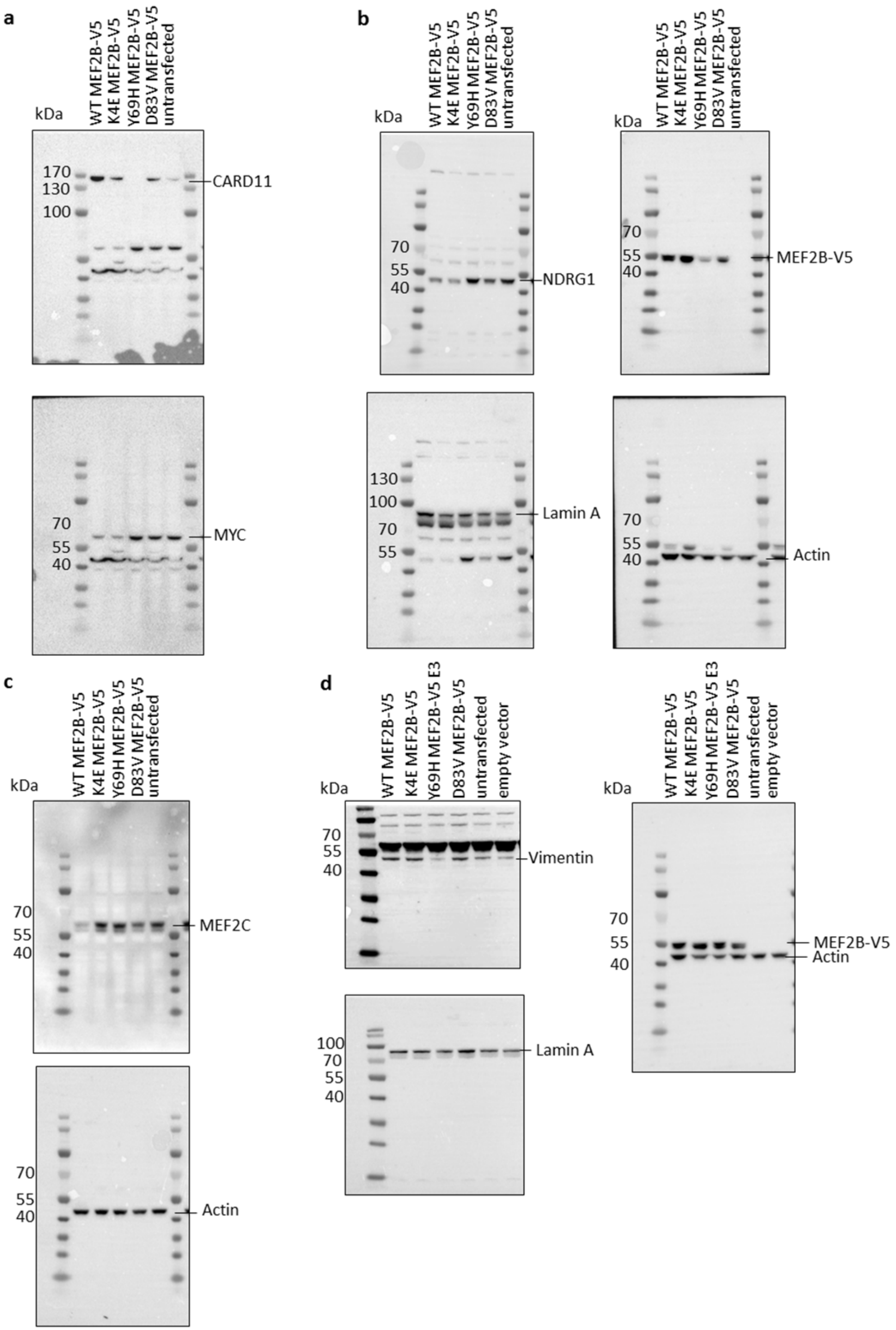

a
WT MEF2B-V5
K4E MEF2B-V5
Y69H MEF2B-V5
D83V MEF2B-V5
untransfected
kDa
170
130
100
CARD11
70
55
40
MYC
b
NDRG1
MEF2B-V5
Lamin A
Actin
c
MEF2C
Actin
d
Y69H MEF2B-V5 E3
empty vector
Vimentin
Lamin A
MEF2B-V5
Actin

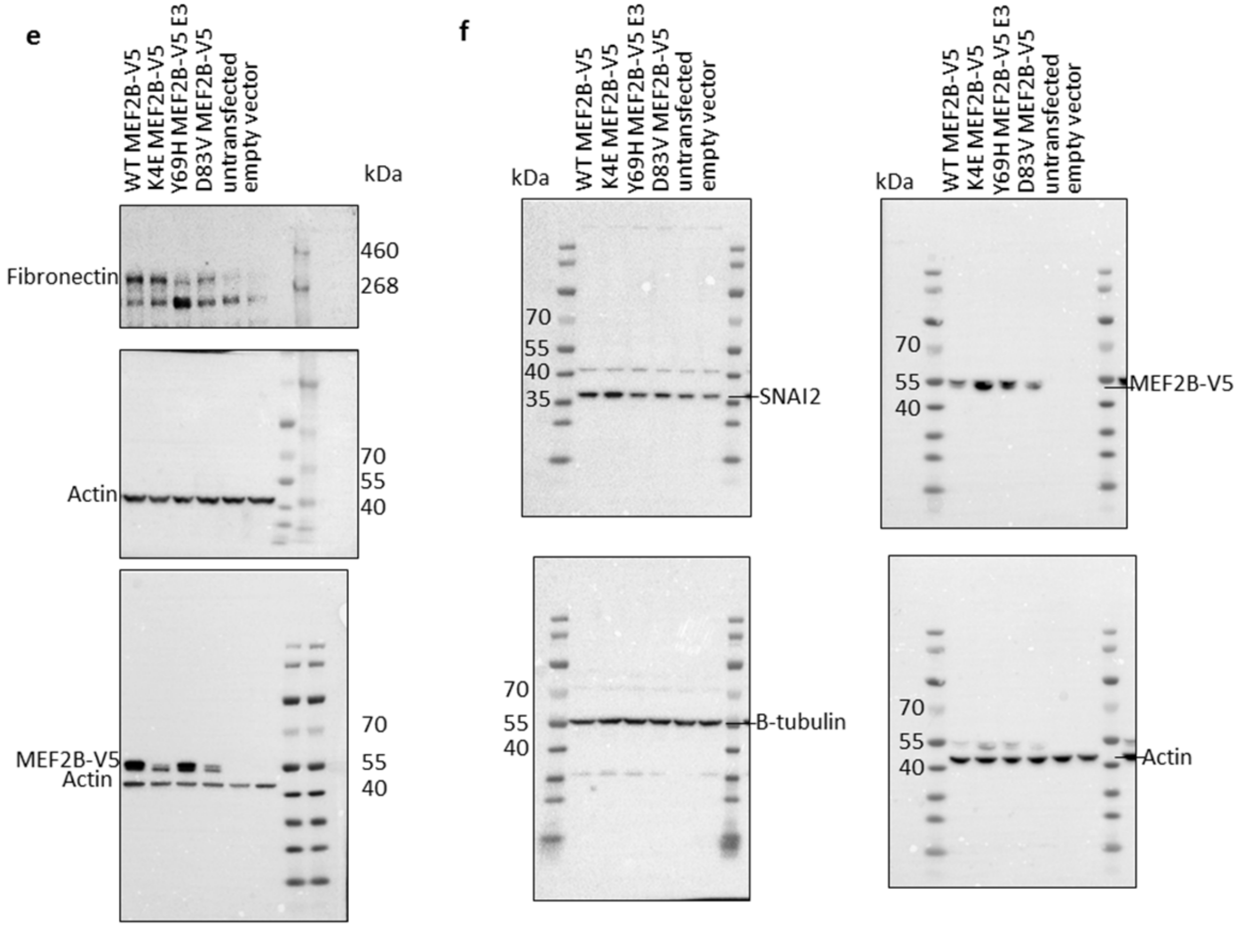

Supplementary Figure 21: Full western blots shown in Figure 4

Panel letters correspond to the panels in Figure 4 that show portions of these western blots. Note that the MEF2B-V5 blot in Figure 4a is the same as that shown in Figure 3e, and is thus shown in full in Supplementary Figure 17.

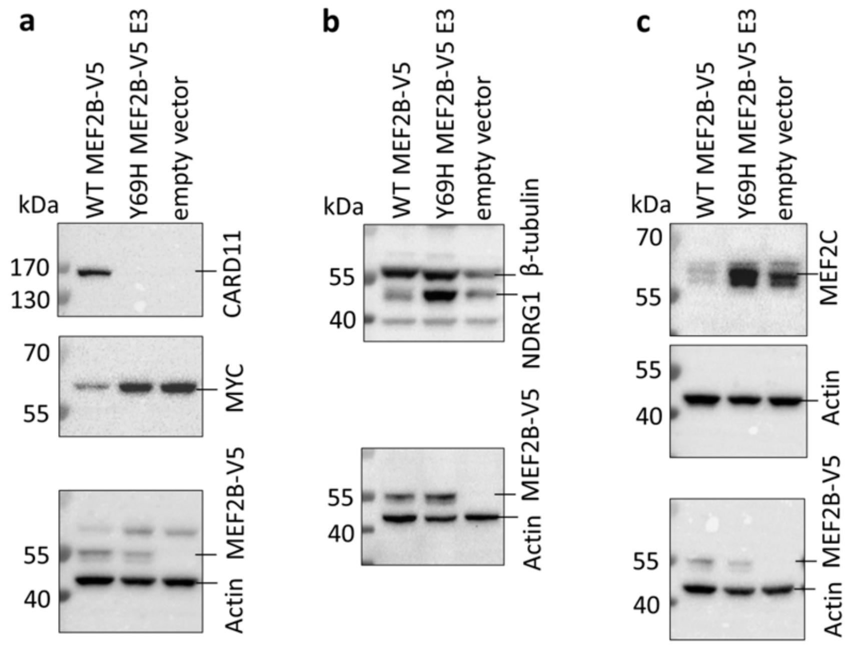

Supplementary Figure 22: Validation of differential protein abundance in an additional Y69H MEF2B-V5 expressing HEK293A cell line

Shown are western blots for **(a)** CARD11 and MYC, **(b)** NDRG1 and **(c)** MEF2C protein abundance. **(a)** CARD11, MYC and MEF2B-V5 were probed on the same membrane, and are thus shown in the same panel. **(b,c)** MEF2B-V5 was detected in the same lysates as were probed for NDRG1 and MEF2C. For all panels, MEF2B-V5 was detected using V5 antibody. The Y69H MEF2B-V5 E3 cells are a different monoclonal cell line than the Y69H MEF2B-V5 cells that were used for microarrays and RNA-seq. The WT MEF2B-V5 cells are the monoclonal cell line used for microarrays and RNA-seq. Empty vector cells were stably transfected with empty pcDNA3 vector.

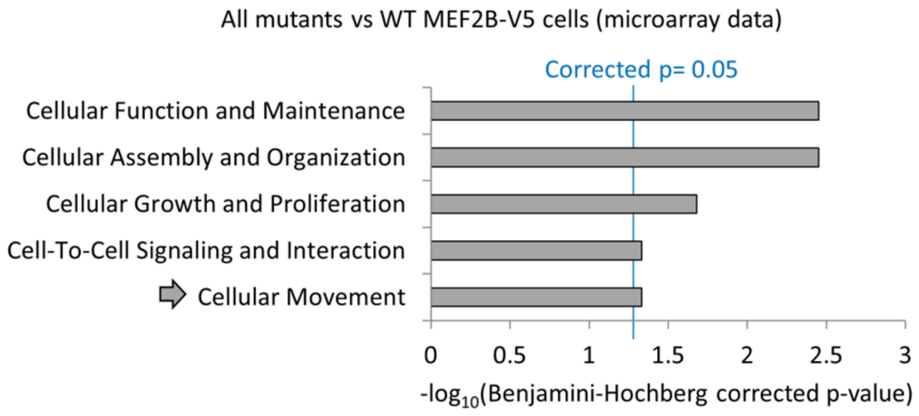

Supplementary Figure 23: Cellular function annotation categories enriched in genes differentially expressed in K4E versus WT, Y69H versus WT, and D83V versus WT MEF2B-V5 cells.

Enrichment analysis considered only genes that were differentially expressed at a B-H adjusted eBayes p-value < 0.05 and had the same direction of expression change in all three comparisons. Shown are B-H adjusted right-tailed Fisher exact test p-values for enrichment, calculated using Ingenuity Pathway Analysis. The arrow indicates a category discussed in the main text.

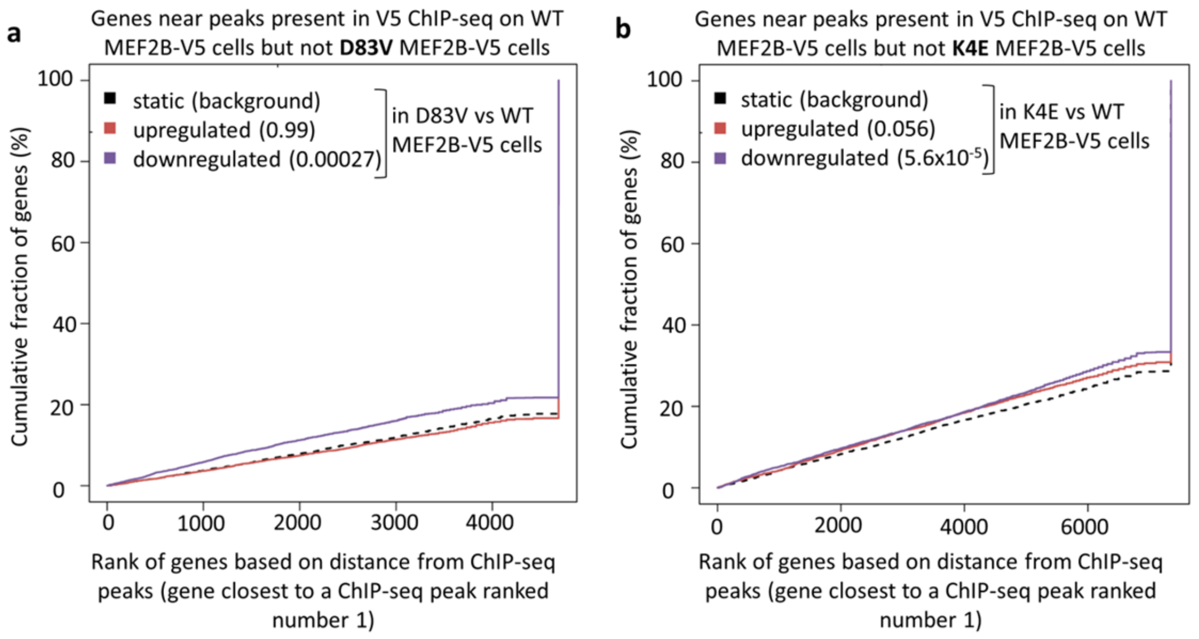

Supplementary Figure 24: Genes with decreased expression in mutant versus WT MEF2B-V5 cells tend to be the closest to peaks that are present WT but not mutant ChIP-seq

Rank numbers are shown on the *x*-axis. Lower ranks indicate shorter distances between the genes' TSSs and ChIP-seq peaks. The *y*-axis indicates the proportion of genes with ranks at or better than the *x*-axis value. Rankings were calculated and plotted using BETA[11]. P-values are compared to the background distribution (one-tailed Kolmogorov-Smirnov test). Analyses considered only peaks that were present in both replicates of WT ChIP-seq but neither replicate of **(a)** D83V or **(b)** K4E mutant ChIP-seq. The direction of gene expression change was obtained from expression microarray data, using a threshold for differential expression of 0.05 (B-H adjusted eBayes p-value).

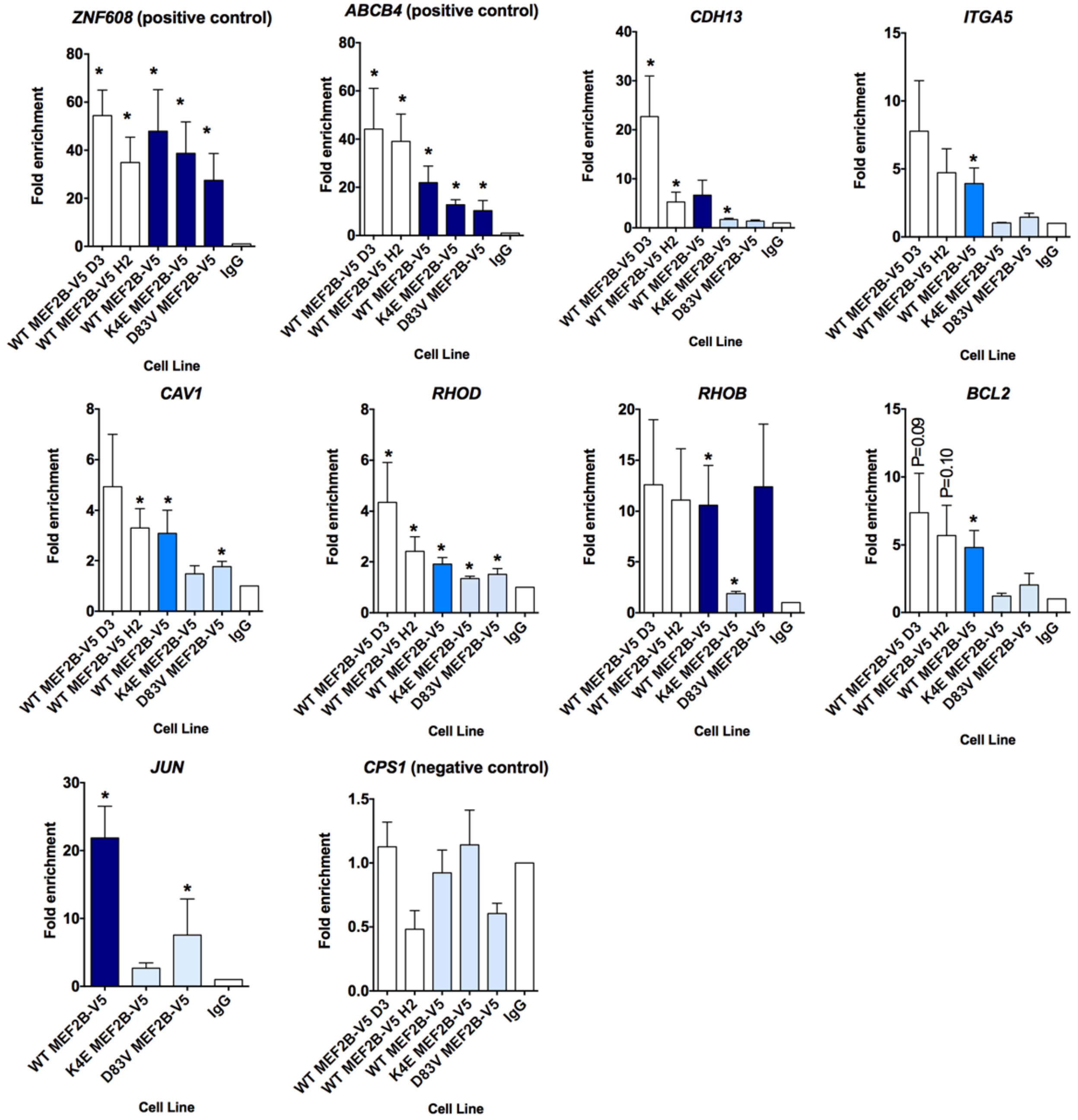

Supplementary Figure 25: ChIP-qPCR data produced for verification of ChIP-seq on mutant and WT MEF2B-V5 cells

Shown are the mean fold enrichments of DNA regions in three biological replicates of ChIP-qPCR. V5 ChIP-qPCR on K4E and D83V MEF2B-V5 cells tended to produce lower fold enrichments than V5 ChIP-qPCR on WT MEF2B-V5 cells. Note that *y*-axis scales differ between plots. Plot titles indicate genes whose TSS is within 5 kb up or downstream of the DNA region assessed. All ChIPs used V5 antibody except the ‘IgG’ sample, which used normal mouse immunoglobulin on chromatin from WT MEF2B-V5 cells. Fold enrichment was calculated compared to enrichment of an intergenic DNA region not expected to interact with MEF2B, then

normalized to fold enrichment in ChIP-qPCR using normal immunoglobulin. The K4E, D83V and WT MEF2B-V5 cell lines were the monoclonal cell lines used for ChIP-seq. WT MEF2B-V5 D3 and H2 were monoclonal cell lines different from the cell lines used for ChIP-seq. All MEF2B-V5 cell lines were HEK293A cells stably transfected with MEF2B-V5. Bar shading indicates how many replicates of ChIP-seq on the cell line indicated by the bar had a peak (FDR 0.05 using MACS2[5]) in that region (light blue: no replicates; medium blue: 1 replicate; dark blue: both replicates). Error bars represent the s.e.m. * P < 0.05 (Student's two tailed *t*-test, unpaired) compared to ChIP using normal immunoglobulin.

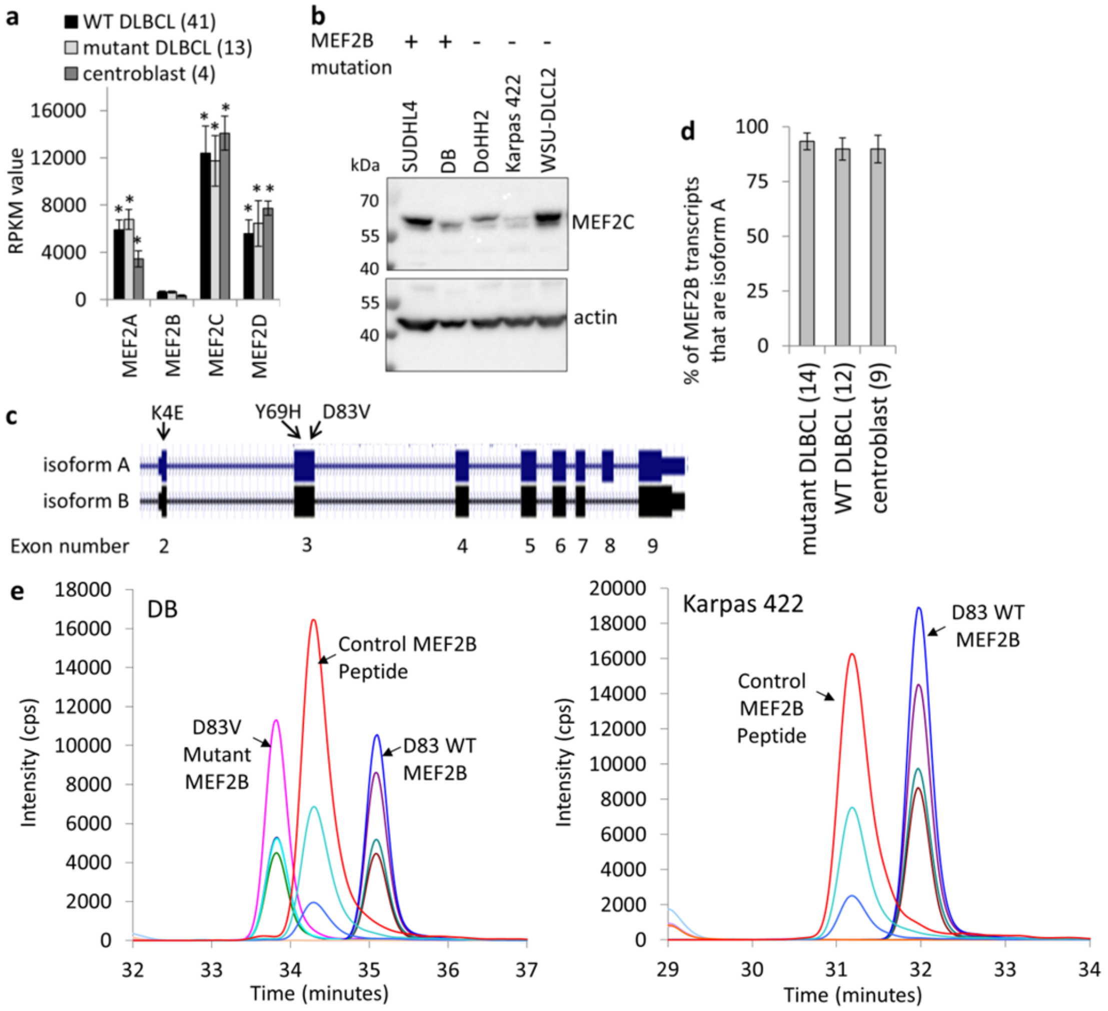

Supplementary Figure 26: Expression of MEF2 family members in DLBCL cells

(a) *MEF2A*, *C* and *D* mRNA expression is greater than *MEF2B* mRNA expression in RNA-seq data for DLBCL patient samples and normal centroblasts. RPKM values are the number of mapped reads per length of transcript in kilobases per million mapped reads. * P < 0.05 compared to MEF2B expression in the same cell type (Student's two tailed *t*-test, unpaired). In brackets are the numbers of samples assessed. Error bars represent the s.e.m. **(b)** Western blot of MEF2C in DLBCL cell lines. MEF2C expression did not correlate with *MEF2B* mutation status.

(c) Isoform B MEF2B skips exon 8, resulting in a frameshift that causes the amino acid sequence of isoform B to differ from isoform A by 30%. The transcript structure was obtained from the UCSC Genome Browser (hg19)[10]. **(d)** The majority of *MEF2B* transcripts in DLBCL RNA-seq data were isoform A. In brackets are the numbers of samples scored within each group. Error bars represent the s.d. For **(a)** and **(d)** 'mutant' and 'WT' indicate *MEF2B* mutation status. **(e)** Both D83 and D83V mutant peptides were detected using multiple reaction monitoring mass spectrometry in DLBCL cells with the D83V *MEF2B* mutation (DB), indicating that both mutant and WT *MEF2B* were expressed. D83V peptide was not expressed in DLBCL cells without *MEF2B* mutation (Karpas 422), confirming the specificity of D83V peptide detection.

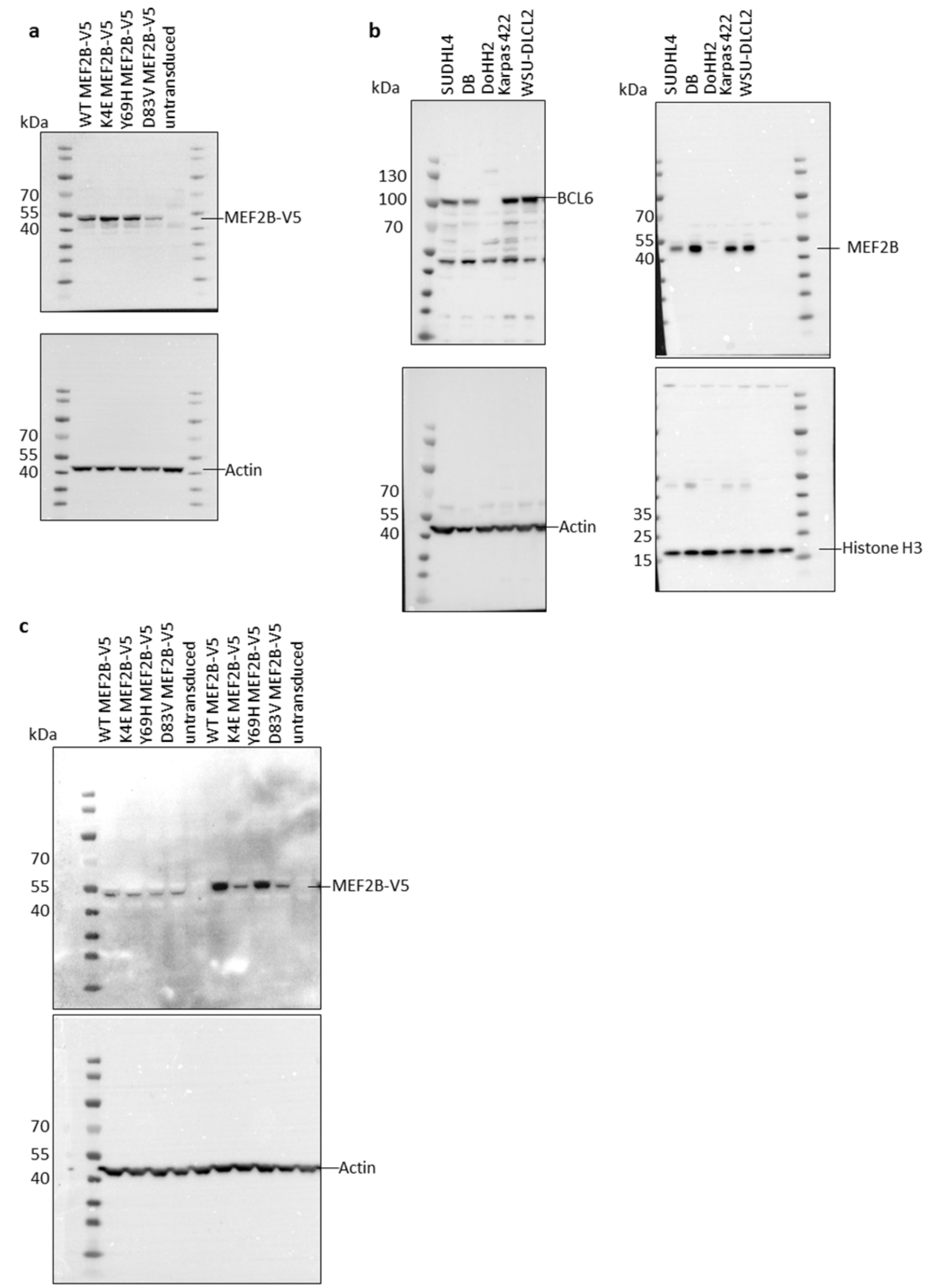

Supplementary Figure 27: Full western blots shown in Figure 6

Portions of the western blots shown in **(a)** and **(b)** are shown in Figure 6 (a) and (b), respectively. Portions of the western blots shown in **(c)** are shown in Figure 6c.

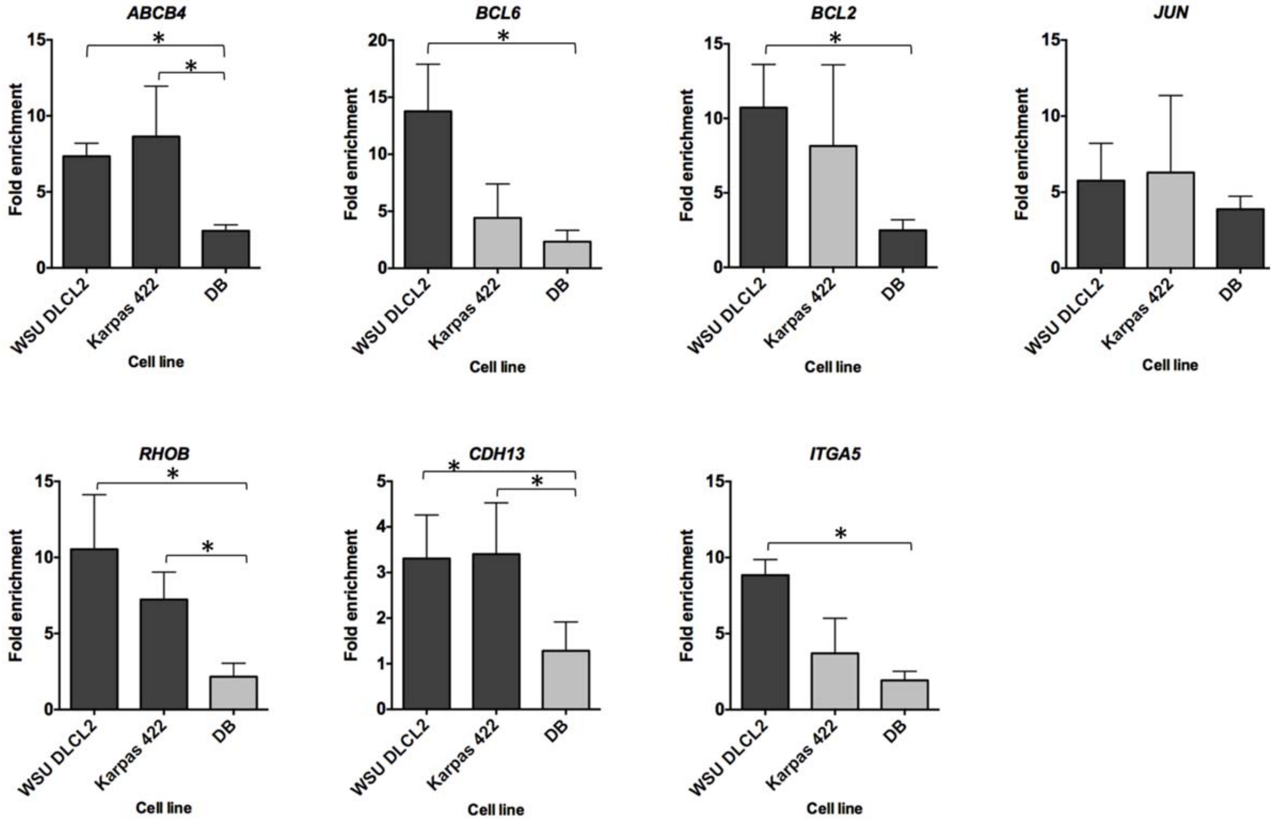

Supplementary Figure 28: ChIP-qPCR on DLBCL cells identifies DNA regions bound by MEF2B

ChIP-qPCR on DLBCL cells with no *MEF2B* mutations (i.e. Karpas 422 and WSU-DLCL2) tended to produce greater fold enrichments than ChIP-qPCR on DLBCL cells with an endogenous D83V *MEF2B* mutation (DB). Shown are mean fold enrichments in ChIP-qPCR using MEF2B antibody (ProSci) compared to ChIP-qPCR using normal rabbit immunoglobulin (IgG) on the same chromatin. Error bars represent the s.e.m. of three biological replicates. Note that *y*-axis scales differ between plots. * $P < 0.05$ compared to DB cells. Dark grey bars indicate $P < 0.05$ compared to the IgG ChIP. All statistical testing used an unpaired Student's two tailed *t*-test.

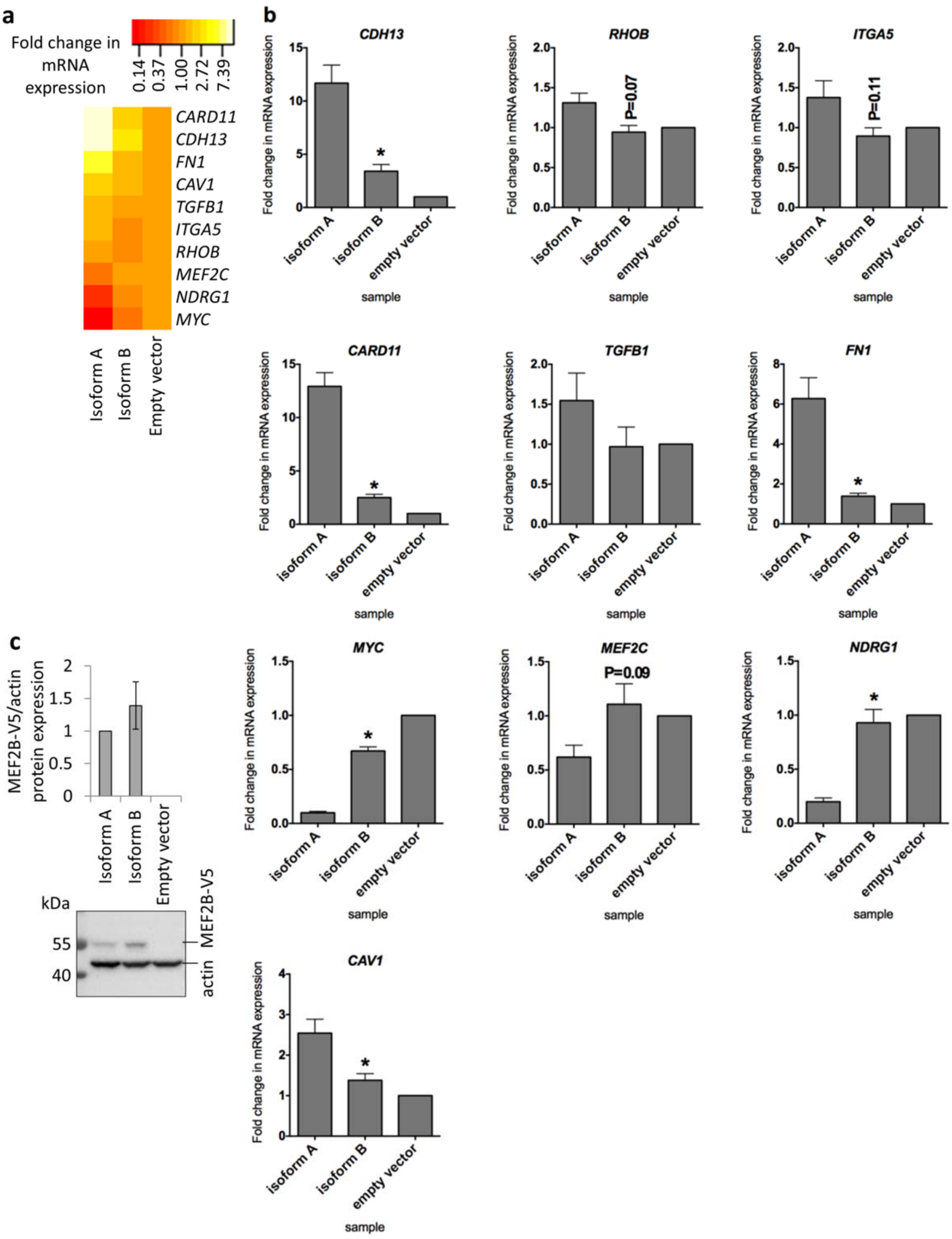
a
Fold change in mRNA expression
0.14
0.37
1.00
2.72
7.39
CARD11
CDH13
FN1
CAV1
TGFB1
ITGA5
RHOB
MEF2C
NDRG1
MYC
Isoform A
Isoform B
Empty vector
b
CDH13
RHOB
ITGA5
CARD11
TGFB1
FN1
MYC
MEF2C
NDRG1
CAV1
P=0.07
P=0.11
P=0.09
Fold change in mRNA expression
isoform A
isoform B
empty vector
sample
c
MEF2B-V5/actin protein expression
Isoform A
Isoform B
Empty vector
kDa
55
40
MEF2B-V5
actin

Supplementary Figure 29: Expression of isoform B MEF2B-V5 affects MEF2B target gene expression less than expression of isoform A MEF2B-V5

Data are shown as **(a)** a heatmap and **(b)** bar plots. Both panels show mean fold changes in RNA expression compared to empty vector control cells over three biological replicates. Data were produced using qRT-PCR and were normalized to *PGK1* expression. Note that *y*-axis scales differ between plots. Error bars represent the s.e.m. of three biological replicates. * $P < 0.05$ in comparison to isoform A MEF2B-V5 expressing cells (Student's two tailed *t*-test, unpaired). **(c)** MEF2B-V5 protein abundance is greater in the isoform B cell line than in the isoform A cell line. Error bars indicate the s.e.m. of three biological replicates. A representative western blot is shown.

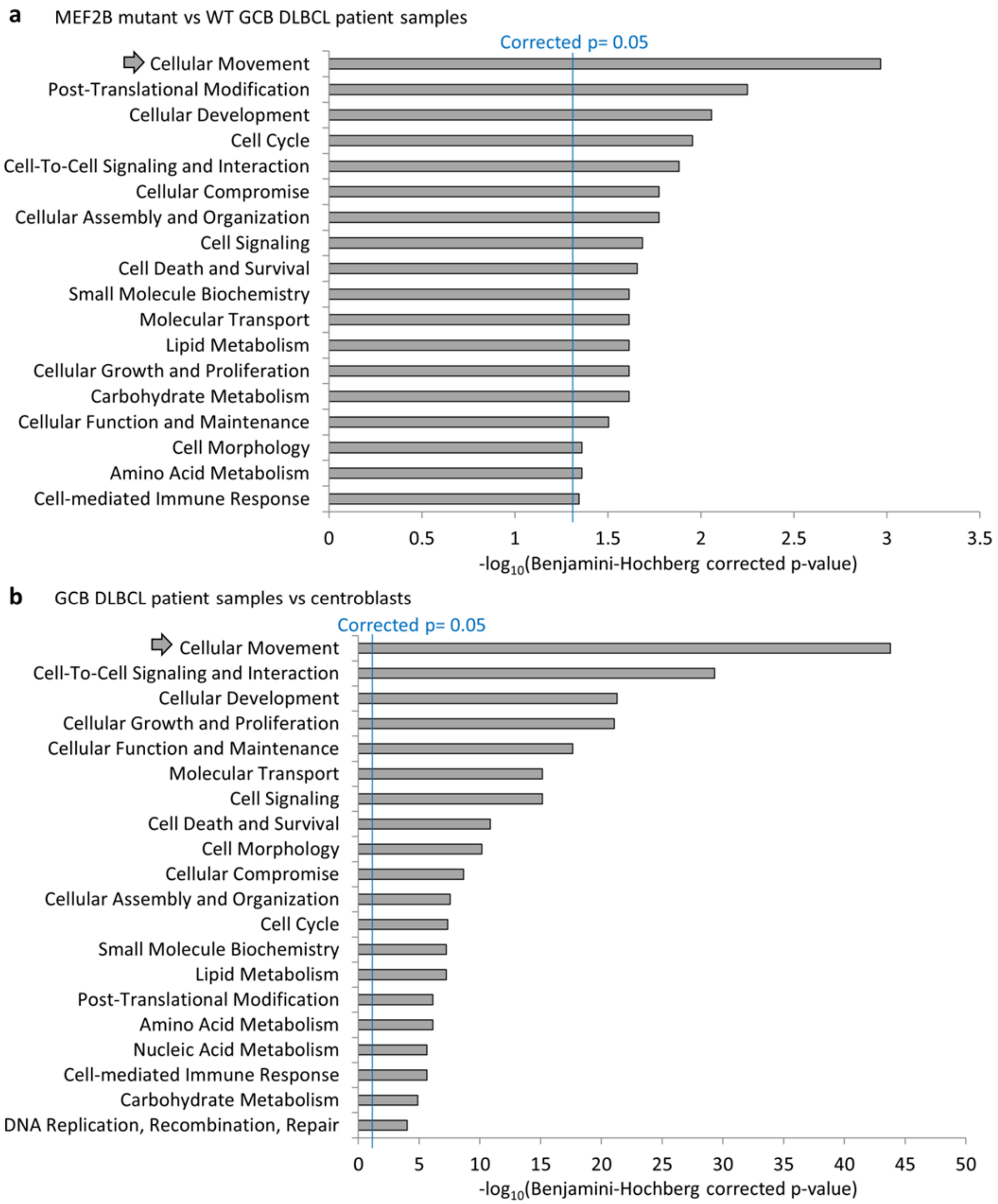

Supplementary Figure 30: Cellular function annotation categories enriched in genes differentially expressed between DLBCL patient samples

Annotation groups relating to cellular movement (indicated by the arrow) were highly enriched for in both **(a)** the genes differentially expressed between GCB DLBCL patient samples with and without *MEF2B* mutations (B-H adjusted DEseq[1] p-values < 0.1) and **(b)** the genes differentially expressed between GCB DLBCL patient samples and centroblasts (B-H adjusted DEseq[1] p-values < 0.05). Shown are B-H adjusted right-tailed Fisher exact test p-values for enrichment, calculated using Ingenuity Pathway Analysis.

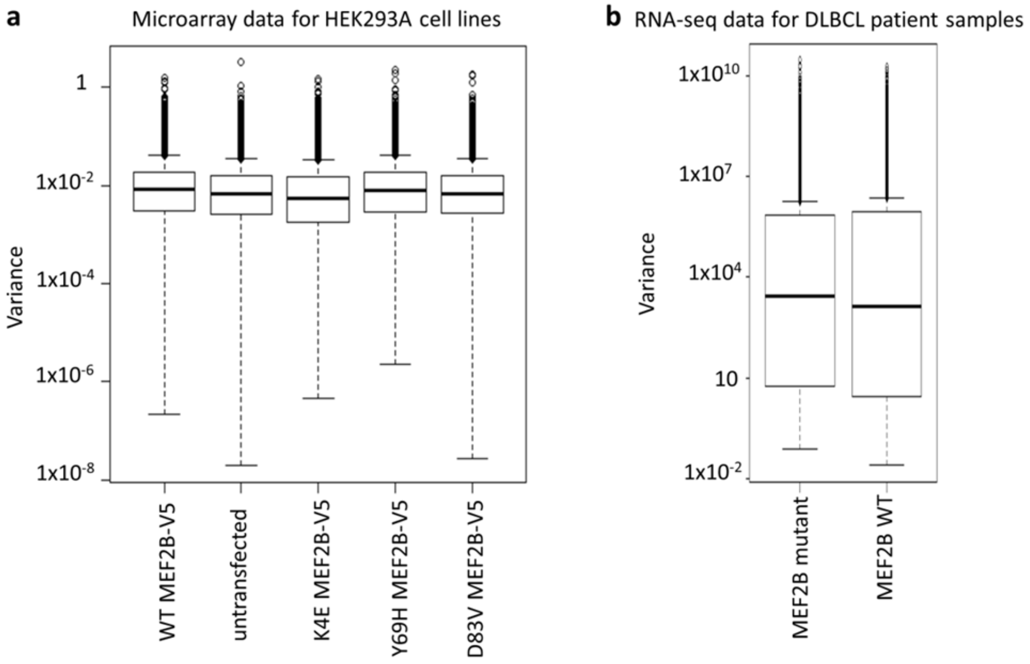

Supplementary Figure 31: Groups used for statistical comparisons showed similar variance in gene expression values

For both panels, the distribution of variance values across all genes is shown. The variance in expression values was calculated for each gene **(a)** across three biological replicates of microarray data and **(b)** across GCB DLBCL patient samples that had mutant MEF2B (13 samples) or WT MEF2B (40 samples). Variance was determined using the 'var' function in R 2.14.1 and is shown on a log scale in Tukey boxplots.

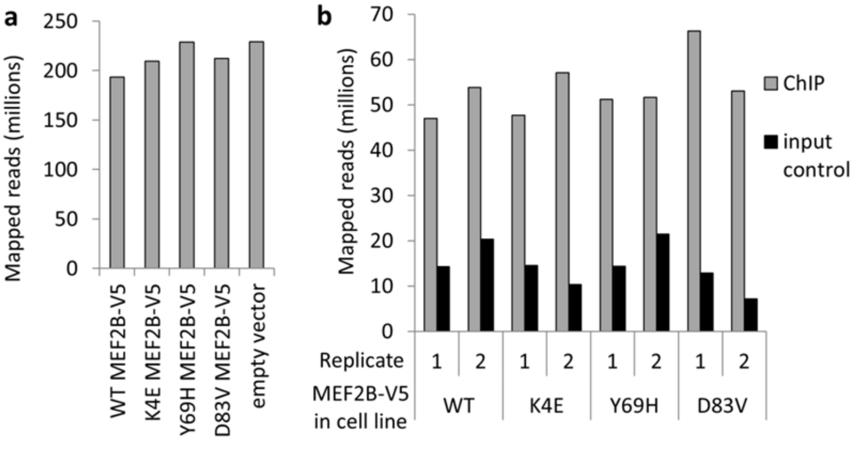

Supplementary Figure 32: Number of mapped reads in RNA-seq and ChIP-seq datasets

Similar numbers of mapped reads were produced between cell types for **(a)** RNA-seq samples and **(b)** ChIP-seq and input DNA control samples.

Gene Symbol	microarray data<br>WT MEF2B-V5 vs untransfected cells	RNA-seq data<br>WT MEF2B-V5 vs empty vector cells	qRT-PCR on validation set lines<br>WT MEF2B-V5 D3 vs empty vector cells	 WT MEF2B-V5 H2 vs empty vector cells
AKT1	up			up
AMOT	up	up	up	up
BCL6	up		up	up
CARD11	up	up	up	up
CAV1	up	up		up
CCL2	up	up	up	up
CDH13	up	up	up	up
CTSB	up	up	up	up
CXCL12	up			up
FN1	up	up	up	up
GNA12	up		up	up
INPP5K	down	down		
ITGA5	up		up	up
LGALS1	up	up	up	up
MEF2C	down		down	down
MYC	down	down	down	down
NDRG1	down	down	down	down
PAK1	up		up	up
PLCG1	up	up	up	up
RHOB	up			up
RHOD	up	up	up	up
ROCK1	up			up
RRAS	up	up	up	up
SEMA3C	up	up		
SIX1	down		up	up
SMAD2	up			up
SMAD3	up		up	
SMAD4	up			up
TGFB1	up		up	up
VEGFB	up			up
**total significant**	**30**	**15**	**20**	**27**
**matches to microarray data**		**15**	**19**	**26**

Supplementary Table 1: Differential expression of the validation set genes in WT MEF2B-V5 versus control cells according to microarray, RNA-seq and qRT-PCR data

The direction of expression change is indicated for differentially expressed genes (Student's t-test p-values < 0.05 for qRT-PCR; DEseq[1] p-values < 0.05 for RNA-seq; B-H adjusted eBayes p-values < 0.05 for microarray). In yellow are cases in which the direction of change in RNA-seq or qRT-PCR matches the direction of change in microarray data. WT MEF2B-V5 D3 and H2 were different monoclonal lines than the WT MEF2B-V5 line used for microarrays and RNA-seq.

Category	Functions Annotation	p-value	Activation z-score	# of genes
Cellular Movement	migration of cells	2.18E-02	2.7	44
Cellular Movement	cell movement	1.06E-02	2.55	50
Cellular Movement	migration of tumor cell lines	1.93E-02	2.103	30

Supplementary Table 2: Cellular function annotation groups enriched in genes differentially expressed in RNA-seq data for WT MEF2B-V5 versus empty vector cells

IPA cellular function annotation groups with absolute activation z-scores of at least 2 and B-H adjusted right-tailed Fisher exact test p-values less than 0.05 for the genes differentially expressed in RNA-seq data for WT MEF2B-V5 versus empty vector HEK293A cells. Positive z-scores indicate MEF2B activity promotes the function and negative z-scores indicate MEF2B activity opposes the function.

MEF2B-V5 in cells	Sample type	Replicate	Total number of reads	% of total reads that are:		Number of peaks (FDR 0.05 using MACS2[5])	Number of reads in peaks (N)	N as % of total mapped reads
				Mapped reads	Uniquely mapped reads			
WT	V5 ChIP	1	54051882	93.0	89.1	5599	1175808	2.34
		2	61453292	91.3	87.7	19642	4623691	8.24
	input control	1	37019950	41.9	38.7		11929	0.03
		2	38922762	55.0	52.3		83393	0.21
K4E	V5 ChIP	1	53218136	93.8	89.7	175	39119	0.08
		2	63408292	94.5	90.1	38	13325	0.02
	input control	1	38482650	40.6	37.8		431	0.001
		2	36745420	29.8	28.1		177	0.0005
D83V	V5 ChIP	1	74619022	92.6	88.9	3771	884037	1.28
		2	60810194	91.3	87.3	427	114273	0.21
	input control	1	37621370	37.7	34.4		4842	0.01
		2	31686572	24.2	22.8		368	0.001

Supplementary Table 3: Quality control statistics for ChIP-seq data

The ‘number of reads in peaks’ for the input control samples was calculated using the peak regions from the matched ChIP-seq sample. Peaks in ChIP-seq samples were identified compared to the read distribution in input control samples using MACS2[5].

Rank	Motif	p-value	% of ChIP-seq peak regions containing the motif	% of background sequences containing the motif	Best match to a known motif
1	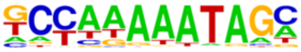	1E-1274	44.23%	7.01%	Mef2c(MADS)/GM12878-Mef2c-ChIP-Seq(GSE32465)/Homer
2	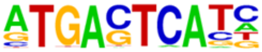	1E-792	23.65%	2.69%	AP-1(bZIP)/ThioMac-PU.1-ChIP-Seq(GSE21512)/Homer
3	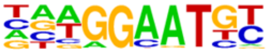	1E-236	31.84%	14.38%	TEAD4(TEA)/Tropoblast-Tead4-ChIP-Seq(GSE37350)/Homer
4	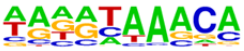	1E-181	22.39%	9.35%	MF0005.1_Forkhead_class/Jaspar
5	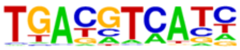	1E-165	13.44%	4.22%	Atf1(bZIP)/K562-ATF1-ChIP-Seq(GSE31477)/Homer
6	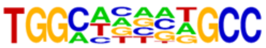	1E-102	9.24%	3.07%	NF1(CTF)/LNCAP-NF1-ChIP-Seq/Homer
7	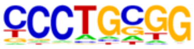	1E-102	32.37%	20.05%	AP-2alpha(AP2)/Hela-AP2alpha-ChIP-Seq/Homer
8	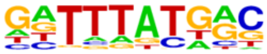	1E-81	9.44%	3.66%	Cdx2(Homeobox)/mES-Cdx2-ChIP-Seq/Homer
9	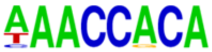	1E-81	8.86%	3.31%	RUNX1(Runt)/Jurkat-RUNX1-ChIP-Seq/Homer
10	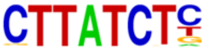	1E-79	9.76%	3.93%	Gata2(Zf)/K562-GATA2-ChIP-Seq/Homer

Supplementary Table 4: Motifs identified *de novo* in WT MEF2B-V5 ChIP-seq peak regions

De novo motifs were identified using the ChIP-seek[12] implementation of HOMER on sequences within 100 bp of the centres of MEF2B-V5 ChIP-seq peaks. Only peaks identified in both replicates of ChIP-seq at a MACS2[5] FDR of 0.05 were included for analysis. The top ten most enriched motifs are shown.

Category	Functions Annotation	p-value	Activation z-score	# of genes
Cell Cycle	S phase of tumor cell lines	7.66E-03	2.433	7
Cellular Movement	cell movement of cancer cells	9.90E-04	-2	6

Supplementary Table 5: Cellular function annotation groups enriched in genes differentially expressed in K4E versus WT, Y69H versus WT, and D83V versus WT MEF2B-V5 cells.

IPA cellular function annotation groups with absolute activation z-scores of at least 2 and B-H adjusted right-tailed Fisher exact test p-values less than 0.05, for the genes differentially expressed in all mutant versus WT MEF2B-V5 HEK293A cells. Positive z-scores indicate increased activity in mutant versus WT cells and negative z-scores indicate decreased activity in mutant versus WT cells.

	Peaks in both replicates of WT MEF2B-V5 ChIP-seq but neither replicate of K4E MEF2B-V5 ChIP-seq			Peaks in both replicates of WT MEF2B-V5 ChIP-seq but neither replicate of D83V MEF2B-V5 ChIP-seq		
motif	p-value for enrichment	p-value rank (most significant =1)	% of peak regions containing the motif	p-value for enrichment	p-value rank (most significant =1)	% of peak regions containing the motif
MEF2A	1e-715	1	34.6%	1.00E-283	3	29.5%
MEF2C	1e-680	2	36.5%	1.00E-283	4	32.0%
JUN-AP1	1e-661	3	18.5%	1e-385	1	19.6%
AP1	1e-589	4	30.6%	1e-344	2	32.2%

Supplementary Table 6: Motifs enriched in regions with peaks in WT but not mutant MEF2B-V5 ChIP-seq

Motifs were identified using the ChIPseek[12] implementation of HOMER. Peaks were identified over input control DNA at a FDR of 0.05 using MACS2[5].

motif	Peaks in both replicates of V5 ChIP-seq on K4E MEF2B-V5 cells			Peaks in both replicates of V5 ChIP-seq on D83V MEF2B-V5 cells		
	p-value for enrichment	p-value rank (most significant =1)	% of peak regions containing the motif	p-value for enrichment	p-value rank (most significant =1)	% of peak regions containing the motif
MEF2A	1.00E-20	1	72.2%	1.00E-104	1	47.3%
MEF2C	1.00E-19	2	75.0%	1.00E-96	2	48.7%
JUN-AP1	1.00E-01	25	8.3%	1.00E-40	3	15.4%
AP1	1.00E-03	6	25.0%	1.00E-37	4	26.7%

Supplementary Table 7: Motifs enriched in regions with peaks in mutant MEF2B-V5 ChIP-seq

Motifs were identified using the ChIPseek[12] implementation of HOMER. Peaks were identified over input control DNA at a FDR of 0.05 using MACS2[5].

Category	Functions Annotation	p-value	Activation z-score	# of genes
Cellular Movement	chemotaxis of cells	6.01E-03	2.545	14
Cellular Movement	chemotaxis of leukocytes	3.18E-04	2.319	13
Cell Signaling, Molecular Transport	quantity of Ca2+	5.07E-03	2.286	11
Cell-To-Cell Signaling and Interaction	activation of tumor cell lines	6.39E-04	2.213	7
Cell-To-Cell Signaling and Interaction	adhesion of phagocytes	7.20E-03	2.193	5
Cellular Movement	chemotaxis of neutrophils	1.65E-05	2.173	9
Cellular Function and Maintenance	ion homeostasis of cells	1.22E-02	2.17	11
Cell Signaling	mobilization of Ca2+	2.04E-03	2.158	12
Cellular Movement	chemotaxis of phagocytes	6.73E-05	2.125	12
Cell Death and Survival	cell viability of mononuclear leukocytes	1.34E-02	2	4
Cellular Movement	chemotaxis of leukemia cell lines	7.20E-03	2	5

Supplementary Table 8: Cellular function annotation groups enriched in genes differentially expressed in DLBCL patient samples with versus without *MEF2B* mutations

IPA cellular function annotation groups with absolute activation z-scores of at least 2 and B-H adjusted right-tailed Fisher exact test p-values less than 0.05 for genes differentially expressed in GCB DLBCL patient samples with versus without *MEF2B* mutations. Positive z-scores indicate increased activity in MEF2B mutant versus WT samples and negative z-scores indicate decreased activity in MEF2B mutant versus WT samples. In blue are annotation groups related to cell migration.

	HEK293A				DLBCL
Gene Symbol	untransfected cells vs WT MEF2B-V5 cells	K4E vs WT MEF2B-V5 cells	Y69H vs WT MEF2B-V5 cells	D83V vs WT MEF2B-V5 cells	mutant vs WT
AMHR2	up			up	up
ATOH1	down				down
BMP5	down		down	down	down
CGA	down	up			down
CNTFR	down	down	down		up
COL2A1	up	up		up	up
CRYM	up				up
DHRS2	up		up		up
DPPA2	down		down		down
DPYSL5	down	down		down	up
DSC3	down		down	down	down
EEF1A2	down		down		down
EPHA7	up	up	up		up
ESRP1	down		down	down	down
GLT1D1	down				down
IGSF1	down				down
LIN28B	up			up	down
LRRTM3	up				down
MATN3	down				up
MC4R	up	up			up
NBPF4	up				down
NR5A1	up				up
NTF3	down	down	down	down	up
OR51B4	up			up	up
RGS7	up			up	up
RIMS1	down				down
SFN	down		down		down
SLITRK1	up				down
SLITRK5	up		up	up	down
TFPI2	down		down		down

Supplementary Table 9: Differentially expressed genes in DLBCL and HEK293A cells with versus without mutant *MEF2B*

Listed are all genes that were differentially expressed in DLBCL cells with versus without *MEF2B* mutations (B-H adjusted DEseq[1] p-values < 0.05) and untransfected versus WT MEF2B-V5 HEK293A cells (B-H adjusted eBayes p-values < 0.05). Directions of expression change in other comparisons of HEK293A cells are indicated where the change was identified at a B-H adjusted eBayes p-value < 0.05.

**Cell line**	**Source**	**Date of authentication**	**Authentication performed by**	**Date of sample collection for *Mycoplasma* testing**	**Results of *Mycoplasma* testing**
HEK-293A	Dr. Gregg Morin	Aug 2014	Genetica DNA Laboratories	Mar 16th 2014	Negative
SUDHL4	DSMZ (ACC-495)	May 2009	DSMZ	Oct 14th 2013	Negative
DoHH2	DSMZ (ACC-47)	Sept 2008	DSMZ	Mar 1st 2014	Negative
DB	DSMZ (ACC-539)	Sept 2008	DSMZ	Oct 24th 2013	Negative
Karpas-422	DSMZ	Sept 2008	DSMZ	Oct 22nd 2013	Negative
WSU-DLCL2	DSMZ (ACC-575)	Sept 2008	DSMZ	Aug 17th 2014	Negative

Supplementary Table 10: Source, authentication and *Mycoplasma* testing of cell lines

**Assay**	**Gene**	**Forward primer (5' to 3')**	**Reverse primer (5' to 3')**
qRT-PCR	*MEF2A*	CAAATGGAGCTGGAAGCAGT	GGAGGGGGAGACTTTGTAGG
	MEF2B	GACCGTGTGCTGCTGAAGTA	AGCCTCCGAAACTTCTCTCC
	MEF2C	ACCAGGTGAGACCAGCAGAC	GTGGTCTGATGGGTGGAGAC
	MEF2D	GTTGAAGCCCTTCTTCCTCA	TCAACCACTCCAACAAGCTG
	AKT1	CCCAGCAGCTTCAGGTACTC	GCTCACCCAGTGACAACTCA
	AMOT	GCCTCTCTTTTGGAGGATGA	GACGAGAACCGGAACTTGAG
	BCL6	TGAGAAGCCCTATCCCTGTG	CTGGCTTTTGTGACGGAAAT
	CARD11	TCCAACATCTACCCCATCGT	CAGGAACTCCTCCTCCGTCT
	CAV1	GAGCTGAGCGAGAAGCAAGT	TCCCTTCTGGTTCTGCAATC
	CCL2	CCCCAGTCACCTGCTGTTAT	AGATCTCCTTGGCCACAATG
	CDH13	GAATGACAACCGACCGATCT	TATACCGCAGGAGGGCATTA
	CTSB	CCAGTAGGGTGTGCCATTCT	TGTGTATTCGGACTTCCTGCT
	CXCL12	CTTTAGCTTCGGGTCAATGC	AGAGCCAACGTCAAGCATCT
	FN1	GCTCATCATCTGGCCATTTT	TGCTTAGGCTTTGGAAGTGG
	GNA12	GGTCCAAGTTGTCCAGGAAG	CCACCTTCCAGCTGTACGTC
	INPP5K	GCTTCAGCCTCCACAGGAT	CGCCCACCTACAAGTTTGAT
	ITGA5	CTGTTCCCCTGAGAAGTTGTAGA	GTGCCCAAAGGGAACCTC
	JUN	GTTGCAGTGGAGAGGGACAG	CCACCAATTCCTGCTTTGAG

Assay	Gene	Forward primer (5' to 3')	Reverse primer (5' to 3')
qRT-PCR (continued)	*LGALS1*	CAAACCTGGAGAGTGCCTTC	CAGGTTGTTGCTGTCTTTGC
	MAP2	GAGAATGGGATCAACGGAGA	CTGCTACAGCCTCAGCAGTG
	MYC	GAGGCTATTCTGCCCATTTG	CACCGAGTCGTAGTCGAGGT
	NDRG1	GGGTGCCATCCAGAGAAGT	CTCGCTGAGGCCTTCAAGTA
	PAK1	TCCCTCATGACCAGGATCTC	ACCGTGTACACAGCAATGGA
	PLCG1	TACCATGGGCACACCCTTAC	AGTGGTCCTCAATGGACAGG
	RHOB	CGACGTCATTCTCATGTGCT	GGGACAGAAGTGCTTCACCT
	RHOD	GTCGCTGCTGATGGTCTTC	GCACAGGTTTGCCTTTCACT
	ROCK1	TTTGAGATGCTTCACCTCCTC	GCTGAACGAAGAGACAGAGGTC
	RRAS	TCCTCAATAGTGGGGTCGTAG	CAGCGAGACACACAAGCTG
	SEMA3C	CTTCCAGCTCCTCCAGAATG	ACGCTGCTGATGGGAGATAC
	SIX1	CCCCTTCCAGAGGAGAGAGT	TTAAGAACCGGAGGCAAAGA
	SMAD2	GGCCTGTTGTATCCCACTGA	TGAGCTTGAGAAAGCCATCA
	SMAD3	CAGGGCTTTGAGGCTGTCTA	AGCAGGGGGTACTGGTCAC
	SMAD4	TGGAGCTCATCCTAGTAAATGTGT	TTGTGAAGATCAGGCCACCT
	TGFB1	CCGGGTTATGCTGGTTGTA	GGCTACCATGCCAACTTCTG
	VEGFB	CGGTACCCGAGCAGTCAG	GGCTTCACAGCACTGTCCTT
	PGK1	GGGAAAAGATGCTTCTGGGAA	TTGGAAAGTGAAGCTCGGAAA
	TBP	CAGCTCTTCCACTCACAGACT	GTGCAATGGTCTTTAGGTCAA
ChIP-qPCR	*RHOB*	GCGGCCAATCAGAGCTAAG	CCTAGCGCCCGCTATTTA
	CAV1	GAGATGATGCACTGCGAAAA	GGTGCTTGGGCAGATTATTT
	CDH13	GGCATTTTGGTAGGAGGTGA	GCCACTTCTGGGACAGACTT
	ITGA5	TGTCTGACCCAGGAGAAACC	TCCTGTGCTCTGTGCAAACT
	PAK1	GCACATAGCTGCTGGAGTCA	GGTGAGGAAACCTGAGAGTCA
	RHOD	ACGCCTGGATCCAAATTCTA	GGCACTGTTCCCTAGGAGGT
	BCL2	TGTGGTGTGCTTCTTGACATC	TGCAATTCAGTGCTTCCTTTT
	JUN	GGGTGACATCATGGGCTATT	CTGTCTGTCTGCCTGACTCC
	ZNF608	AGGGAGTGGCGACTTTTACA	TGATAAATTGGACCTTATGAAACCT
	ABCB4	TCAGGCTAAAGGCGAAAATG	CCTGGCCCTTGTTAAACTCA
	BCL6	TGCATTGTAGTTGTGGCAGTC	GAGCCAAATACCTGTTTGTGTTT
	CPS1	TGGAGAAAAGTTTATTCTAACGTTCTT	TGTTGGGGTAAAAAGACAATGTT
	intergenic	TATTTGATGGTCCCCAAACC	AGGATGGTCCAGGGAAAGAC
PCR of MEF2B cDNA		GGAGGAATTCATGGGGAGGAAAAAAATCCAGATC	GGAGGCTAGCCCGGGGCCAGCCGTCGGCCA
Sequencing MEF2B cDNA	A1R	AGTCAGCATCAAGTCTGAGCG	
	A2F	CGCTCAGACTTGATGCTGACT	
	A3R	AAGCCACCTCACCAGCAAGAC	
	A4F	GTCTTGCTGGTGAGGTGGCTT	
	A5.2	AGGAGCCAGGAGAGAAGTTT	

Assay	Gene	Forward primer (5' to 3')	Reverse primer (5' to 3')
	A7	GGGGAGCTTCCCCTTCCTC	
Site-directed mutagenesis	K4E	TGCGGAAGATCTGGATTTTTTCCCTCCCCATCG	CGATGGGGAGGGAAAAAATCCAGATCTCCCGCA
	Y69H	CGCTGTACTCTGTGTGCTTCAGCAGCACACG	CGTGTGCTGCTGAAGCACACAGAGTACAGCG
	D83V	CCGCACCAACACTGTCATCCTCGAGACGC	GCGTCTCGAGGATGACAGTGTTGGTGCGG
	R3T	GAGATCTGGATTTTTTTCGTCCCCATGGTTCGAACTC	GAGTTCGAACCATGGGGACGAAAAAAATCCAGATCTC
	R24L	GTGACGTTCACCAAGCTGAAGTTCGGGCTGATG	CATCAGCCCGAACTTCAGCTTGGTG AACGTCAC
EMSA probes	*JUN*	GCCAGTCAACCCCTAAAAATAGCCCATGAT	GGGTGACATCATGGGCTATTTTTAGGGGTT
	BCL2	GTCCCAAGAGGCTATAAAAGGAAGC	GATTCAGTGCTTCCTTTTATAGCCTC

Supplementary Table 11: Sequences of PCR and sequencing primers, gel-shift assay probes and site-directed mutagenesis oligonucleotides

sample	total reads	duplicate reads	mapped reads (M)	M as % of total reads	properly paired reads (P)	P as % of total reads	average coverage in exons	average coverage in introns	average intergenic coverage	% of reads in exons	% of reads in introns	% of reads that are intergenic
WT MEF2B-V5	216913628	38926007	193379141	89.15%	1.79E+08	82.7%	88.65	0.558	0.177	90.76	6.86	2.38
K4E MEF2B-V5	237278192	41405283	209312519	88.21%	1.94E+08	81.6%	95.99	0.583	0.185	91.06	6.64	2.30
Y69H MEF2B-V5	250039810	45073674	228750317	91.49%	2.15E+08	86.1%	106.27	0.582	0.218	91.52	6.02	2.46
D83V MEF2B-V5	236565298	42125131	212306044	89.75%	1.98E+08	83.5%	98.65	0.534	0.187	91.75	5.97	2.28
empty vector	261588114	50939870	228969442	87.53%	2.11E+08	80.8%	104.41	0.564	0.209	91.65	5.95	2.40

Supplementary Table 12: RNA-seq quality control statistics

Supplementary References

1. Anders, S. & Huber, W. Differential expression analysis for sequence count data. *Genome Biol.* **11,** R106 (2010).
2. Subramanian, A. *et al.* Gene set enrichment analysis: a knowledge-based approach for interpreting genome-wide expression profiles. *Proc. Natl. Acad. Sci. U. S. A.* **102,** 15545–15550 (2005).
3. Mootha, V. K. *et al.* PGC-1alpha-responsive genes involved in oxidative phosphorylation are coordinately downregulated in human diabetes. *Nat. Genet.* **34,** 267–273 (2003).
4. Gröger, C. J., Grubinger, M., Waldhör, T., Vierlinger, K. & Mikulits, W. Meta-analysis of gene expression signatures defining the epithelial to mesenchymal transition during cancer progression. *PloS One* **7,** e51136 (2012).
5. Zhang, Y. *et al.* Model-based analysis of ChIP-Seq (MACS). *Genome Biol.* **9,** R137 (2008).
6. Li, Q., Brown, J., Huang, H. & Bickel, P. Measuring reproducibility of high-throughput experiments. *Ann. Appl. Stat.* **5,** 1752–1779 (2011).
7. Landt, S. G. *et al.* ChIP-seq guidelines and practices of the ENCODE and modENCODE consortia. *Genome Res.* **22,** 1813–1831 (2012).
8. Bailey, T. L. & Machanick, P. Inferring direct DNA binding from ChIP-seq. *Nucleic Acids Res.* **40,** e128 (2012).
9. McLean, C. Y. *et al.* GREAT improves functional interpretation of cis-regulatory regions. *Nat. Biotechnol.* **28,** 495–501 (2010).
10. Kent, W. J. *et al.* The human genome browser at UCSC. *Genome Res.* **12,** 996–1006 (2002).

11. Wang, S. *et al.* Target analysis by integration of transcriptome and ChIP-seq data with BETA. *Nat. Protoc.* **8,** 2502–2515 (2013).

12. Chen, T.-W. *et al.* ChIPseek, a web-based analysis tool for ChIP data. *BMC Genomics* **15,** 539 (2014).
